# Variables Associated with Amputation Among 600 Community-Acquired Hand Infections—A Surgical and Infectiology Cohort

**DOI:** 10.3390/jcm15166314

**Published:** 2026-08-15

**Authors:** Camillo T. Müller, Franziska Grünfelder, Ilker Uçkay, Valentin Haug, Ulrich Kneser, Leila Harhaus, Martin Aman

**Affiliations:** 1 Clinic for Plastic, Reconstructive & Aesthetic Surgery, Evangelical Clinics Essen-Mitte, 45136 Essen, Germany; 2 BeYou.medical Sàrl Private Clinic, 1004 Lausanne, Switzerland; 3 Service de Chirurgie Plastique, Reconstructive et Esthétique et de Chirurgie de la Main, Département de L’appareil Locomoteur, Faculté de Biologie et Médecine, University of Lausanne, 1011 Lausanne, Switzerland; 4 Plastic and Reconstructive Surgery, Burn Center, Department for Plastic Surgery, BG Klinik Trauma Center Ludwigshafen, University of Heidelberg, 67071 Ludwigshafen, Germany; 5 Plastic and Reconstructive Surgery, Burn Center, Department for Hand- and Peripheral Nerve Surgery and Rehabilitation, BG Klinik Trauma Center Ludwigshafen, University of Heidelberg, 67071 Ludwigshafen, Germany; 6 Infectiology, Unit for Clinical and Applied Research, Balgrist University Hospital, University of Zurich, 8008 Zurich, Switzerland; 7 Department of Plastic, Reconstructive, and Aesthetic Surgery, Vivantes Klinikum im Friedrichshain, 10249 Berlin, Germany; 8 Department of Hand, Plastic, and Microsurgery, Burn Center, Klinikum Unfallkrankenhaus Berlin, Hand, Replantation, and Microsurgery, Charité—Universitätsmedizin Berlin, 10117 Berlin, Germany; 9 Department of Plastic and Reconstructive Surgery, Charité—Universitätsmedizin Berlin, 10117 Berlin, Germany

**Keywords:** hand infections, phlegmon, debridement, amputation, antibiotic treatment

## Abstract

**Background/Objectives:** Posttraumatic infections are common in emergency hand surgery. Amputation is the worst outcome and can occur at admission or during the course of antibiotic treatment and iterative surgical debridement. We wondered if the prolongation of antibiotic treatment beyond the usual indication, and more surgical debridement besides its immediate benefit, could reveal additional preventive effect against amputation during or immediately after therapy. **Methods:** We investigate 166 risk association (variables) of community-acquired (traumatic) hand infection with overall treatment failure using a specifically designed retrospective single-center cohort between 1 November 2018 and 31 October 2020. **Results:** Among 600 patients (362 males; 71 (11.8%) with diabetes mellitus), 58 (9.7%) required initially unplanned amputation during the therapeutic ourse. Multivariate Cox regression analysis identified only inherent risks associated with “amputation”: male sex (hazard ratio [HR] 3.12, 95% confidence interval [CI] 1.28–7.69, *p* = 0.01), age (HR 1.03, 95% CI 1.01–1.04, *p* = 0.03), diabetes (HR 2.40, 95% CI 1.15–5.01, *p* = 0.02), whereas no interventional variables such as early flapping or antibiotic-related parameters (early empirical antibiotic use, total duration of antibiotics including its initial parenteral use, and choice of agent) altered outcomes. **Conclusions:** In severe (traumatic) hand infections among 600 patients, the outcomes were determined by the extent of trauma and underlying comorbidities. The outcomes do not seem to be effectively reduced by more surgery or initial antibiotic treatment.

## 1. Introduction

Worldwide, community-acquired and traumatic hand infections are a leading indication for emergency hand surgery [1,2,3,4,5,6]. Independently of the diverse origins [3,4,5,6], the surgical indication typically involves an urgent debridement in the operating theater followed by postoperative antibiotic therapy [7]. In many episodes, the postoperative antimicrobial treatment consists of an initial parenteral administration, which is sometimes with local (intraosseous) antibiotic therapy. The prognosis is often poor, especially in elderly frail patients. Presented with these sorts of infections, surgeons and physicians want to reduce the risk of poor outcomes. This can take the form of prescribing an unnecessarily broad-spectrum regimen (often with combinations of agents), administering parenteral rather than oral therapy, or continuing therapy for a longer duration than necessary. Although the clinicians’ concern about failing to adequately treat infection is understandable, it is not only ineffective, but theoretically associated with risks of adverse events, increased costs, and promoting antibiotic resistance. Furthermore, many clinicians relying on antibiotic overuse, or early coverage, with flapping during antibiotic treatment, fail to recognize the importance of other variables inherent to the patient and the index trauma. The hand, together with the face, is widely believed to be one of the large body parts yielding optimal blood irrigation and excellent self-healing proportions. A significant subset of patients will undeniably experience surgical complications, overall treatment failures in many forms, and/or mechanical sequelae. In some cases, amputation (of one or more fingers) is necessary to prevent immediate further, possibly life-threatening complications [8,9], or it is inevitable due to massive trauma-related tissue loss or tissue destruction in the immediate aftermath of an (ischemic) trauma, but often amputation is the ultimate, elective surgery to treat anticipated treatment failures and persistent tissue loss and wound breakdowns. Additionally, optimal management of acute, trauma-related hand infections such as phlegmons requires identifying early situations at risk of amputations so as not to delay them and waste time [10].

There is only very sparse literature regarding clinical and therapeutic variables associated with wound-related amputations in hand emergency infections. Almost all are underpowered in terms of stratified analysis and main results. We therefore conducted a very large, single-center, retrospective cohort study to investigate clinical and therapeutic parameters in pyogenic bacterial hand infections that might be associated with ultimate amputation, overall management failures, and the need for soft tissue coverage. The surgical teams were all experienced hand and plastic surgeons working in a tertiary setting.

Specifically, we aimed to determine whether an increased frequency of surgical debridement and extended systemic antibiotic therapy could prevent these unplanned amputations. We focused on clinically important variables that can be, ideally, modified, instead of enumerating all theoretical, fateful variables that could be statistically associated with amputation. Similarly, this study did not address the diagnosis and treatment of nosocomial surgical site infections following elective hand surgery, the epidemiology of microbiology of hand infections, implant-related hand infections, open fractures, atypical infections, or amputations due to massive traumatic tissue destruction, ischemia, cancer, or aesthetic reasons, for which a broader literature is available [11,12,13].

## 2. Materials and Methods

### 2.1. Data Collection, Study Outcomes, and Definitions

In this retrospective cohort study, we reviewed all hospital records to identify all patients with acute hand infections at the emergency department of BGU Klinik Ludwigshafen, Germany, between 1 November 2018 and 31 October 2020. For each of the episodes, two authors (C.T.M., F.G.) assessed 166 separate variables in total, including patient demographics and comorbidities, clinical presentation, laboratory, microbiology, and detailed interventions such as overall treatment failures, number of interventions, and pre- and postoperative antibiotic therapies. The database has served for many analyses and publications. For this study, the primary outcome was the need for any amputation in the affected hand linked to the index trauma/infection, and we concentrated on clinically pertinent variables. Secondary outcomes were the need for surgical soft tissue coverage and general treatment failures at the first attempt until the time the database was closed on 30 September 2022. We also excluded hand surgeries in patients undergoing other concomitant orthopedic surgeries (e.g., polytrauma patients), chronic infections without a recent onset, and very mild infections that were treated non-operatively (e.g., by the General Practitioner). We purposely focused on community-acquired infections and excluded clear (late) surgical site infections following an uninfected index surgery performed for trauma. However, we cannot entirely rule out the attribution of the immediate medical outpatient intervention of healthcare workers or laypeople on hand. Every patient was included once (first episode) for infection in the same hand. However, they could have had non-infected hand trauma in the past.

**Figure 1:**
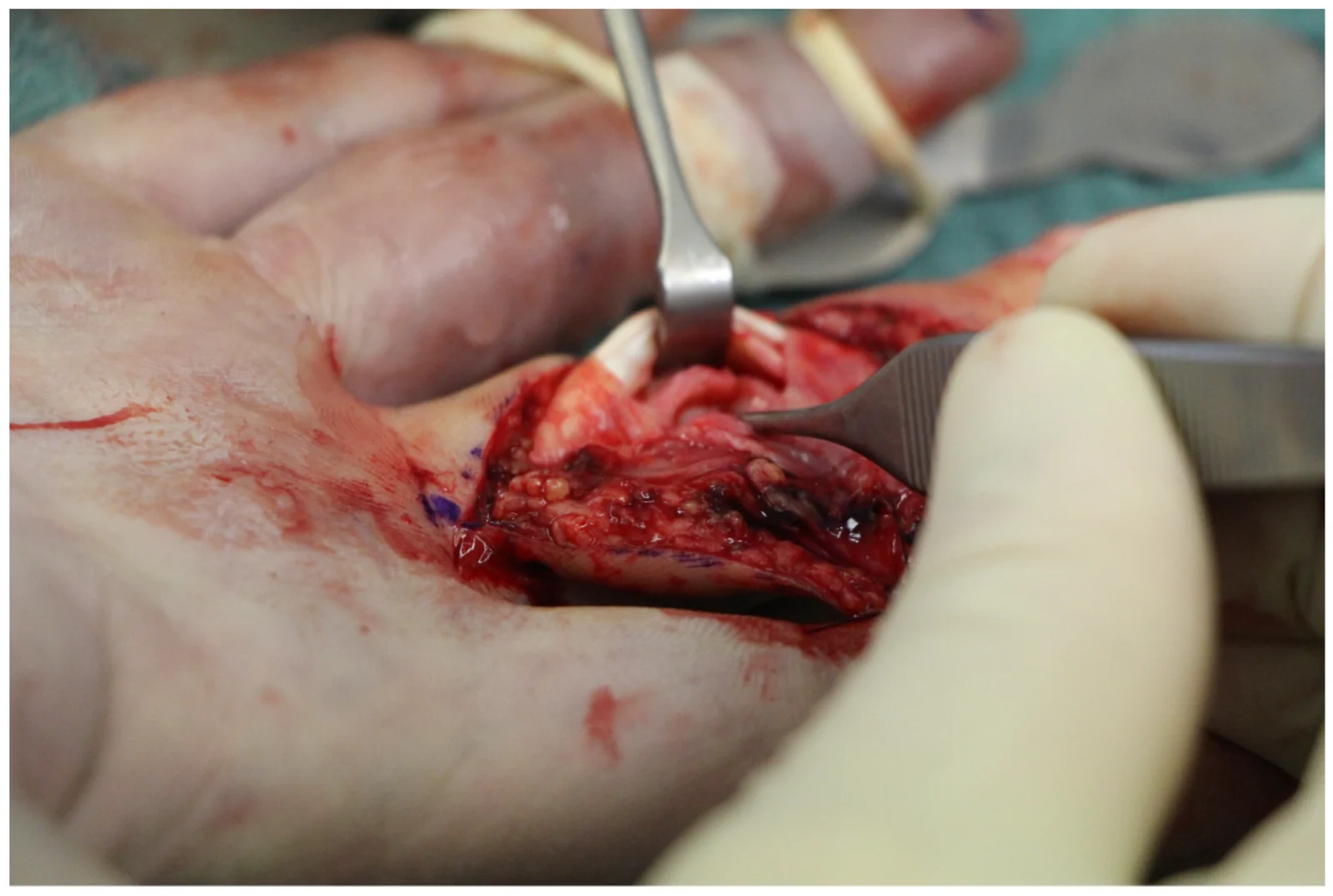
Right hand, proximal phalanx of the middle finger (D3): intraoperative view after irrigation of the tendon sheath: white tendon (flexor digitorum superficialis 4 and flexor digitorum profundus 4) presented on hook, rose-colored intact neurovascular bundle (NVB) identified and protected at the wound edge. Photo published with the permission of the patient.

We diagnosed a bacterial hand infection clinically [14] by the presence of new clinical signs of infection: redness, warmth, pus, pain, rapidly spreading symptoms, lymphangitis, and impaired function after a cause such as trauma. This diagnosis was supported by the presence of the same pathogen in at least two intraoperative microbiological samples, a plausible history, an infectious-disease clinician consultation, the need for surgical intervention(s) for infection, and systemic antibiotic treatment based on the susceptibility testing of the causative bacteria. Radiological suspicion and corresponding histopathology were facultative. As we allowed empirical antibiotic treatment before the first surgical debridement, we ignored quantifying microbiological cultures or the necessity for a visible Gram staining [15]. We interpreted every antibiotic spectrum larger than the most commonly used amoxicillin–clavulanic acid as broad spectrum, whether monotherapies, concomitant antibiotic combinations, or broad spectrum in sequential use. Broad-spectrum therapies also included gentamicin that was occasionally used as local, intraosseous treatment. We defined “amputation” as the surgical act of deliberately resecting anatomical parts of the hand (part of or full fingers, rays including metacarpals). A “therapeutic failure” was based on clinical grounds of the treating clinicians requiring re-intervention. “Soft tissue” coverage was any surgical act that was covering an open wound (except for negative-pressure devices that we avoided for infection) (Figure 1).

### 2.2. Statistical Analyses

The primary objective and the primary outcome were the assessment of 166 demographic, laboratory, and interventional variables associated with ultimate (elective) amputation. Secondary outcomes were overall therapeutic failures and the need for soft tissue coverage during or immediately after the index infection. The targeted sample size was arbitrarily set at 600 independent infection episodes to avoid underpowering of possible stratified analyses and to enable multivariate adjustments. We performed group comparisons using the Pearson χ^2^, Fisher exact, or Wilcoxon rank-sum-tests. Three sperate Cox regression analyses with three study outcomes each were adjusted for the large case mix: without matching or cluster-effect analyses. The endpoints were the end date of the surveillance period, unplanned amputations during or immediately after the therapeutic course, death, or the occurrence of any of the corresponding outcomes. In these multivariate analyses, we focused on independent variables associated with medical plausibility and clinical importance and avoided including less important parameters based on mere computation. We preferred Cox regression analysis (instead of a logistic regression) because of the short and individualized study participation time of the study patients, entering and leaving at different time points over the large study period, although we cannot guarantee the formal proportional hazard assumption in the inherently dynamic process of acute infections (and their iterative debridement).

The database was complete without the necessity for imputation regarding our study objective. We computed the antibiotic durations (that were ultimately effective against the pathogens) as continuous and as stratified variables, arbitrarily cutting the variables “total antibiotic duration” into three equivalently sized thirds (≤7 days, 8 to ≤14 days, and ≥14 days). Likewise, we regrouped the leading bacteria into three clinically important groups of *Staphylococcus aureus*, streptococci, and Gram-negative pathogens [16]. In contrast, we could not group the localizations and tissues of infection as we could have done after minor trauma [17]. Most posttraumatic infections involved soft tissues, tendons, muscles, bone and joints to various extents, depth, ischemia, and compositions.

Regarding our final multivariate models and to avoid overfitting, we only included clinically pertinent, important, and frequently represented and reproducible variables that each showed substantial prevalence in the entire database in our final model, i.e., not seldom. Formally and statistically, we introduced these clinically approved variables, particularly surgical antibiotic-related variables, ideally with a univariate *p*-value of <0.02, stepwise into the multivariate model. We checked all independent variables for confounding, colinearity, and interaction. We used STATA™ software (version 15, College Station, TX, USA) and considered *p*-values < 0.05 (two-tailed) as significant.

## 3. Results

### 3.1. Study Population and Infections

We included 600 episodes of acute hand infection in 600 patients requiring surgery, including 362 males (60.3%) with a median age of 56 years (interquartile range 39–67 years). In total, 71 patients (11.8%) were diabetic with an overall median body mass index of 26 kg/m^2^ (interquartile range 22–29 kg/m^2^). Other chronically immunosuppressed states were advanced renal insufficiency (*n* = 40), systemically active cancer (*n* = 3), rheumatic disease with immunosuppressive drugs (*n* = 20), and advanced liver diseases (*n* = 15). Beyond immunosuppression, noteworthy comorbidities were symptomatic cardiovascular diseases (*n* = 217; 36%), asthma or chronic obstructive pulmonary disease (COPD, *n* = 15), and recurrent gout (*n* = 20). Infections involved the right (dominant) hand in 299 cases (50%), and 159 cases (25%) were attributed to bite injuries. In 63 episodes (10%), we removed foreign bodies during debridement. Hand trauma was not new for 76 of these patients (76/600; 13%) who were treated for a (non-infected) affection in prior lifetime. The subset of patients largely overlapped with the study patients with psychiatric disorders (*n* = 49) or self-declared addictions (*n* = 72).

Clinically, 41 patients (6.8%) presented with a purulent joint infection and 24 (4%) with a purulent tendon synovitis. Lymphangitis was present in 86 cases (14%). Only nine patients (1.5%) showed daily fever (≥ 38 °C axillary) despite anti-inflammatory (pain) medication and empirical antibiotic therapy that was started preoperatively (113 episodes; 19%). The infections were acute and severe, but remained local. Only ten episodes (1.7%) revealed systemic signs of a (potentially generalized) infection. Among 41 very diverse microbiological constellations, the three most frequent pathogen groups were *S. aureus* in 178 episodes (30%), *Streptococcus pyogenes* in 95 cases (16%), and Gram-negative bacteria in 78 cases (13%). Notably, we witnessed no cases of necrotizing fasciitis.

### 3.2. Combined Antibiotic and Surgical Treatment

All patients received at least one surgical debridement with empirical systemic antibiotic treatment after debridement and a switch to targeted agents after the availability of the microbiological culture results. Postoperative wound care, surgical visits, occupational therapy, and therapeutic elevation of the hand occurred daily during a median length of hospital stay of 4 days (interquartile range 3–7 days). The median duration of complete hand immobilization was 3 days.

Infectious-disease physicians prescribed a total of 92 empirical or targeted regimens, of which 85 episodes (85/600; 14.2%) were with broad-spectrum agents. Additionally, the surgeons introduced a local, intraosseous antibiotic during 22 interventions. The median duration of microbiologically susceptible postoperative antibiotic treatment was 8 days (range 1–72 days), with a median of 4 days parenterally. We started to count postsurgical antibiotic duration with the first debridement or the first day of effective antibiotic use regarding the susceptibility testing of the causative pathogens. We could not reconstruct retrospectively the exact rationale for continuous parenteral use for every episode. We think that in most cases, this prolongation was likely due to the choice of antibiotic agents that are only available in intravenous formulations. Unusual infections were accompanied by infectious-disease physicians. No patient died because of systemic infection, and no secondary amputation was due to infection alone.

Surgically, the far majority were emergency interventions regarding first debridement (524/600; 87.3%), with removal of infected and/or necrotic soft tissues in 544 (90.7%) cases, of bone in 20 (3.3%) first interventions, and lavage of joints in nine episodes (1.5%). The surgeons were able to primarily close all wounds at the end of the first debridement in 110 episodes (110/600; 18%). In sum, 130 patients (22%) underwent at least two debridements (range two to nine), all at the initially operated site. No patient developed infection propagation to other digits or hand compartments. Specifically, 44 patients (7%) underwent two interventions, 18 patients (3%) had three interventions, 8 patients (1%) had four surgeries, and 5 patients (1%) underwent five or more surgeries for the same infection and wound problems during the first treatment episode.

### 3.3. Outcomes

Only three patients (3/600; 0.5%) experienced a true infection recurrence with the same pathogen(s) as in the index episode. These three relapses were due to *S. aureus* (*n* = 2) and *Streptococcus* spp. (*n* = 1). The 107 therapeutic failures (17.8%) at first attempt were wound-related. Overall, 48 patients (8%) required reconstructive surgery for soft tissue closure. In total, 58 patients (9.7%) underwent an ultimate amputation, with 53 amputations occurring at the finger level (partial or complete) and 5 at the metacarpal or carpal level. These patients underwent a mean of two debridement surgeries before amputation. Among the 58 ultimate amputations, 15 (15/58; 26%) required “re-amputation” at the same site. No patient developed non-resected secondary osteitis, and none required revascularization. Two patients died due to reasons other than surgery or prior infection.

### 3.4. Multivariate Adjustments

To account for differences observed in the crude group comparisons (Table 1), we performed at least three multivariate analyses to adjust for the large case mix and ran them several times. For the primary outcome “amputation” (Table 2), our final multivariate analysis identified four inherent variables factors significantly associated with the outcome “amputation”: male sex (hazard ratio [HR] 3.12, 95% confidence interval [CI] 1.28–7.69), advanced age (HR 1.03, 95% CI 1.01–1.04), diabetes (HR 2.40, 95% CI 1.15–5.01), and the number of surgical debridements prior to amputation (HR 1.31, 95% CI 1.05–1.62). Of note, none of the surgical or antibiotic-related variables or parameters of infection severity altered the risk of amputation (Table 1 and Table 2). The corresponding multivariate results for overall antibiotic duration yielded an HR of 1.00 (95% CI 0.98–1.06). The goodness-of-fit test was insignificant (*p* = 0.54). The formal, minimal receiver operating characteristic curve (ROC) value was 0.80, indicating acceptable accuracy for our final model.

The multivariate results for the secondary outcomes “soft tissue coverage” and “overall therapeutic failure” are not displayed in the tables. The significant variables for soft tissue coverage were only wound breakdown and/or persistent dehiscence (HR 44.1; 95% CI 18.4–105.7). Regarding overall failures, the corresponding significant variables were the increasing number of debridements (HR 1.56; 95% CI 1.36–1.79), an increasing age (HR 1.02; 95% CI 1.00–1.04), and, tendentially, the presence of diabetes (HR 1.68; 95% CI 0.97–2.90). Female sex was inversely associated with overall “failures” (HR 0.48; 95% CI 0.28–0.84). Supplementary File S1 covers the initial key results before the revision, and yields more analyses than the text.

## 4. Discussion

Using a very large single-center university database, we investigated the presence of (modifiable) clinical variables in community-acquired posttraumatic and operated severe acute hand infections in German patients. The variables identified were advanced age, male sex, and diabetes mellitus. None was modifiable. Notably, other parameters such as body mass index, pathogen groups, bone or tendon resection, presence of pus, or primary arthrodesis were unrelated to future amputation. Regarding the modifiable variables, the duration and modalities of postsurgical systemic antibiotic treatment failed to alter the risk of ultimate amputation, leaving space to comply with infectiology guidance and to avoid unnecessary, futile exaggerations of antimicrobial therapy. Especially the empirical use of broad-spectrum agents (beyond amoxicillin–clavulanic acid) is unnecessary for community-acquired hand infections in Central Europe, independently of antibiotic use prior to the first debridement. Indeed, the proportion of initial (empirical) broad-spectrum use in later amputated and non-amputated patients was exactly 14% in both groups.

Our findings align with previous studies on joint [17] and tendon infections [8]. Of note and importantly, our study involved only operated patients by excluding strictly conservative treatments [18,19]. Despite a large sample of 600 patients stretching over a recruitment period of two and a half years, we cannot entirely exclude a minimal periodical bias that could externally influence our outcomes. In contrast, our sample size completely avoids underpowering inherent to many retrospective surgical studies involving a large case mix of investigated problems.

Regarding wound problems after surgery [20], various biopsychosocial factors impact the perioperative complication rate in the frail elderly [21], with physiological changes and wound breakdowns being most significant [22]. Skin atrophy and joint degeneration predispose indirectly (via skin breakdown) to infections and worse clinical outcomes. Shortened telomeres may lead to a deficiency in the adaptive immune system [23] and collagen synthesis. Dementia or depression an be comorbidities leading to delayed consultation and poor or no compliance with wound-related recommendations.

Male sex is another risk factor for worse outcomes in many fields of infectious diseases due to behavioral and hormonal factors [24]. Besides compliance issues, the adaptive immune response might be more efficient in females, especially premenopausal women, due to a higher antibody response [25] facilitated by a greater number of B cells and a broader B-cell repertoire [26]. Additionally, women might have a higher CD4/CD8 ratio, higher CD4 counts, and a greater number of activated T cells [25,26,27,28].

Diabetes is genuinely associated with an increased risk of infection, particularly soft tissue infections [29,30], across the entire field of orthopedic surgery [31], including hand surgery [32,33,34,35,36,37,38,39,40]. This increased risk can be attributed to persistent hyperglycemia [41], impaired immune function [41], neuropathy, and arteriopathy [42]. Impaired immune function in diabetes is due to reduced neutrophil function, decreased phagocytosis, and reduced T-cell activity, increasing the risk of complicated progression. Neuropathy can cause decreased sensation in the hands, making injuries and infections harder to detect and potentially masking increasing pain during progression and the intrusion of foreign bodies. Unsurprisingly, the literature reports many challenging hand infections in diabetic patients, often associated with finger amputations [33,35,36,39,43].

In many retrospective analyses, multiple surgeries were associated with more severe outcomes and not predictive of them [44,45]. There is no direct causative relationship. These phenomena, known as “confounding by indication,” or more generally time-related biases, are inherent to all retrospective study designs. This reflects that surgeons, aware of an unsatisfactory healing course, try to circumvent it with aggressive debridement. Hence, and unsurprisingly, patients needing multiple debridements also needed amputation, while longer antibiotic prescription could not prevent, or reverse, the fate of an ultimate amputation. This said, this hallmark of “confounding by indication” does not mean that it cannot be analyzed or interpreted. In our study, surgical debridement and antibiotics were certainly very effective as long as they were prescribed for the clinical need. They had therapeutic benefits, but did not show any visible additional preventive benefit beyond their true indications. In other words, more debridement and more antibiotics can be explained by “confounding by indication,” but we can comfortably say that additional (excessive) interventions did benefit, even in retrospective study designs. Otherwise, we would have seen this “potential benefit” in numbers, which would not have passed undetected among 600 infection episodes.

Our study has many strengths and limitations. The strengths are the largest database of hand trauma infections in the available literature, but we refrained from overfitting with all of them. We equally confined our search to variables that remain clinically important for our study question, so avoiding underpowering of our final model. The limitations include its retrospective and single-center design, which restricts the generalizability of our findings to other settings, resource-poor (tropical) settings [4]. Additionally, the decision to amputate was made by the treating surgeons based on clinical findings. This could introduce an overestimation bias towards amputation or result in unnecessary amputations for patients with more severe symptoms. Despite these theoretical limitations, our study includes the largest number of hand infections in the literature at a tertiary referral center, which reduces the likelihood of a relevant “surgeon bias.”

Other formal limitations are the assessment of associations with unplanned amputation in hand surgery, and not with general variables. For example, preexisting chronic vascular disease or long-lasting active smoking are major risk factors for amputation in non-trauma patients. We did not assess arteriopathy as a relevant parameter because of the clinical paucity of its presence among young hand trauma patients. In line with our focus on important variables, we skipped performing a formal statistical evaluation of “prediction of amputation,” which would require a complete clinical model, internal and external validation, calibration curves, decision-curve analyses, construction of a risk score, and visualization and eventually machine learning. Such an analysis was beyond our study aim [46]. Similarly, posttraumatic amputation following injured hand parts are a hallmark of hand surgery. This risk is heavily related to the initial injury independently of antibiotic therapy and surgery. In this study, we were not interested in the anatomical regions destructed, which might motivate another paper in the field of occupational health [47]. In a pure hand surgery paper, we were able to add other outcomes such as infection persistence, wound breakdown, recurrent infection with new or relapsing pathogens, the need for repeat debridement, the need for reconstruction by flaps and occupational health issues, and return to work. These are objects of other publications inasmuch as each of these possible alternative outcomes requires an entirely new analysis.

## 5. Conclusions

We report a large single-center cohort of 600 patients who underwent surgery for acute community-acquired hand infections in Germany. A total of 58 patients, corresponding to 9.7% of the cohort, ultimately required amputation. Male sex, increasing age, diabetes, and the number of debridements were associated with amputation (via confounding by indication), whereas antibiotic-related variables, including preoperative antibiotic use, broad-spectrum therapy, route, and duration of postoperative antibiotic treatment, were not. Amputation after severe hand infection appears to be driven mainly by patient-related and trauma-related factors rather than by escalation of antibiotic treatment or repeated surgical intervention. The latter were not associated with lower amputation risk in this retrospective cohort. Hence, there is a strong message in favor of better antibiotic and surgical stewardships: theoretically, and biostatistically, only prospective randomized-controlled trials could best confirm our findings, which however are difficult to conduct in an ethically acceptable way.

## Figures and Tables

**Table 1:** Patients with hand infections with and without amputation.

	Ultimate Amputation	Comparison	Without Amputation
*n* = 600	*n* = 58	*p* Value *	*n* = 542
**Patient characteristics**			
Female sex	14 (24.1%)	0.01	224 (41.3%)
Median age Median body mass index	59 years	0.00	53 years
	26.4 kg/m^2^		26.0 kg/m^2^
Diabetes mellitus	17 (29.3%)	0.00	54 (10.0%)
**Surgical parameters**			
Median length of surgery	30.24 min	0.01	25.92 min
Median number of surgical interventions	2	0.00	1
Primary arthrodesis	1 (1.7%)		8 (1.5%)
Open wound at admission	15 (25.9%)	0.00	58 (10.7%)
Visual pus in joint	8 (13.8%)	0.03	33 (6.1%)
**Key pathogens**			
*Staphylococcus aureus*	29 (50.0%)	0.00	149 (27.5%)
*Streptococcus pyogenes*	11 (19.0%)		84 (15.5%)
Gram-negative pathogens	11 (19.0%)		67 (12.4%)
**Antibiotic treatment**			
Antibiotic use prior to first debridement	9 (15.5%)		104 (19.2%)
Median duration of postoperative use	10 days	0.03	8 days
- <7 days	10 (17.2%)		180 (33.2%)
- 7–14 days	10 (17.2%)		118 (21.8%)
- >14 days	38 (65.5%)		244 (45.0%)
Broad-spectrum antibiotic use *	8 (13.8%)		77 (14.2%)

* Agents or combinations broader than the most common oral empirical agent; amoxicillin–clavulanic acid.

**Table 2:** Multivariate Cox regression adjustment: main outcome “amputation.”

	Univariate Results	Multivariate Results
*n* = 600	Hazard Ratio (95% CI)	Hazard Ratio (95% CI)
**Patient characteristics**		
Female sex	**0.46 (0.25–0.83)**	**0.32 (0.13–0.78)**
Age (continuous variable)	**1.02 (1.01–1.04)**	**1.03 (1.01–1.04)**
Presence of antibiotic therapy prior to surgery	1.91 (0.55–6.66)	n.d.
Presence of diabetes mellitus	**3.91 (2.22–6.90)**	**2.40 (1.15–5.01)**
**Therapy and intraoperative findings**		
Number of surgical interventions	**1.35 (1.18–1.54)**	**1.31 (1.05–1.62)**
Use of local gentamycin	**2.76 (1.18–6.45)**	n.d.
Bone resection	1.24 (0.92–1.67)	n.d.
Wound dehiscence at admission	**2.70 (1.50–4.87)**	n.d.
Pus in joint	**0.44 (0.21–0.93)**	1.12 (0.42–2.98)
Primary arthrodesis	**0.13 (0.06–0.31)**	n.d.
Duration of postoperative antibiotic treatment	1.00 (0.98–1.06)	1.00 (0.98–1.06)
**Important pathogens groups**		
*Staphylococcus aureus*	**2.36 (1.41–3.97)**	1.88 (0.90–3.93)
*Streptococcus pyogenes*	0.97 (0.50–1.88)	1.01 (0.40–2.59)
Gram-negative pathogens	1.38 (0.72–2.67)	1.11 (0.37–3.32)
Infection recurrence	1.68 (0.22–12.60)	n.d.

Variables **in bold** are statistically significant (two-tailed *p*-value < 0.05). n.d. = not done due to lack of clinical reasons, lack of proportional hazard assumptions, or because of insignificant associations.

## Data Availability

Supplementary File S1 represents the first do-file, resuming the statistical methods, their results, and analyzed variables in one single PDF file. The abbreviations are self-explanatory. The software commands correspond to the usual STATA™ do-files.

## Supplementary Materials

The following supporting information can be downloaded at https://www.mdpi.com/article/10.3390/jcm15166314/s1. The do-file of the first (initial) key analyses of the cohort (File S1). During both revisions, the parameters of interest changed. File S1 is a selection for this paper. The general study database is much larger, more surgical, and is not displayed in this publication.
