# Supplementary material for: Variables Associated with Amputation Among 600 Community-Acquired Hand Infections—A Surgical and Infectiology Cohort
Source: J Clin Med. 2026 Aug 15;15(16):6314. doi: 10.3390/jcm15166314 (PMC13513049; doi:10.3390/jcm15166314)
Supplement: Supplementary file 1 [file jcm-15-06314-s001.zip › jcm-4403058-supplementary.pdf]

```
                                                    ___  ____  ____  ____  ____(R)
                                                   /__    /   ____/   /   ____/
                                                  ___/   /   /___/   /   /___/
                                                    Statistics/Data Analysis

-------------------------------------------------------------------------------
      name:  <unnamed>
       log:  D:\camillofranziska.smcl
  log type:  smcl
 opened on:  31 Oct 2022, 16:10:40

 1 .
 2 . /*
   > Objectif: Arbeit Franziska Handphlegmonen - September 2022 (Analyse Ilker Uçk
   > ay)
   > */
 3 .
 4 . insheet using D:\camillofranziska.txt
   (166 vars, 600 obs)

 5 . compress
    (0 bytes saved)

 6 . count
    600

 7 .
 8 . ** Aussschluss nicht analysierter Einzelvariablen
 9 . drop patient opdauervon opdauerbis dateop2 dateop3 dateop4 dateop5

10 . drop dateop6 dateop7 dateop8 dateop9 fingerseite3 datenotfall

11 . drop ab1von ab1bis ab2von ab2bis ab3von

12 . drop ab3bis ab4von ab4bis flucanozol dauerab5 abvon abbis

13 . *drop 4Finger1linkD5PIP
14 . count
    600

15 .
16 . *** DESKRIPTIVES
17 . ** Patienten-Stammdaten
18 . codebook sex wohnort versicherung beruf zustaendigebg diabetes cardiovask ast
   > hmacopd

-------------------------------------------------------------------------------
sex                                                                 (unlabeled)
-------------------------------------------------------------------------------

                  type:  numeric (byte)

                 range:  [1,2]                        units:  1
         unique values:  2                        missing .:  0/600

            tabulation:  Freq.  Value
                           362  1
                           238  2

-------------------------------------------------------------------------------
wohnort                                                             (unlabeled)
-------------------------------------------------------------------------------

                  type:  numeric (byte)

                 range:  [0,3]                        units:  1
         unique values:  4                        missing .:  1/600

            tabulation:  Freq.  Value
                             5  0
                           194  1
                           397  2
                             3  3
                             1  .
```

versicherung (unlabeled)

```
            type:  numeric (byte)

           range:  [1,6]                        units:  1
   unique values:  6                        missing .:  2/600

      tabulation:  Freq.  Value
                      80  1
                     104  2
                     407  3
                       1  4
                       5  5
                       1  6
                       2  .
```

beruf (unlabeled)

```
            type:  numeric (byte)

           range:  [0,16]                       units:  1
   unique values:  14                       missing .:  1/600

            mean:   1.54257
        std. dev:   3.58104

     percentiles:        10%       25%       50%       75%       90%
                           0         0         0         0         5
```

zustaendigebg (unlabeled)

```
            type:  numeric (byte)

           range:  [1,13]                       units:  1
   unique values:  13                       missing .:  498/600

            mean:   5.85294
        std. dev:   2.93628

     percentiles:        10%       25%       50%       75%       90%
                           3         4         5         7        11
```

diabetes (unlabeled)

```
            type:  numeric (byte)

           range:  [0,1]                        units:  1
   unique values:  2                        missing .:  0/600

      tabulation:  Freq.  Value
                     529  0
                      71  1
```

cardiovask (unlabeled)

```
            type:  numeric (byte)

           range:  [0,1]                        units:  1
   unique values:  2                        missing .:  0/600
```

```
            tabulation:  Freq.  Value
                           383  0
                           217  1

-------------------------------------------------------------------------------
asthmacopd                                                          (unlabeled)
-------------------------------------------------------------------------------

                  type:  numeric (byte)

                 range:  [0,3]                        units:  1
         unique values:  4                        missing .:  0/600

            tabulation:  Freq.  Value
                           570  0
                            15  1
                            14  2
                             1  3

19 . codebook rheuma gicht niereninsuffizienz leber psychiatrie immun tumor sucht
> thyreose

-------------------------------------------------------------------------------
rheuma                                                              (unlabeled)
-------------------------------------------------------------------------------

                  type:  numeric (byte)

                 range:  [0,1]                        units:  1
         unique values:  2                        missing .:  0/600

            tabulation:  Freq.  Value
                           582  0
                            18  1

-------------------------------------------------------------------------------
gicht                                                               (unlabeled)
-------------------------------------------------------------------------------

                  type:  numeric (byte)

                 range:  [0,1]                        units:  1
         unique values:  2                        missing .:  0/600

            tabulation:  Freq.  Value
                           571  0
                            29  1

-------------------------------------------------------------------------------
niereninsuffizienz                                                  (unlabeled)
-------------------------------------------------------------------------------

                  type:  numeric (byte)

                 range:  [0,1]                        units:  1
         unique values:  2                        missing .:  0/600

            tabulation:  Freq.  Value
                           560  0
                            40  1

-------------------------------------------------------------------------------
leber                                                               (unlabeled)
-------------------------------------------------------------------------------

                  type:  numeric (byte)

                 range:  [0,9]                        units:  1
         unique values:  7                        missing .:  5/600
```

```
            tabulation:  Freq.  Value
                           579  0
                             4  1
                             8  2
                             1  3
                             1  4
                             1  5
                             1  9
                             5  .
```

---

psychiatrie **(unlabeled)**

```
                  type:  numeric (byte)

                 range:  [0,4]                        units:  1
         unique values:  5                        missing .:  0/600

            tabulation:  Freq.  Value
                           531  0
                            19  1
                            34  2
                             3  3
                            13  4
```

---

immun **(unlabeled)**

```
                  type:  numeric (byte)

                 range:  [0,2]                        units:  1
         unique values:  3                        missing .:  0/600

            tabulation:  Freq.  Value
                           569  0
                             4  1
                            27  2
```

---

tumor **(unlabeled)**

```
                  type:  numeric (byte)

                 range:  [0,1]                        units:  1
         unique values:  2                        missing .:  0/600

            tabulation:  Freq.  Value
                           597  0
                             3  1
```

---

sucht **(unlabeled)**

```
                  type:  numeric (byte)

                 range:  [0,4]                        units:  1
         unique values:  5                        missing .:  0/600

            tabulation:  Freq.  Value
                           520  0
                            48  1
                            16  2
                            14  3
                             2  4
```

```
------------------------------------------------------------------------------
thyreose                                                           (unlabeled)
------------------------------------------------------------------------------

                  type:  numeric (byte)

                 range:  [0,1]                        units:  1
         unique values:  2                        missing .:  0/600

            tabulation:  Freq.  Value
                           539  0
                            61  1

20 . centile age, c (25,50,75)

                                                       -- Binom. Interp. --
    Variable |       Obs  Percentile    Centile        [95% Conf. Interval]
-------------+-------------------------------------------------------------
         age |       600         25          39              37          42
             |                   50          54              52          55
             |                   75          67              64          69

21 . centile bmi, c (25,50,75)

                                                       -- Binom. Interp. --
    Variable |       Obs  Percentile    Centile        [95% Conf. Interval]
-------------+-------------------------------------------------------------
         bmi |       507         25        22.9            22.5        23.4
             |                   50          26            25.6        26.6
             |                   75        29.5              29        30.4

22 . drop wohnort versicherung beruf zustaendigebg cardiovask asthmacopd rheuma gi
> cht

23 . drop niereninsuffizienz leber psychiatrie immun tumor sucht thyreose

24 . 
25 . ** Infektionen - Klinik und Labor
26 . codebook anamnese punktionhandgelenk fremdkoerper beugesehne eitersehne

------------------------------------------------------------------------------
anamnese                                                           (unlabeled)
------------------------------------------------------------------------------

                  type:  numeric (byte)

                 range:  [0,25]                       units:  1
         unique values:  24                       missing .:  0/600

                  mean:     4.275
              std. dev:   3.86794

           percentiles:        10%       25%       50%       75%       90%
                                 3         3         3         3         8

------------------------------------------------------------------------------
punktionhandgelenk                                                 (unlabeled)
------------------------------------------------------------------------------

                  type:  numeric (byte)

                 range:  [0,3]                        units:  1
         unique values:  4                        missing .:  0/600

            tabulation:  Freq.  Value
                            48  0
                            31  1
                           503  2
                            18  3
```

```
-------------------------------------------------------------------------------
fremdkoerper                                                        (unlabeled)
-------------------------------------------------------------------------------

                  type:  numeric (byte)

                 range:  [1,2]                        units:  1
         unique values:  2                        missing .:  0/600

            tabulation:  Freq.  Value
                            63  1
                           537  2

-------------------------------------------------------------------------------
beugesehne                                                          (unlabeled)
-------------------------------------------------------------------------------

                  type:  numeric (byte)

                 range:  [1,2]                        units:  1
         unique values:  2                        missing .:  0/600

            tabulation:  Freq.  Value
                           101  1
                           499  2

-------------------------------------------------------------------------------
eitersehne                                                          (unlabeled)
-------------------------------------------------------------------------------

                  type:  numeric (byte)

                 range:  [1,2]                        units:  1
         unique values:  2                        missing .:  0/600

            tabulation:  Freq.  Value
                            24  1
                           576  2

27 . codebook eitersubkutan eitergelenk karpaltunnel trauma biss

-------------------------------------------------------------------------------
eitersubkutan                                                       (unlabeled)
-------------------------------------------------------------------------------

                  type:  numeric (byte)

                 range:  [1,2]                        units:  1
         unique values:  2                        missing .:  0/600

            tabulation:  Freq.  Value
                           239  1
                           361  2

-------------------------------------------------------------------------------
eitergelenk                                                         (unlabeled)
-------------------------------------------------------------------------------

                  type:  numeric (byte)

                 range:  [1,2]                        units:  1
         unique values:  2                        missing .:  0/600

            tabulation:  Freq.  Value
                            41  1
                           559  2

-------------------------------------------------------------------------------
karpaltunnel                                                        (unlabeled)
-------------------------------------------------------------------------------
```

```
                 type:  numeric (byte)

                range:  [1,2]                        units:  1
        unique values:  2                        missing .:  0/600

          tabulation:  Freq.  Value
                          44  1
                         556  2
```

---

trauma **(unlabeled)**

```
                 type:  numeric (byte)

                range:  [0,8]                        units:  1
        unique values:  9                        missing .:  0/600

          tabulation:  Freq.  Value
                         105  0
                          75  1
                         111  2
                          43  3
                           7  4
                          28  5
                           1  6
                         176  7
                          54  8
```

---

biss **(unlabeled)**

```
                 type:  numeric (byte)

                range:  [0,10]                       units:  1
        unique values:  7                        missing .:  0/600

          tabulation:  Freq.  Value
                          98  0
                         343  1
                          45  2
                         103  3
                           7  4
                           3  5
                           1  10
```

28 . tab anamnesezusammengefasst, m

anamnesezusammengefasst	Freq.	Percent	Cum.
1	465	77.50	77.50
2	26	4.33	81.83
3	23	3.83	85.67
4	15	2.50	88.17
5	71	11.83	100.00
Total	600	100.00	

```
29 . codebook druckschmerz lymphangitis fieber pus hautgeschlossen lyse fingerseit
> e1

-------------------------------------------------------------------------------
druckschmerz                                                        (unlabeled)
-------------------------------------------------------------------------------

                  type:  numeric (byte)

                 range:  [1,3]                        units:  1
         unique values:  3                        missing .:  0/600

            tabulation:  Freq.  Value
                            80  1
                           519  2
                             1  3

-------------------------------------------------------------------------------
lymphangitis                                                        (unlabeled)
-------------------------------------------------------------------------------

                  type:  numeric (byte)

                 range:  [1,2]                        units:  1
         unique values:  2                        missing .:  0/600

            tabulation:  Freq.  Value
                            86  1
                           514  2

-------------------------------------------------------------------------------
fieber                                                              (unlabeled)
-------------------------------------------------------------------------------

                  type:  numeric (byte)

                 range:  [1,2]                        units:  1
         unique values:  2                        missing .:  0/600

            tabulation:  Freq.  Value
                             9  1
                           591  2

-------------------------------------------------------------------------------
pus                                                                 (unlabeled)
-------------------------------------------------------------------------------

                  type:  numeric (byte)

                 range:  [1,2]                        units:  1
         unique values:  2                        missing .:  0/600

            tabulation:  Freq.  Value
                           137  1
                           463  2

-------------------------------------------------------------------------------
hautgeschlossen                                                     (unlabeled)
-------------------------------------------------------------------------------

                  type:  numeric (byte)

                 range:  [1,2]                        units:  1
         unique values:  2                        missing .:  0/600

            tabulation:  Freq.  Value
                           110  1
                           490  2
```

```
-------------------------------------------------------------------------------
lyse                                                                (unlabeled)
-------------------------------------------------------------------------------

                  type:  numeric (byte)

                 range:  [1,4]                        units:  1
         unique values:  4                        missing .:  0/600

            tabulation:  Freq.  Value
                            21  1
                            31  2
                             5  3
                           543  4

-------------------------------------------------------------------------------
fingerseite1                                                        (unlabeled)
-------------------------------------------------------------------------------

                  type:  numeric (byte)

                 range:  [1,2]                        units:  1
         unique values:  2                        missing .:  1/600

            tabulation:  Freq.  Value
                           301  1
                           298  2
                             1  .

30 . codebook lokalisation1 mcp1 pip1 dip1 gg1 mg1 eg1 fingerseite2 lokalisation2
   > mcp2

-------------------------------------------------------------------------------
lokalisation1                                                       (unlabeled)
-------------------------------------------------------------------------------

                  type:  numeric (byte)

                 range:  [0,13]                       units:  1
         unique values:  12                       missing .:  0/600

                  mean:     4.255
              std. dev:   2.87002

           percentiles:        10%       25%       50%       75%       90%
                                 1         2         3         6         9

-------------------------------------------------------------------------------
mcp1                                                                (unlabeled)
-------------------------------------------------------------------------------

                  type:  numeric (byte)

                 range:  [0,1]                        units:  1
         unique values:  2                        missing .:  0/600

            tabulation:  Freq.  Value
                           577  0
                            23  1

-------------------------------------------------------------------------------
pip1                                                                (unlabeled)
-------------------------------------------------------------------------------

                  type:  numeric (byte)

                 range:  [0,1]                        units:  1
         unique values:  2                        missing .:  0/600
```

```
         tabulation:  Freq.  Value
                        558  0
                         42  1
```

---

dip1 **(unlabeled)**

```
               type:  numeric (byte)

              range:  [0,1]                        units:  1
      unique values:  2                        missing .:  0/600

         tabulation:  Freq.  Value
                        545  0
                         55  1
```

---

gg1 **(unlabeled)**

```
               type:  numeric (byte)

              range:  [0,1]                        units:  1
      unique values:  2                        missing .:  0/600

         tabulation:  Freq.  Value
                        382  0
                        218  1
```

---

mg1 **(unlabeled)**

```
               type:  numeric (byte)

              range:  [0,1]                        units:  1
      unique values:  2                        missing .:  0/600

         tabulation:  Freq.  Value
                        391  0
                        209  1
```

---

eg1 **(unlabeled)**

```
               type:  numeric (byte)

              range:  [0,1]                        units:  1
      unique values:  2                        missing .:  0/600

         tabulation:  Freq.  Value
                        329  0
                        271  1
```

---

fingerseite2 **(unlabeled)**

```
               type:  numeric (byte)

              range:  [1,2]                        units:  1
      unique values:  2                        missing .:  538/600

         tabulation:  Freq.  Value
                         24  1
                         38  2
                        538  .
```

```
-------------------------------------------------------------------------------
lokalisation2                                                       (unlabeled)
-------------------------------------------------------------------------------

                  type:  numeric (byte)

                 range:  [0,15]                       units:  1
         unique values:  13                       missing .:  0/600

                  mean:   .506667
              std. dev:   1.86791

           percentiles:        10%       25%       50%       75%       90%
                                 0         0         0         0         1

-------------------------------------------------------------------------------
mcp2                                                                (unlabeled)
-------------------------------------------------------------------------------

                  type:  numeric (byte)

                 range:  [0,1]                        units:  1
         unique values:  2                        missing .:  0/600

            tabulation:  Freq.  Value
                           591  0
                             9  1

31 . codebook pip2 dip2 gg2 mg2 eg2 eintrittspforte generlisierterinfekt

-------------------------------------------------------------------------------
pip2                                                                (unlabeled)
-------------------------------------------------------------------------------

                  type:  numeric (byte)

                 range:  [0,0]                        units:  1
         unique values:  1                        missing .:  3/600

            tabulation:  Freq.  Value
                           597  0
                             3  .

-------------------------------------------------------------------------------
dip2                                                                (unlabeled)
-------------------------------------------------------------------------------

                  type:  numeric (byte)

                 range:  [0,1]                        units:  1
         unique values:  2                        missing .:  0/600

            tabulation:  Freq.  Value
                           597  0
                             3  1

-------------------------------------------------------------------------------
gg2                                                                 (unlabeled)
-------------------------------------------------------------------------------

                  type:  numeric (byte)

                 range:  [0,1]                        units:  1
         unique values:  2                        missing .:  0/600

            tabulation:  Freq.  Value
                           580  0
                            20  1
```

```
---------------------------------------------------------------------------
mg2                                                            (unlabeled)
---------------------------------------------------------------------------

                  type:  numeric (byte)

                 range:  [0,1]                        units:  1
         unique values:  2                        missing .:  0/600

            tabulation:  Freq.  Value
                           588  0
                            12  1

---------------------------------------------------------------------------
eg2                                                            (unlabeled)
---------------------------------------------------------------------------

                  type:  numeric (byte)

                 range:  [0,1]                        units:  1
         unique values:  2                        missing .:  0/600

            tabulation:  Freq.  Value
                           592  0
                             8  1

---------------------------------------------------------------------------
eintrittspforte                                                (unlabeled)
---------------------------------------------------------------------------

                  type:  numeric (byte)

                 range:  [0,6]                        units:  1
         unique values:  7                        missing .:  0/600

            tabulation:  Freq.  Value
                           166  0
                           252  1
                            71  2
                            57  3
                            26  4
                             4  5
                            24  6

---------------------------------------------------------------------------
generlisierterinfekt                                           (unlabeled)
---------------------------------------------------------------------------

                  type:  numeric (byte)

                 range:  [0,1]                        units:  1
         unique values:  2                        missing .:  0/600

            tabulation:  Freq.  Value
                           590  0
                            10  1

32 . centile krea, c (25,50,75)

                                                       -- Binom. Interp. --
    Variable |       Obs  Percentile    Centile        [95% Conf. Interval]
-------------+-------------------------------------------------------------
        krea |       397          25         .8        .7708269         .82
             |                    50        .92              .9         .93
             |                    75      1.065            1.03        1.11
```

```
33 . centile crp, c (25,50,75)

                                                          -- Binom. Interp. --
    Variable |       Obs  Percentile    Centile        [95% Conf. Interval]
-------------+-------------------------------------------------------------
         crp |       423         25         5.2        3.808357         6.1
             |                   50        16.7            13.9        18.9
             |                   75        44.1        34.90652     55.0415

34 . centile glucose, c (25,50,75)

                                                          -- Binom. Interp. --
    Variable |       Obs  Percentile    Centile        [95% Conf. Interval]
-------------+-------------------------------------------------------------
     glucose |       396         25          93              90          94
             |                   50         101             100    103.9907
             |                   75      118.75             116         125

35 . centile leukos, c (25,50,75)

                                                          -- Binom. Interp. --
    Variable |       Obs  Percentile    Centile        [95% Conf. Interval]
-------------+-------------------------------------------------------------
      leukos |       440         25         7.3               7         7.6
             |                   50         9.2             8.9         9.6
             |                   75        11.5        10.94483    11.90567

36 . centile thrombos, c ( 25,50,75)

                                                          -- Binom. Interp. --
    Variable |       Obs  Percentile    Centile        [95% Conf. Interval]
-------------+-------------------------------------------------------------
    thrombos |       443         25         206             198         216
             |                   50         251             240         260
             |                   75         301        293.9202         312

37 . drop punktionhandgelenk fremdkoerper beugesehne eitersehne eitersubkutan fieb
> er

38 . drop karpaltunnel trauma biss anamnese anamnesezusammengefasst druckschmerz

39 . drop pus hautgeschlossen lyse fingerseite1 lokalisation1 mcp1 pip1 dip1 gg1 m
> g1 eg1

40 . drop fingerseite2 lokalisation2 mcp2 pip2 dip2 gg2 mg2 eg2 eintrittspforte kr
> ea glucose

41 . drop leukos thrombos generlisierterinfekt

42 .
43 . ** Infektionen - Keime
44 . tab keim1, m

      keim1 |      Freq.     Percent        Cum.
------------+-----------------------------------
          0 |        259       43.17       43.17
          1 |        166       27.67       70.83
          2 |         25        4.17       75.00
          3 |         66       11.00       86.00
          4 |         42        7.00       93.00
          5 |         42        7.00      100.00
------------+-----------------------------------
      Total |        600      100.00
```

```
45 . codebook last1 keimnachweis1

-------------------------------------------------------------------------------
last1                                                               (unlabeled)
-------------------------------------------------------------------------------

                  type:  numeric (byte)

                 range:  [0,7]                        units:  1
         unique values:  6                        missing .:  0/600

            tabulation:  Freq.  Value
                           259  0
                           110  1
                            80  2
                            84  4
                            37  6
                            30  7

-------------------------------------------------------------------------------
keimnachweis1                                                       (unlabeled)
-------------------------------------------------------------------------------

                  type:  numeric (byte)

                 range:  [0,5]                        units:  1
         unique values:  6                        missing .:  0/600

            tabulation:  Freq.  Value
                           122  0
                           273  1
                            93  2
                           110  3
                             1  4
                             1  5

46 . tab keim2, m
```

keim2	Freq.	Percent	Cum.
0	471	78.50	78.50
1	10	1.67	80.17
2	51	8.50	88.67
3	36	6.00	94.67
4	4	0.67	95.33
5	28	4.67	100.00
Total	600	100.00	

```
47 . codebook last2 keimnachweis2

-------------------------------------------------------------------------------
last2                                                               (unlabeled)
-------------------------------------------------------------------------------

                  type:  numeric (byte)

                 range:  [0,7]                        units:  1
         unique values:  6                        missing .:  0/600

            tabulation:  Freq.  Value
                           469  0
                            54  1
                            43  2
                            19  4
                            11  6
                             4  7

-------------------------------------------------------------------------------
keimnachweis2                                                       (unlabeled)
-------------------------------------------------------------------------------
```

```
                 type:  numeric (byte)

                range:  [0,3]                        units:  1
        unique values:  4                        missing .:  1/600

           tabulation:  Freq.  Value
                          467  0
                           83  1
                           32  2
                           17  3
                            1  .
```

48 . drop keim1 last1 keimnachweis1 keim2 last2 keimnachweis2

49 . tab aureus, m

aureus	Freq.	Percent	Cum.
0	422	70.33	70.33
1	178	29.67	100.00
Total	600	100.00	

50 . tab gramnegative, m

gramnegativ e	Freq.	Percent	Cum.
0	522	87.00	87.00
1	78	13.00	100.00
Total	600	100.00	

51 . tab strept, m

strept	Freq.	Percent	Cum.
0	505	84.17	84.17
1	95	15.83	100.00
Total	600	100.00	

52 . codebook keim3 last3 keimnachweis3 keim4 last4 keimnachweis4 keim5 last5

keim3 **(unlabeled)**

```
                 type:  numeric (byte)

                range:  [0,5]                        units:  1
        unique values:  6                        missing .:  0/600

           tabulation:  Freq.  Value
                          538  0
                            3  1
                           23  2
                           11  3
                            1  4
                           24  5
```

last3 **(unlabeled)**

```
                 type:  numeric (byte)

                range:  [0,6]                        units:  1
        unique values:  5                        missing .:  5/600
```

```
           tabulation:  Freq.  Value
                          533  0
                           21  1
                           21  2
                           17  4
                            3  6
                            5  .
```

keimnachweis3 **(unlabeled)**

```
                 type:  numeric (byte)

                range:  [0,3]                    units:  1
        unique values:  4                    missing .:  6/600

           tabulation:  Freq.  Value
                          532  0
                           36  1
                           16  2
                           10  3
                            6  .
```

keim4 **(unlabeled)**

```
                 type:  numeric (byte)

                range:  [0,5]                    units:  1
        unique values:  4                    missing .:  0/600

           tabulation:  Freq.  Value
                          561  0
                           22  2
                            6  3
                           11  5
```

last4 **(unlabeled)**

```
                 type:  numeric (byte)

                range:  [0,7]                    units:  1
        unique values:  6                    missing .:  4/600

           tabulation:  Freq.  Value
                          557  0
                           14  1
                           11  2
                            8  4
                            5  6
                            1  7
                            4  .
```

keimnachweis4 **(unlabeled)**

```
                 type:  numeric (byte)

                range:  [0,3]                    units:  1
        unique values:  4                    missing .:  4/600
```

```
            tabulation:  Freq.  Value
                           557  0
                            21  1
                            11  2
                             7  3
                             4  .

-------------------------------------------------------------------------------
keim5                                                               (unlabeled)
-------------------------------------------------------------------------------

                  type:  numeric (byte)

                 range:  [0,5]                        units:  1
         unique values:  5                        missing .:  0/600

            tabulation:  Freq.  Value
                           584  0
                             1  1
                             7  2
                             2  3
                             6  5

-------------------------------------------------------------------------------
last5                                                               (unlabeled)
-------------------------------------------------------------------------------

                  type:  numeric (byte)

                 range:  [0,6]                        units:  1
         unique values:  4                        missing .:  7/600

            tabulation:  Freq.  Value
                           578  0
                             6  1
                             6  2
                             3  6
                             7  .

53 . codebook keimnachweis5 keim6 last6 keimnachweis6 keim7 last7 keimnachweis7

-------------------------------------------------------------------------------
keimnachweis5                                                       (unlabeled)
-------------------------------------------------------------------------------

                  type:  numeric (byte)

                 range:  [0,3]                        units:  1
         unique values:  4                        missing .:  5/600

            tabulation:  Freq.  Value
                           579  0
                             5  1
                             6  2
                             5  3
                             5  .

-------------------------------------------------------------------------------
keim6                                                               (unlabeled)
-------------------------------------------------------------------------------

                  type:  numeric (byte)

                 range:  [0,5]                        units:  1
         unique values:  3                        missing .:  0/600

            tabulation:  Freq.  Value
                           592  0
                             5  2
                             3  5
```

last6 **(unlabeled)**

type: numeric (**byte**)

range: [**0**,**7**] units: **1**
unique values: **5** missing .: **5**/**600**

tabulation:

Freq.	Value
587	0
1	2
4	4
1	6
2	7
5	.

keimnachweis6 **(unlabeled)**

type: numeric (**byte**)

range: [**0**,**2**] units: **1**
unique values: **3** missing .: **10**/**600**

tabulation:

Freq.	Value
582	0
4	1
4	2
10	.

keim7 **(unlabeled)**

type: numeric (**byte**)

range: [**0**,**5**] units: **1**
unique values: **4** missing .: **0**/**600**

tabulation:

Freq.	Value
594	0
1	2
1	3
4	5

last7 **(unlabeled)**

type: numeric (**byte**)

range: [**0**,**4**] units: **1**
unique values: **4** missing .: **6**/**600**

tabulation:

Freq.	Value
588	0
4	1
1	2
1	4
6	.

keimnachweis7 **(unlabeled)**

type: numeric (**byte**)

range: [**0**,**2**] units: **1**
unique values: **3** missing .: **6**/**600**

```
            tabulation:  Freq.  Value
                           588  0
                             3  1
                             3  2
                             6  .

54 . codebook keim8 last8 keimnachweis8 keim9 last9 keimnachweis9 keim10 last10

---------------------------------------------------------------------------
keim8                                                          (unlabeled)
---------------------------------------------------------------------------

                  type:  numeric (byte)

                 range:  [0,3]                        units:  1
         unique values:  2                        missing .:  0/600

            tabulation:  Freq.  Value
                           598  0
                             2  3

---------------------------------------------------------------------------
last8                                                          (unlabeled)
---------------------------------------------------------------------------

                  type:  numeric (byte)

                 range:  [0,1]                        units:  1
         unique values:  2                        missing .:  4/600

            tabulation:  Freq.  Value
                           594  0
                             2  1
                             4  .

---------------------------------------------------------------------------
keimnachweis8                                                  (unlabeled)
---------------------------------------------------------------------------

                  type:  numeric (byte)

                 range:  [0,2]                        units:  1
         unique values:  3                        missing .:  3/600

            tabulation:  Freq.  Value
                           595  0
                             1  1
                             1  2
                             3  .

---------------------------------------------------------------------------
keim9                                                          (unlabeled)
---------------------------------------------------------------------------

                  type:  numeric (byte)

                 range:  [0,5]                        units:  1
         unique values:  2                        missing .:  0/600

            tabulation:  Freq.  Value
                           599  0
                             1  5

---------------------------------------------------------------------------
last9                                                          (unlabeled)
---------------------------------------------------------------------------

                  type:  numeric (byte)

                 range:  [0,1]                        units:  1
         unique values:  2                        missing .:  3/600
```

```
          tabulation:  Freq.  Value
                         596  0
                           1  1
                           3  .

-------------------------------------------------------------------------------
keimnachweis9                                                       (unlabeled)
-------------------------------------------------------------------------------

                type:  numeric (byte)

               range:  [0,1]                        units:  1
       unique values:  2                        missing .:  4/600

          tabulation:  Freq.  Value
                         595  0
                           1  1
                           4  .

-------------------------------------------------------------------------------
keim10                                                              (unlabeled)
-------------------------------------------------------------------------------

                type:  numeric (byte)

               range:  [0,2]                        units:  1
       unique values:  2                        missing .:  0/600

          tabulation:  Freq.  Value
                         599  0
                           1  2

-------------------------------------------------------------------------------
last10                                                              (unlabeled)
-------------------------------------------------------------------------------

                type:  numeric (byte)

               range:  [0,1]                        units:  1
       unique values:  2                        missing .:  4/600

          tabulation:  Freq.  Value
                         595  0
                           1  1
                           4  .

55 . codebook keimnachweis10 histo

-------------------------------------------------------------------------------
keimnachweis10                                                      (unlabeled)
-------------------------------------------------------------------------------

                type:  numeric (byte)

               range:  [0,1]                        units:  1
       unique values:  2                        missing .:  3/600

          tabulation:  Freq.  Value
                         596  0
                           1  1
                           3  .

-------------------------------------------------------------------------------
histo                                                               (unlabeled)
-------------------------------------------------------------------------------

                type:  numeric (byte)

               range:  [1,2]                        units:  1
       unique values:  2                        missing .:  570/600
```

```
tabulation:  Freq.  Value
                19  1
                11  2
               570  .
```

56 . tab rezidivkeim1, m

rezidivkeim 1	Freq.	Percent	Cum.
0	587	97.83	97.83
1	4	0.67	98.50
2	4	0.67	99.17
5	5	0.83	100.00
Total	600	100.00	

57 . tab rezidivkeim2, m

rezidivkeim 2	Freq.	Percent	Cum.
0	596	99.33	99.33
2	2	0.33	99.67
3	1	0.17	99.83
5	1	0.17	100.00
Total	600	100.00	

58 . tab rezidivkeim3, m

rezidivkeim 3	Freq.	Percent	Cum.
0	597	99.50	99.50
2	3	0.50	100.00
Total	600	100.00	

59 . tab rezidivkeim4, m

rezidivkeim 4	Freq.	Percent	Cum.
0	599	99.83	99.83
2	1	0.17	100.00
Total	600	100.00	

60 . tab keimuebersicht, m

keimuebersicht	Freq.	Percent	Cum.
	536	89.33	89.33
0	2	0.33	89.67
1	1	0.17	89.83
15	1	0.17	90.00
2	1	0.17	90.17
4	1	0.17	90.33
7	1	0.17	90.50
8	1	0.17	90.67
9	1	0.17	90.83
Actinomyces canis	1	0.17	91.00
Actinomyces europaeus	1	0.17	91.17
Anaerococcus hydrogenalis	1	0.17	91.33
Anaerococcus vaginalis	1	0.17	91.50
Bacillus weihenstephanensis	1	0.17	91.67
Bacteroides pyogenes	1	0.17	91.83
Bergeyella zoohlecum	1	0.17	92.00
Clostridium perfringens	1	0.17	92.17
Corynebacterium simulans	1	0.17	92.33
Eggerthia catenaformis	1	0.17	92.50

Eikanella corrodens	1	0.17	92.67
Fusobacterium canifelinum	1	0.17	92.83
Gemella morbillorum	1	0.17	93.00
Granulicatella adiacens	1	0.17	93.17
Haemophilus parainfluenzae	1	0.17	93.33
Klebsiella oxytoca	1	0.17	93.50
Kocuria kristinae	1	0.17	93.67
Lactobacillus gasseri	1	0.17	93.83
Leptotrichia spp.	1	0.17	94.00
Neisseria animaloris	1	0.17	94.17
Neisseria subflava	1	0.17	94.33
Neisseria zooswgmatis	1	0.17	94.50
Parvimonas micra	1	0.17	94.67
Pasteurella stomatis	1	0.17	94.83
Peptoniphilus harei	1	0.17	95.00
Peptostreptococcus canis	1	0.17	95.17
Poephyromonas gingivalis	1	0.17	95.33
Porphyromonas somorae	1	0.17	95.50
Prevotalla melaninogenica	1	0.17	95.67
Prevotella bivia	1	0.17	95.83
Prevotella disiens	1	0.17	96.00
Prevotella melaninofenica	1	0.17	96.17
Prevotella nigrescens	1	0.17	96.33
Prevotella pallens	1	0.17	96.50
Prevotella spp.	1	0.17	96.67
Raoultella ornithinolytica	1	0.17	96.83
Serratia liquefaciens	1	0.17	97.00
Serratia marcescens	1	0.17	97.17
Solobacterium moorei	1	0.17	97.33
Staph. Epidermidis	1	0.17	97.50
Staph. Warneri	1	0.17	97.67
Strep. Canis	1	0.17	97.83
Strep. Constellatus	1	0.17	98.00
Strep. Epidermidis	1	0.17	98.17
Strep. Intermedius	1	0.17	98.33
Strep. Salivarius	1	0.17	98.50
Strep. Sanguis	1	0.17	98.67
Strep. Spp.	1	0.17	98.83
Strep. anginosus	1	0.17	99.00
Strep. gordonii	1	0.17	99.17
Trueperella bernardiae	1	0.17	99.33
Veillonella parvula	1	0.17	99.50
anaerobe gramneg. Kokken	1	0.17	99.67
gramneg. St□bchen	1	0.17	99.83
physiologische Haut-&Schleimhautflora	1	0.17	100.00
Total	600	100.00	

```
61 . drop keim3 last3 keimnachweis3 keim4 last4 keimnachweis4 keim5 last5 keimnach
   > weis5

62 . drop keim6 last6 keimnachweis6 keim7 last7 keimnachweis7 keim8 last8 keimnach
   > weis8

63 . drop keim9 last9 keimnachweis9 keim10 last10 keimnachweis10 histo rezidivkeim
   > 1

64 . drop rezidivkeim2 rezidivkeim3 rezidivkeim4 keimuebersicht

65 . count
  600
```

```
66 .
67 . *** BEHANDLUNG
68 . ** Behandlung - Chirurgie
69 . centile anzahlopgesamt, c (25,50,75)

                                                         -- Binom. Interp. --
    Variable |        Obs  Percentile    Centile        [95% Conf. Interval]
-------------+-------------------------------------------------------------
anzahlopge~t |        600          25          1               1           1
             |                     50          1               1           1
             |                     75          2               1           2

70 . centile anzahlophause, c (25,50,75)

                                                         -- Binom. Interp. --
    Variable |        Obs  Percentile    Centile        [95% Conf. Interval]
-------------+-------------------------------------------------------------
             |

71 . codebook operateur assistent anaesthesie abtraeger sehnenresektion knochenres
> ektion

-------------------------------------------------------------------------------
operateur                                                           (unlabeled)
-------------------------------------------------------------------------------

                  type:  numeric (byte)

                 range:  [1,4]                        units:  1
         unique values:  4                        missing .:  34/600

            tabulation:  Freq.  Value
                            73  1
                           122  2
                           369  3
                             2  4
                            34  .

-------------------------------------------------------------------------------
assistent                                                           (unlabeled)
-------------------------------------------------------------------------------

                  type:  numeric (byte)

                 range:  [1,3]                        units:  1
         unique values:  3                        missing .:  433/600

            tabulation:  Freq.  Value
                            22  1
                            59  2
                            86  3
                           433  .

-------------------------------------------------------------------------------
anaesthesie                                                         (unlabeled)
-------------------------------------------------------------------------------

                  type:  numeric (byte)

                 range:  [1,5]                        units:  1
         unique values:  5                        missing .:  12/600

            tabulation:  Freq.  Value
                           414  1
                           124  2
                            46  3
                             2  4
                             2  5
                            12  .
```

```
-------------------------------------------------------------------------------
abtraeger                                                           (unlabeled)
-------------------------------------------------------------------------------

                  type:  numeric (byte)

                 range:  [0,1]                        units:  1
         unique values:  2                        missing .:  0/600

            tabulation:  Freq.  Value
                           578  0
                            22  1

-------------------------------------------------------------------------------
sehnenresektion                                                     (unlabeled)
-------------------------------------------------------------------------------

                  type:  numeric (byte)

                 range:  [1,2]                        units:  1
         unique values:  2                        missing .:  0/600

            tabulation:  Freq.  Value
                            56  1
                           544  2

-------------------------------------------------------------------------------
knochenresektion                                                    (unlabeled)
-------------------------------------------------------------------------------

                  type:  numeric (byte)

                 range:  [1,3]                        units:  1
         unique values:  3                        missing .:  551/600

            tabulation:  Freq.  Value
                            29  1
                             1  2
                            19  3
                           551  .

72 . codebook opbevornotfall abvorher analgesia antiinflammatorydrugs lappen

-------------------------------------------------------------------------------
opbevornotfall                                                      (unlabeled)
-------------------------------------------------------------------------------

                  type:  numeric (byte)

                 range:  [1,2]                        units:  1
         unique values:  2                        missing .:  0/600

            tabulation:  Freq.  Value
                            76  1
                           524  2

-------------------------------------------------------------------------------
abvorher                                                            (unlabeled)
-------------------------------------------------------------------------------

                  type:  numeric (byte)

                 range:  [1,2]                        units:  1
         unique values:  2                        missing .:  0/600

            tabulation:  Freq.  Value
                           113  1
                           487  2
```

```
-------------------------------------------------------------------------------
analgesia                                                           (unlabeled)
-------------------------------------------------------------------------------

                  type:  numeric (byte)

                 range:  [0,2]                        units:  1
         unique values:  3                        missing .:  0/600

            tabulation:  Freq.  Value
                            85  0
                           513  1
                             2  2

-------------------------------------------------------------------------------
antiinflammatorydrugs                                               (unlabeled)
-------------------------------------------------------------------------------

                  type:  numeric (byte)

                 range:  [0,12]                       units:  1
         unique values:  13                       missing .:  0/600

                  mean:      2.01
              std. dev:    1.9697

           percentiles:        10%       25%       50%       75%       90%
                                 0         1         1         4         5

-------------------------------------------------------------------------------
lappen                                                              (unlabeled)
-------------------------------------------------------------------------------

                  type:  numeric (byte)

                 range:  [0,4]                        units:  1
         unique values:  5                        missing .:  0/600

            tabulation:  Freq.  Value
                           574  0
                            12  1
                             1  2
                             9  3
                             4  4

73 . codebook gelenkversteifung functionaloutcome ruhigstellung

-------------------------------------------------------------------------------
gelenkversteifung                                                   (unlabeled)
-------------------------------------------------------------------------------

                  type:  numeric (byte)

                 range:  [1,2]                        units:  1
         unique values:  2                        missing .:  0/600

            tabulation:  Freq.  Value
                             9  1
                           591  2

-------------------------------------------------------------------------------
functionaloutcome                                                   (unlabeled)
-------------------------------------------------------------------------------

                  type:  numeric (byte)

                 range:  [0,5]                        units:  1
         unique values:  6                        missing .:  0/600
```

tabulation:

Freq.	Value
10	0
5	1
580	2
2	3
1	4
2	5

---

ruhigstellung **(unlabeled)**

---

type: numeric (**byte**)

range: [**1,3**] units: **1**
unique values: **3** missing .: **47**/**600**

tabulation:

Freq.	Value
333	1
219	2
1	3
47	.

74 . centile opdauer, c (25,50,75)

Variable	Obs	Percentile	Centile	— Binom. Interp. — [95% Conf.	Interval]
opdauer	558	25	.0118056	.0105461	.0125
		50	.01875	.0173611	.0201389
		75	.0298611	.0277778	.0326389

75 . centile zeitintervalltraumanotfall, c (25,50,75)

Variable	Obs	Percentile	Centile	— Binom. Interp. — [95% Conf.	Interval]
zei~anotfall	449	25	1	1	2
		50	4	3	4
		75	9	7	12

76 . centile zeitintervallbeschwerdenotfall, c (25,50,75)

Variable	Obs	Percentile	Centile	— Binom. Interp. — [95% Conf.	Interval]

77 . centile zeitintervallnotfallop, c (25,50,75)

Variable	Obs	Percentile	Centile	— Binom. Interp. — [95% Conf.	Interval]
zeitinterv~p	585	25	0	0	0
		50	0	0	0
		75	1	1	1

78 . centile dauerruhigstellung, c (25,50,75)

Variable	Obs	Percentile	Centile	— Binom. Interp. — [95% Conf.	Interval]
dauerruhig~g	369	25	0	0	0
		50	0	0	0
		75	3	2	3

```
79 . drop operateur assistent anaesthesie anzahlophause opbevornotfall analgesia

80 . drop zeitintervalltraumanotfall zeitintervallbeschwerdenotfal zeitintervallno
   > tfallop

81 . drop antiinflammatorydrugs functionaloutcome dauerruhigstellung

82 .
83 . ** Behandlung - Antibiotika
84 . tab ab1, m
```

ab1	Freq.	Percent	Cum.
1	33	5.50	5.50
2	102	17.00	22.50
3	353	58.83	81.33
4	1	0.17	81.50
5	3	0.50	82.00
6	33	5.50	87.50
8	1	0.17	87.67
9	38	6.33	94.00
10	2	0.33	94.33
11	1	0.17	94.50
14	2	0.33	94.83
15	5	0.83	95.67
16	2	0.33	96.00
17	3	0.50	96.50
18	1	0.17	96.67
19	1	0.17	96.83
20	3	0.50	97.33
21	1	0.17	97.50
22	1	0.17	97.67
.	14	2.33	100.00
Total	600	100.00	

```
85 . tab ab2, m
```

ab2	Freq.	Percent	Cum.
1	9	1.50	1.50
2	38	6.33	7.83
3	28	4.67	12.50
4	17	2.83	15.33
5	9	1.50	16.83
6	7	1.17	18.00
7	1	0.17	18.17
8	6	1.00	19.17
9	5	0.83	20.00
12	1	0.17	20.17
13	1	0.17	20.33
14	1	0.17	20.50
15	1	0.17	20.67
17	1	0.17	20.83
18	1	0.17	21.00
19	1	0.17	21.17
20	1	0.17	21.33
21	1	0.17	21.50
22	1	0.17	21.67
23	1	0.17	21.83
24	1	0.17	22.00
43726	1	0.17	22.17
.	467	77.83	100.00
Total	600	100.00	

```
86 . tab ab3, m

        ab3 |      Freq.     Percent        Cum.
------------+-----------------------------------
          1 |          1        0.17        0.17
          3 |          4        0.67        0.83
          4 |          6        1.00        1.83
          5 |          1        0.17        2.00
          6 |          2        0.33        2.33
          8 |          3        0.50        2.83
         13 |          1        0.17        3.00
         16 |          2        0.33        3.33
         17 |          1        0.17        3.50
          . |        579       96.50      100.00
------------+-----------------------------------
      Total |        600      100.00

87 . tab ab4, m

        ab4 |      Freq.     Percent        Cum.
------------+-----------------------------------
          7 |          1        0.17        0.17
          . |        599       99.83      100.00
------------+-----------------------------------
      Total |        600      100.00

88 . drop ab1 ab2 ab3 ab4

89 . centile dauerabtotal, c (25,50,75)

                                                       -- Binom. Interp. --
    Variable |       Obs  Percentile    Centile        [95% Conf. Interval]
-------------+-------------------------------------------------------------
dauerabtotal |       454         25          7                7           7
             |                   50          8                8           9
             |                   75         11               11          12

90 .
91 . ** Kategorisation Dauer der postoperativen Antibiotika
92 . gen dauerabgrp=dauerabtotal
   (146 missing values generated)

93 . replace dauerabgrp=1 if (dauerabtotal<=7)
   (188 real changes made)

94 . replace dauerabgrp=2 if (dauerabtotal>7 & dauerabtotal<=11)
   (157 real changes made)

95 . replace dauerabgrp=3 if (dauerabtotal>=11)
   (282 real changes made)

96 . tab dauerabgrp, m

 dauerabgrp |      Freq.     Percent        Cum.
------------+-----------------------------------
          1 |        190       31.67       31.67
          2 |        128       21.33       53.00
          3 |        282       47.00      100.00
------------+-----------------------------------
      Total |        600      100.00
```

```
 97 .
 98 . *** OUTCOMES
 99 . codebook sensibility wundheilungsstoerung nekrosepostop nachamputation

-------------------------------------------------------------------------------
sensibility                                                         (unlabeled)
-------------------------------------------------------------------------------

                  type:  numeric (byte)

                 range:  [0,3]                        units:  1
         unique values:  4                        missing .:  0/600

            tabulation:  Freq.  Value
                             8  0
                             5  1
                           586  2
                             1  3

-------------------------------------------------------------------------------
wundheilungsstoerung                                                (unlabeled)
-------------------------------------------------------------------------------

                  type:  numeric (byte)

                 range:  [0,1]                        units:  1
         unique values:  2                        missing .:  0/600

            tabulation:  Freq.  Value
                           527  0
                            73  1

-------------------------------------------------------------------------------
nekrosepostop                                                       (unlabeled)
-------------------------------------------------------------------------------

                  type:  numeric (byte)

                 range:  [1,2]                        units:  1
         unique values:  2                        missing .:  0/600

            tabulation:  Freq.  Value
                            18  1
                           582  2

-------------------------------------------------------------------------------
nachamputation                                                      (unlabeled)
-------------------------------------------------------------------------------

                  type:  numeric (byte)

                 range:  [1,2]                        units:  1
         unique values:  2                        missing .:  0/600

            tabulation:  Freq.  Value
                            15  1
                           585  2

100 . centile dauerhosp1, c (25,50,75)

                                                       -- Binom. Interp. --
    Variable |       Obs  Percentile    Centile        [95% Conf. Interval]
-------------+-------------------------------------------------------------
  dauerhosp1 |       600          25          3               3           3
             |                    50          4               4           5
             |                    75          7               7           8
```

101 . centile dauerhosp2, c (25,50,75)

Variable	Obs	Percentile	Centile	— Binom. Interp. — [95% Conf.	Interval]
dauerhosp2	36	25	3	2	4.837993
		50	5	4	6.418499
		75	9	6	14.39617

102 . centile dauerhosp3, c (25,50,75)

Variable	Obs	Percentile	Centile	— Binom. Interp. — [95% Conf.	Interval]
dauerhosp3	3	25	3	3	10.46667*
		50	3	3	11*
		75	11	3	11*

* Lower (upper) confidence limit held at minimum (maximum) of sample

103 . tab death, m

death	Freq.	Percent	Cum.
1	2	0.33	0.33
2	598	99.67	100.00
Total	600	100.00	

104 . drop dauerhosp1 dauerhosp2 dauerhosp3 death sensibility nachamputation

105 . drop nekrosepostop

106 . tab softtissuecoverage, m

softtissuec overage	Freq.	Percent	Cum.
0	552	92.00	92.00
1	48	8.00	100.00
Total	600	100.00	

107 . tab amputation, m

amputation	Freq.	Percent	Cum.
0	542	90.33	90.33
1	58	9.67	100.00
Total	600	100.00	

108 . tab failure1, m

failure1	Freq.	Percent	Cum.
0	493	82.17	82.17
1	107	17.83	100.00
Total	600	100.00	

```
109 . tab failure2, m
```

failure2	Freq.	Percent	Cum.
0	458	76.33	76.33
1	142	23.67	100.00
Total	600	100.00	

```
110 . tab samerecurrence, m
```

samerecurrence	Freq.	Percent	Cum.
0	597	99.50	99.50
1	3	0.50	100.00
Total	600	100.00	

```
111 .
112 . ** VERGLEICH Was macht Lymphangitis?
113 . tab sex lymphangitis, chi col
```

Key
frequency<br>*column percentage*

sex	lymphangitis 1	2	Total
1	53<br>61.63	309<br>60.12	362<br>60.33
2	33<br>38.37	205<br>39.88	238<br>39.67
Total	86<br>100.00	514<br>100.00	600<br>100.00

Pearson chi2(**1**) = **0.0703** Pr = **0.791**

```
114 . gen agelymphangitis=age if lymphangitis==1
    (514 missing values generated)

115 . gen agenichts=age if lymphangitis==0
    (600 missing values generated)

116 . centile agelymphangitis agenichts
```

Variable	Obs	Percentile	Centile	— Binom. Interp. — [95% Conf.	Interval]
agelymphan~s	86	50	50	42.38581	54.61419
agenichts	0				

```
117 . ranksum age, by (lymphangitis)
```

Two-sample Wilcoxon rank-sum (Mann-Whitney) test

lymphangitis	obs	rank sum	expected
1	86	22822.5	25843
2	514	157477.5	154457
combined	600	180300	180300

```
    unadjusted variance  2213883.67
    adjustment for ties    -753.40
                         ----------
    adjusted variance    2213130.27

    Ho: age(lympha~s==1) = age(lympha~s==2)
                 z =  -2.030
        Prob > |z| =   0.0423

118 . gen bmilymphangitis=bmi if lymphangitis==1
    (526 missing values generated)

119 . gen bminichts=bmi if lymphangitis==0
    (600 missing values generated)

120 . centile bmilymphangitis bminichts

                                                           -- Binom. Interp. --
        Variable |       Obs  Percentile    Centile        [95% Conf. Interval]
    -------------+-------------------------------------------------------------
    bmilymphan~s |        74         50       25.35             24.2    26.29213
       bminichts |       0

121 . ranksum bmi, by (lymphangitis)

    Two-sample Wilcoxon rank-sum (Mann-Whitney) test

    lymphangitis |      obs    rank sum    expected
    -------------+---------------------------------
               1 |       74     16793.5       18796
               2 |      433    111984.5      109982
    -------------+---------------------------------
        combined |      507      128778      128778

    unadjusted variance  1356444.67
    adjustment for ties    -148.19
                         ----------
    adjusted variance    1356296.47

    Ho: bmi(lympha~s==1) = bmi(lympha~s==2)
                 z =  -1.719
        Prob > |z| =   0.0855

122 . gen crplymphangitis=crp if lymphangitis==1
    (539 missing values generated)

123 . gen crpnichts=crp if lymphangitis==0
    (600 missing values generated)

124 . centile crplymphangitis crpnichts

                                                           -- Binom. Interp. --
        Variable |       Obs  Percentile    Centile        [95% Conf. Interval]
    -------------+-------------------------------------------------------------
    crplymphan~s |        61         50        16.7         12.35077    23.77957
       crpnichts |       0

125 . ranksum crp, by (lymphangitis)

    Two-sample Wilcoxon rank-sum (Mann-Whitney) test

    lymphangitis |      obs    rank sum    expected
    -------------+---------------------------------
               1 |       61     13066.5       12932
               2 |      362     76609.5       76744
    -------------+---------------------------------
        combined |      423       89676       89676
```

```
unadjusted variance   780230.67
adjustment for ties      -20.35
                     ----------
adjusted variance     780210.32

Ho: crp(lympha~s==1) = crp(lympha~s==2)
             z =   0.152
    Prob > |z| =   0.8790
```

126 . tab aureus lymphangitis, chi col

Key
frequency   *column percentage*

aureus	lymphangitis 1	2	Total
0	58<br>67.44	364<br>70.82	422<br>70.33
1	28<br>32.56	150<br>29.18	178<br>29.67
Total	86<br>100.00	514<br>100.00	600<br>100.00

Pearson chi2(1) =   0.4022   Pr = 0.526

127 . tab gramnegative lymphangitis, chi col

Key
frequency   *column percentage*

gramnegati ve	lymphangitis 1	2	Total
0	75<br>87.21	447<br>86.96	522<br>87.00
1	11<br>12.79	67<br>13.04	78<br>13.00
Total	86<br>100.00	514<br>100.00	600<br>100.00

Pearson chi2(1) =   0.0039   Pr = 0.950

128 . tab strept lymphangitis, chi col

Key
frequency   *column percentage*

strept	lymphangitis 1	2	Total
0	**71**<br>**82.56**	**434**<br>**84.44**	**505**<br>**84.17**
1	**15**<br>**17.44**	**80**<br>**15.56**	**95**<br>**15.83**
Total	**86**<br>**100.00**	**514**<br>**100.00**	**600**<br>**100.00**

Pearson chi2(**1**) = **0.1949** Pr = **0.659**

129 . tab diabetes lymphangitis, chi col

Key
frequency<br>*column percentage*

diabetes	lymphangitis 1	2	Total
0	**77**<br>**89.53**	**452**<br>**87.94**	**529**<br>**88.17**
1	**9**<br>**10.47**	**62**<br>**12.06**	**71**<br>**11.83**
Total	**86**<br>**100.00**	**514**<br>**100.00**	**600**<br>**100.00**

Pearson chi2(**1**) = **0.1801** Pr = **0.671**

130 . tab eitergelenk lymphangitis, chi col

Key
frequency<br>*column percentage*

eitergelen k	lymphangitis 1	2	Total
1	**3**<br>**3.49**	**38**<br>**7.39**	**41**<br>**6.83**
2	**83**<br>**96.51**	**476**<br>**92.61**	**559**<br>**93.17**
Total	**86**<br>**100.00**	**514**<br>**100.00**	**600**<br>**100.00**

Pearson chi2(**1**) = **1.7643** Pr = **0.184**

```
131 . gen ablymphangitis=dauerabtotal if lymphangitis==1
    (528 missing values generated)

132 . gen abnichts=dauerabtotal if lymphangitis==0
    (600 missing values generated)

133 . centile ablymphangitis abnichts
```

Variable	Obs	Percentile	Centile	— Binom. Interp. — [95% Conf.	Interval]
ablymphang~s	72	50	8	8	9
abnichts	0				

```
134 . ranksum dauerabtotal, by (lymphangitis)

    Two-sample Wilcoxon rank-sum (Mann-Whitney) test
```

lymphangitis	obs	rank sum	expected
1	72	16076.5	16380
2	382	87208.5	86905
combined	454	103285	103285

```
    unadjusted variance  1042860.00
    adjustment for ties   -14676.97
                         ----------
    adjusted variance    1028183.03

    Ho: dauera~l(lympha~s==1) = dauera~l(lympha~s==2)
                 z =  -0.299
        Prob > |z| =   0.7647

135 . tab dauerabgrp lymphangitis, chi col
```

Key
frequency<br>*column percentage*

dauerabgrp	lymphangitis 1	2	Total
1	27<br>31.40	163<br>31.71	190<br>31.67
2	25<br>29.07	103<br>20.04	128<br>21.33
3	34<br>39.53	248<br>48.25	282<br>47.00
Total	86<br>100.00	514<br>100.00	600<br>100.00

```
          Pearson chi2(2) =   4.0091   Pr = 0.135
```

136 . tab softtissuecoverage lymphangitis, chi col

Key
frequency<br>*column percentage*

softtissue coverage	lymphangitis 1	2	Total
0	**83**<br>**96.51**	**469**<br>**91.25**	**552**<br>**92.00**
1	**3**<br>**3.49**	**45**<br>**8.75**	**48**<br>**8.00**
Total	**86**<br>**100.00**	**514**<br>**100.00**	**600**<br>**100.00**

Pearson chi2(**1**) = **2.7764** Pr = **0.096**

137 . tab amputation lymphangitis, chi col

Key
frequency<br>*column percentage*

amputation	lymphangitis 1	2	Total
0	**82**<br>**95.35**	**460**<br>**89.49**	**542**<br>**90.33**
1	**4**<br>**4.65**	**54**<br>**10.51**	**58**<br>**9.67**
Total	**86**<br>**100.00**	**514**<br>**100.00**	**600**<br>**100.00**

Pearson chi2(**1**) = **2.8920** Pr = **0.089**

138 . tab failure1 lymphangitis, chi col

Key
frequency<br>*column percentage*

failure1	lymphangitis 1	2	Total
0	**79**<br>**91.86**	**414**<br>**80.54**	**493**<br>**82.17**
1	**7**<br>**8.14**	**100**<br>**19.46**	**107**<br>**17.83**
Total	**86**<br>**100.00**	**514**<br>**100.00**	**600**<br>**100.00**

Pearson chi2(**1**) = **6.4379** Pr = **0.011**

```
139 . tab failure2 lymphangitis, chi col
```

Key
frequency   *column percentage*

failure2	lymphangitis 1	2	Total
0	**75**<br>**87.21**	**383**<br>**74.51**	**458**<br>**76.33**
1	**11**<br>**12.79**	**131**<br>**25.49**	**142**<br>**23.67**
Total	**86**<br>**100.00**	**514**<br>**100.00**	**600**<br>**100.00**

Pearson chi2(**1**) = **6.5731** Pr = **0.010**

```
140 . tab samerecurrence lymphangitis, chi col
```

Key
frequency   *column percentage*

samerecurr ence	lymphangitis 1	2	Total
0	**86**<br>**100.00**	**511**<br>**99.42**	**597**<br>**99.50**
1	**0**<br>**0.00**	**3**<br>**0.58**	**3**<br>**0.50**
Total	**86**<br>**100.00**	**514**<br>**100.00**	**600**<br>**100.00**

Pearson chi2(**1**) = **0.5045** Pr = **0.478**

```
141 . drop crplymphangitis crpnichts agelymphangitis agenichts bmilymphangitis bmin
    > ichts

142 . drop ablymphangitis abnichts lymphangitis

143 .
144 . *** OUTCOME SOFT TISSUE COVERAGE
145 . ***Gruppenvergleich
146 . tab sex softtissuecoverage, chi col
```

Key
frequency   *column percentage*

sex	softtissuecoverage 0	1	Total
1	332<br>60.14	30<br>62.50	362<br>60.33
2	220<br>39.86	18<br>37.50	238<br>39.67
Total	552<br>100.00	48<br>100.00	600<br>100.00

Pearson chi2(1) = 0.1023   Pr = 0.749

147 . gen agesofttissuecoverage=age if softtissuecoverage==1
(552 missing values generated)

148 . gen agenichts=age if softtissuecoverage==0
(48 missing values generated)

149 . centile agesofttissuecoverage agenichts

Variable	Obs	Percentile	Centile	— Binom. Interp. — [95% Conf.	Interval]
agesofttis~e	48	50	58.5	55	63.31379
agenichts	552	50	53	52	55

150 . ranksum age, by (softtissuecoverage)

Two-sample Wilcoxon rank-sum (Mann-Whitney) test

softtissue~e	obs	rank sum	expected
0	552	163246	165876
1	48	17054	14424
combined	600	180300	180300

unadjusted variance 1327008.00
adjustment for ties -451.59

adjusted variance 1326556.41

Ho: age(softti~e==0) = age(softti~e==1)
z = -2.283
Prob > |z| = 0.0224

151 . gen bmisofttissuecoverage=bmi if softtissuecoverage==1
(553 missing values generated)

152 . gen bminichts=bmi if softtissuecoverage==0
(140 missing values generated)

153 . centile bmisofttissuecoverage bminichts

Variable	Obs	Percentile	Centile	— Binom. Interp. — [95% Conf.	Interval]
bmisofttis~e	47	50	26.2	25.05141	27.97429
bminichts	460	50	26	25.6	26.6

```
154 . ranksum bmi, by (softtissuecoverage)

    Two-sample Wilcoxon rank-sum (Mann-Whitney) test

    softtissue~e |      obs    rank sum    expected
    -------------+---------------------------------
               0 |      460    116206.5      116840
               1 |       47     12571.5       11938
    -------------+---------------------------------
        combined |      507      128778      128778

    unadjusted variance   915246.67
    adjustment for ties      -99.99
                          ----------
    adjusted variance     915146.67

    Ho: bmi(softti~e==0) = bmi(softti~e==1)
                 z =  -0.662
        Prob > |z| =   0.5078

155 . gen crpsofttissuecoverage=crp if softtissuecoverage==1
    (558 missing values generated)

156 . gen crpnichts=crp if softtissuecoverage==0
    (219 missing values generated)

157 . centile crpsofttissuecoverage crpnichts

                                                          -- Binom. Interp. --
        Variable |       Obs  Percentile    Centile        [95% Conf. Interval]
    -------------+-------------------------------------------------------------
    crpsofttis~e |        42         50        32.4             19.7    76.69676
       crpnichts |       381         50        14.9             13.1        17.9

158 . ranksum crp, by (softtissuecoverage)

    Two-sample Wilcoxon rank-sum (Mann-Whitney) test

    softtissue~e |      obs    rank sum    expected
    -------------+---------------------------------
               0 |      381     78011.5       80772
               1 |       42     11664.5        8904
    -------------+---------------------------------
        combined |      423       89676       89676

    unadjusted variance   565404.00
    adjustment for ties      -14.75
                          ----------
    adjusted variance     565389.25

    Ho: crp(softti~e==0) = crp(softti~e==1)
                 z =  -3.671
        Prob > |z| =   0.0002

159 . gen opdauersofttissuecoverage=opdauer if softtissuecoverage==1
    (552 missing values generated)

160 . gen opdauernichts=opdauer if softtissuecoverage==0
    (90 missing values generated)
```

```
161 . centile opdauersofttissuecoverage opdauernichts

                                                       -- Binom. Interp. --
    Variable |       Obs  Percentile    Centile        [95% Conf. Interval]
-------------+-------------------------------------------------------------
opdauersof~e |        48          50   .0239583        .0208333    .0401556
opdauernic~s |       510          50   .0180556        .0166667      .01875

162 . ranksum opdauer, by (softtissuecoverage)

Two-sample Wilcoxon rank-sum (Mann-Whitney) test

softtissue~e |      obs    rank sum    expected
-------------+---------------------------------
           0 |      510    137773.5      142545
           1 |       48     18187.5       13416
-------------+---------------------------------
    combined |      558      155961      155961

unadjusted variance  1140360.00
adjustment for ties     -544.02
                     ----------
adjusted variance    1139815.98

Ho: opdauer(softti~e==0) = opdauer(softti~e==1)
             z =  -4.469
    Prob > |z| =   0.0000

163 .
164 . ** Kategorisierung Op-Dauer
165 . centile opdauer, c (25,50,75)

                                                       -- Binom. Interp. --
    Variable |       Obs  Percentile    Centile        [95% Conf. Interval]
-------------+-------------------------------------------------------------
     opdauer |       558          25   .0118056        .0105461       .0125
             |                    50     .01875        .0173611    .0201389
             |                    75   .0298611        .0277778    .0326389

166 . gen opdauergrp=opdauer
    (42 missing values generated)

167 . replace opdauergrp=1 if (opdauer<=0.012)
    (149 real changes made)

168 . replace opdauergrp=2 if (opdauer>0.012 & opdauer<=0.019)
    (141 real changes made)

169 . replace opdauergrp=3 if (opdauer>=0.019)
    (310 real changes made)

170 . tab opdauergrp, m

 opdauergrp |      Freq.     Percent        Cum.
------------+-----------------------------------
          1 |        149       24.83       24.83
          2 |        141       23.50       48.33
          3 |        310       51.67      100.00
------------+-----------------------------------
      Total |        600      100.00
```

```
171 .
172 . gen anzahlopgesamtsofttissuecoverage=anzahlopgesamt if softtissuecoverage==1
    (552 missing values generated)

173 . gen anzahlopgesamtnichts=anzahlopgesamt if softtissuecoverage==0
    (48 missing values generated)

174 . centile anzahlopgesamtsofttissuecoverage anzahlopgesamtnichts
```

Variable	Obs	Percentile	Centile	— Binom. Interp. — [95% Conf.	Interval]
anzahlopge~e	**48**	**50**	**3**	**3**	**4**
anzahlopge~s	**552**	**50**	**1**	**1**	**1**

```
175 . ranksum anzahlopgesamt, by (softtissuecoverage)
```

Two-sample Wilcoxon rank-sum (Mann-Whitney) test

softtissue~e	obs	rank sum	expected
0	**552**	**154192**	**165876**
1	**48**	**26108**	**14424**
combined	**600**	**180300**	**180300**

```
unadjusted variance  1327008.00
adjustment for ties  -488368.72
                     ----------
adjusted variance     838639.28

Ho: anzahl~t(softti~e==0) = anzahl~t(softti~e==1)
             z = -12.759
    Prob > |z| =   0.0000
```

```
176 . tab abtraeger softtissuecoverage, chi col
```

Key
frequency<br>*column percentage*

abtraeger	softtissuecoverage 0	1	Total
0	**532**<br>**96.38**	**46**<br>**95.83**	**578**<br>**96.33**
1	**20**<br>**3.62**	**2**<br>**4.17**	**22**<br>**3.67**
Total	**552**<br>**100.00**	**48**<br>**100.00**	**600**<br>**100.00**

Pearson chi2(**1**) =   **0.0369**   Pr = **0.848**

```
177 . tab sehnenresektion softtissuecoverage, chi col
```

Key
frequency<br>*column percentage*

sehnenresektion	softtissuecoverage 0	1	Total
1	46<br>8.33	10<br>20.83	56<br>9.33
2	506<br>91.67	38<br>79.17	544<br>90.67
Total	552<br>100.00	48<br>100.00	600<br>100.00

Pearson chi2(**1**) = **8.1539** Pr = **0.004**

178 . tab knochenresektion softtissuecoverage, chi col

Key
frequency<br>*column percentage*

knochenresektion	softtissuecoverage 0	1	Total
1	27<br>60.00	2<br>50.00	29<br>59.18
2	1<br>2.22	0<br>0.00	1<br>2.04
3	17<br>37.78	2<br>50.00	19<br>38.78
Total	45<br>100.00	4<br>100.00	49<br>100.00

Pearson chi2(**2**) = **0.2925** Pr = **0.864**

179 . tab wundheilungsstoerung softtissuecoverage, chi col

Key
frequency<br>*column percentage*

wundheilungsstoerung	softtissuecoverage 0	1	Total
0	520<br>94.20	7<br>14.58	527<br>87.83
1	32<br>5.80	41<br>85.42	73<br>12.17
Total	552<br>100.00	48<br>100.00	600<br>100.00

Pearson chi2(**1**) = **261.9616** Pr = **0.000**

180 . tab aureus softtissuecoverage, chi col

Key
frequency
column percentage

aureus	softtissuecoverage 0	1	Total
0	**391**<br>**70.83**	**31**<br>**64.58**	**422**<br>**70.33**
1	**161**<br>**29.17**	**17**<br>**35.42**	**178**<br>**29.67**
Total	**552**<br>**100.00**	**48**<br>**100.00**	**600**<br>**100.00**

Pearson chi2(**1**) = **0.8267** Pr = **0.363**

181 . tab gramnegative softtissuecoverage, chi col

Key
frequency
column percentage

gramnegati ve	softtissuecoverage 0	1	Total
0	**482**<br>**87.32**	**40**<br>**83.33**	**522**<br>**87.00**
1	**70**<br>**12.68**	**8**<br>**16.67**	**78**<br>**13.00**
Total	**552**<br>**100.00**	**48**<br>**100.00**	**600**<br>**100.00**

Pearson chi2(**1**) = **0.6202** Pr = **0.431**

182 . tab strept softtissuecoverage, chi col

Key
frequency
column percentage

strept	softtissuecoverage 0	1	Total
0	**470**<br>**85.14**	**35**<br>**72.92**	**505**<br>**84.17**
1	**82**<br>**14.86**	**13**<br>**27.08**	**95**<br>**15.83**
Total	**552**<br>**100.00**	**48**<br>**100.00**	**600**<br>**100.00**

Pearson chi2(**1**) = **4.9550** Pr = **0.026**

183 . tab diabetes softtissuecoverage, chi col

Key
frequency   *column percentage*

diabetes	softtissuecoverage 0	1	Total
0	**496** **89.86**	**33** **68.75**	**529** **88.17**
1	**56** **10.14**	**15** **31.25**	**71** **11.83**
Total	**552** **100.00**	**48** **100.00**	**600** **100.00**

Pearson chi2(**1**) = **18.8535** Pr = **0.000**

184 . tab eitergelenk softtissuecoverage, chi col

Key
frequency   *column percentage*

eitergelen k	softtissuecoverage 0	1	Total
1	**33** **5.98**	**8** **16.67**	**41** **6.83**
2	**519** **94.02**	**40** **83.33**	**559** **93.17**
Total	**552** **100.00**	**48** **100.00**	**600** **100.00**

Pearson chi2(**1**) = **7.9243** Pr = **0.005**

185 . tab gelenkversteifung softtissuecoverage, chi col

Key
frequency   *column percentage*

gelenkvers teifung	softtissuecoverage 0	1	Total
1	**8** **1.45**	**1** **2.08**	**9** **1.50**
2	**544** **98.55**	**47** **97.92**	**591** **98.50**
Total	**552** **100.00**	**48** **100.00**	**600** **100.00**

Pearson chi2(**1**) = **0.1202** Pr = **0.729**

```
186 . gen absofttissuecoverage=dauerabtotal if softtissuecoverage==1
    (577 missing values generated)

187 . gen abnichts=dauerabtotal if softtissuecoverage==0
    (169 missing values generated)

188 . centile absofttissuecoverage abnichts
```

Variable	Obs	Percentile	Centile	— Binom. Interp. — [95% Conf.	Interval]
absofttiss~e	23	50	20	11.52388	31.42837
abnichts	431	50	8	8	9

```
189 . ranksum dauerabtotal, by (softtissuecoverage)

    Two-sample Wilcoxon rank-sum (Mann-Whitney) test
```

softtissue~e	obs	rank sum	expected
0	431	95380	98052.5
1	23	7905	5232.5
combined	454	103285	103285

```
    unadjusted variance    375867.92
    adjustment for ties     -5289.88
                           ----------
    adjusted variance      370578.04

    Ho: dauera~l(softti~e==0) = dauera~l(softti~e==1)
                 z =  -4.390
        Prob > |z| =   0.0000

190 . tab dauerabgrp softtissuecoverage, chi col
```

Key
frequency
column percentage

dauerabgrp	softtissuecoverage 0	1	Total
1	186<br>33.70	4<br>8.33	190<br>31.67
2	127<br>23.01	1<br>2.08	128<br>21.33
3	239<br>43.30	43<br>89.58	282<br>47.00
Total	552<br>100.00	48<br>100.00	600<br>100.00

```
          Pearson chi2(2) =  38.1625   Pr = 0.000
```

```
191 . tab ruhigstellung softtissuecoverage, chi col

  +-------------------+
  | Key               |
  |-------------------|
  |     frequency     |
  | column percentage |
  +-------------------+

ruhigstell |  softtissuecoverage
       ung |         0          1 |     Total
-----------+----------------------+----------
         1 |       308         25 |       333 
           |     60.63      55.56 |     60.22 
-----------+----------------------+----------
         2 |       200         19 |       219 
           |     39.37      42.22 |     39.60 
-----------+----------------------+----------
         3 |         0          1 |         1 
           |      0.00       2.22 |      0.18 
-----------+----------------------+----------
     Total |       508         45 |       553 
           |    100.00     100.00 |    100.00 

          Pearson chi2(2) =  11.5506   Pr = 0.003

192 . tab samerecurrence softtissuecoverage, chi col

  +-------------------+
  | Key               |
  |-------------------|
  |     frequency     |
  | column percentage |
  +-------------------+

samerecurr |  softtissuecoverage
      ence |         0          1 |     Total
-----------+----------------------+----------
         0 |       550         47 |       597 
           |     99.64      97.92 |     99.50 
-----------+----------------------+----------
         1 |         2          1 |         3 
           |      0.36       2.08 |      0.50 
-----------+----------------------+----------
     Total |       552         48 |       600 
           |    100.00     100.00 |    100.00 

          Pearson chi2(1) =   2.6291   Pr = 0.105

193 . drop crpsofttissuecoverage crpnichts agesofttissuecoverage agenichts

194 . drop anzahlopgesamtsofttissuecoverage anzahlopgesamtnichts opdauersofttissuec
    > overage

195 . drop absofttissuecoverage abnichts bmisofttissuecoverage bminichts opdauernic
    > hts

196 . 
197 . ** UNIVARIATE COX Regression SOFTISSUECOVERAGE *
198 . stset coxend, fail (softtissuecoverage) origin (dateop) id (id)

                id:  id
     failure event:  softtissuecoverage != 0 & softtissuecoverage < .
obs. time interval:  (coxend[_n-1], coxend]
 exit on or before:  failure
    t for analysis:  (time-origin)
            origin:  time dateop
```

```
------------------------------------------------------------------------------
        600  total observations
          5  ignored because never entered
------------------------------------------------------------------------------
        595  observations remaining, representing
        595  subjects
         48  failures in single-failure-per-subject data
    644,772  total analysis time at risk and under observation
                                                at risk from t =         0
                                     earliest observed entry t =         0
                                          last observed exit t =     1,427

199 . xi: stcox sex

         failure _d:  softtissuecoverage
   analysis time _t:  (coxend-origin)
             origin:  time dateop
                 id:  id

Iteration 0:   log likelihood = -262.86273
Iteration 1:   log likelihood = -262.70288
Iteration 2:   log likelihood = -262.70284
Refining estimates:
Iteration 0:   log likelihood = -262.70284

Cox regression -- Breslow method for ties

No. of subjects =          595                  Number of obs    =         595
No. of failures =           48
Time at risk    =       644772
                                                LR chi2(1)       =        0.32
Log likelihood  =   -262.70284                  Prob > chi2      =      0.5717

------------------------------------------------------------------------------
          _t | Haz. Ratio   Std. Err.      z    P>|z|     [95% Conf. Interval]
-------------+----------------------------------------------------------------
         sex |   .8456609   .2523059    -0.56   0.574     .4712348    1.517593
------------------------------------------------------------------------------

200 . xi: stcox age

         failure _d:  softtissuecoverage
   analysis time _t:  (coxend-origin)
             origin:  time dateop
                 id:  id

Iteration 0:   log likelihood = -262.86273
Iteration 1:   log likelihood = -259.84794
Iteration 2:   log likelihood =  -259.8441
Refining estimates:
Iteration 0:   log likelihood =  -259.8441

Cox regression -- Breslow method for ties

No. of subjects =          595                  Number of obs    =         595
No. of failures =           48
Time at risk    =       644772
                                                LR chi2(1)       =        6.04
Log likelihood  =    -259.8441                  Prob > chi2      =      0.0140

------------------------------------------------------------------------------
          _t | Haz. Ratio   Std. Err.      z    P>|z|     [95% Conf. Interval]
-------------+----------------------------------------------------------------
         age |   1.019391   .0081104     2.41   0.016     1.003618    1.035411
------------------------------------------------------------------------------
```

```
201 . xi: stcox bmi

         failure _d:  softtissuecoverage
   analysis time _t:  (coxend-origin)
             origin:  time dateop
                 id:  id

Iteration 0:   log likelihood = -250.66148
Iteration 1:   log likelihood = -250.21462
Iteration 2:   log likelihood = -250.21217
Iteration 3:   log likelihood = -250.21217
Refining estimates:
Iteration 0:   log likelihood = -250.21217

Cox regression -- Breslow method for ties

No. of subjects =          507                Number of obs    =         507
No. of failures =           47
Time at risk    =       547711
                                              LR chi2(1)       =        0.90
Log likelihood  =   -250.21217                Prob > chi2      =      0.3432
```

_t	Haz. Ratio	Std. Err.	z	P>|z|	[95% Conf.	Interval]
bmi	1.026807	.0279724	0.97	0.332	.97342	1.083122

```
202 . xi: stcox diabetes

         failure _d:  softtissuecoverage
   analysis time _t:  (coxend-origin)
             origin:  time dateop
                 id:  id

Iteration 0:   log likelihood = -262.86273
Iteration 1:   log likelihood = -260.01278
Iteration 2:   log likelihood = -254.34871
Iteration 3:   log likelihood = -254.34456
Iteration 4:   log likelihood = -254.34456
Refining estimates:
Iteration 0:   log likelihood = -254.34456

Cox regression -- Breslow method for ties

No. of subjects =          595                Number of obs    =         595
No. of failures =           48
Time at risk    =       644772
                                              LR chi2(1)       =       17.04
Log likelihood  =   -254.34456                Prob > chi2      =      0.0000
```

_t	Haz. Ratio	Std. Err.	z	P>|z|	[95% Conf.	Interval]
diabetes	4.229595	1.320626	4.62	0.000	2.293634	7.799618

```
203 . xi: stcox dauerabgrp

         failure _d:  softtissuecoverage
   analysis time _t:  (coxend-origin)
             origin:  time dateop
                 id:  id

Iteration 0:   log likelihood = -262.86273
Iteration 1:   log likelihood = -245.77618
Iteration 2:   log likelihood = -244.36855
Iteration 3:   log likelihood = -244.30519
Iteration 4:   log likelihood = -244.30496
Refining estimates:
Iteration 0:   log likelihood = -244.30496
```

```
Cox regression -- Breslow method for ties

No. of subjects =          595                Number of obs    =        595
No. of failures =           48
Time at risk    =       644772
                                               LR chi2(1)       =      37.12
Log likelihood  =   -244.30496                Prob > chi2      =     0.0000

------------------------------------------------------------------------------
          _t | Haz. Ratio   Std. Err.      z    P>|z|     [95% Conf. Interval]
-------------+----------------------------------------------------------------
  dauerabgrp |   3.999098   1.204484     4.60   0.000     2.216101     7.21663
------------------------------------------------------------------------------
```

204 . xi: stcox dauerabtotal

```
         failure _d:  softtissuecoverage
   analysis time _t:  (coxend-origin)
             origin:  time dateop
                 id:  id

Iteration 0:   log likelihood = -120.32699
Iteration 1:   log likelihood = -111.93889
Iteration 2:   log likelihood = -109.00864
Iteration 3:   log likelihood = -109.00133
Iteration 4:   log likelihood = -109.00133
Refining estimates:
Iteration 0:   log likelihood = -109.00133

Cox regression -- Breslow method for ties

No. of subjects =          449                Number of obs    =        449
No. of failures =           23
Time at risk    =       489102
                                               LR chi2(1)       =      22.65
Log likelihood  =   -109.00133                Prob > chi2      =     0.0000

------------------------------------------------------------------------------
          _t | Haz. Ratio   Std. Err.      z    P>|z|     [95% Conf. Interval]
-------------+----------------------------------------------------------------
dauerabtotal |   1.058609   .0102349     5.89   0.000     1.038737     1.07886
------------------------------------------------------------------------------
```

205 . xi: stcox anzahlopgesamt

```
         failure _d:  softtissuecoverage
   analysis time _t:  (coxend-origin)
             origin:  time dateop
                 id:  id

Iteration 0:   log likelihood = -262.86273
Iteration 1:   log likelihood = -262.03827
Iteration 2:   log likelihood = -235.60788
Iteration 3:   log likelihood = -232.35333
Iteration 4:   log likelihood = -221.53233
Iteration 5:   log likelihood = -221.50634
Iteration 6:   log likelihood = -221.50634
Refining estimates:
Iteration 0:   log likelihood = -221.50634

Cox regression -- Breslow method for ties

No. of subjects =          595                Number of obs    =        595
No. of failures =           48
Time at risk    =       644772
                                               LR chi2(1)       =      82.71
Log likelihood  =   -221.50634                Prob > chi2      =     0.0000
```

_t	Haz. Ratio	Std. Err.	z	P>|z|	[95% Conf.	Interval]
anzahlopge~t	1.773435	.0879681	11.55	0.000	1.609136	1.954508

```
206 . xi: stcox abtraeger

         failure _d:  softtissuecoverage
   analysis time _t:  (coxend-origin)
             origin:  time dateop
                 id:  id

Iteration 0:   log likelihood = -262.86273
Iteration 1:   log likelihood = -262.85664
Iteration 2:   log likelihood = -262.85664
Refining estimates:
Iteration 0:   log likelihood = -262.85664

Cox regression -- Breslow method for ties

No. of subjects =          595                  Number of obs    =         595
No. of failures =           48
Time at risk    =       644772
                                                LR chi2(1)       =        0.01
Log likelihood  =   -262.85664                  Prob > chi2      =      0.9121
```

_t	Haz. Ratio	Std. Err.	z	P>|z|	[95% Conf.	Interval]
abtraeger	1.084202	.7842051	0.11	0.911	.2626848	4.474924

```
207 . xi: stcox opdauer

         failure _d:  softtissuecoverage
   analysis time _t:  (coxend-origin)
             origin:  time dateop
                 id:  id

Iteration 0:   log likelihood = -258.38305
Iteration 1:   log likelihood = -250.42696
Iteration 2:   log likelihood = -250.38901
Iteration 3:   log likelihood = -250.38894
Refining estimates:
Iteration 0:   log likelihood = -250.38894

Cox regression -- Breslow method for ties

No. of subjects =          558                  Number of obs    =         558
No. of failures =           48
Time at risk    =       601858
                                                LR chi2(1)       =       15.99
Log likelihood  =   -250.38894                  Prob > chi2      =      0.0001
```

_t	Haz. Ratio	Std. Err.	z	P>|z|	[95% Conf.	Interval]
opdauer	7.11e+07	2.52e+08	5.10	0.000	68683.05	7.37e+10

```
208 . xi: stcox opdauergrp

         failure _d:  softtissuecoverage
   analysis time _t:  (coxend-origin)
             origin:  time dateop
                 id:  id

    Iteration 0:   log likelihood = -262.86273
    Iteration 1:   log likelihood = -257.33303
    Iteration 2:   log likelihood = -257.10102
    Iteration 3:   log likelihood = -257.09973
    Iteration 4:   log likelihood = -257.09973
    Refining estimates:
    Iteration 0:   log likelihood = -257.09973

    Cox regression -- Breslow method for ties

    No. of subjects =          595                 Number of obs    =         595
    No. of failures =           48
    Time at risk    =       644772
                                                   LR chi2(1)       =       11.53
    Log likelihood  =   -257.09973                 Prob > chi2      =      0.0007
```

_t	Haz. Ratio	Std. Err.	z	P>|z|	[95% Conf.	Interval]
opdauergrp	2.04897	.488702	3.01	0.003	1.283851	3.270067

```
209 . xi: stcox ruhigstellung

         failure _d:  softtissuecoverage
   analysis time _t:  (coxend-origin)
             origin:  time dateop
                 id:  id

    Iteration 0:   log likelihood = -240.54577
    Iteration 1:   log likelihood = -239.12037
    Iteration 2:   log likelihood = -239.11257
    Iteration 3:   log likelihood = -239.11257
    Refining estimates:
    Iteration 0:   log likelihood = -239.11257

    Cox regression -- Breslow method for ties

    No. of subjects =          552                 Number of obs    =         552
    No. of failures =           45
    Time at risk    =       597322
                                                   LR chi2(1)       =        2.87
    Log likelihood  =   -239.11257                 Prob > chi2      =      0.0904
```

_t	Haz. Ratio	Std. Err.	z	P>|z|	[95% Conf.	Interval]
ruhigstell~g	1.655871	.4857097	1.72	0.086	.9318523	2.942428

```
210 . xi: stcox knochenresektion

         failure _d:  softtissuecoverage
   analysis time _t:  (coxend-origin)
             origin:  time dateop
                 id:  id

    Iteration 0:   log likelihood = -12.162748
    Iteration 1:   log likelihood = -11.917813
    Iteration 2:   log likelihood = -11.916149
    Refining estimates:
    Iteration 0:   log likelihood = -11.916149

    Cox regression -- no ties
```

```
No. of subjects =           49                Number of obs    =          49
No. of failures =            4
Time at risk    =        52345
                                              LR chi2(1)       =        0.49
Log likelihood  =   -11.916149                Prob > chi2      =      0.4825
```

_t	Haz. Ratio	Std. Err.	z	P>|z|	[95% Conf.	Interval]
knochenres~n	1.434693	.7301838	0.71	0.478	.5291049	3.890238

```
211 . xi: stcox eitergelenk

         failure _d:  softtissuecoverage
   analysis time _t:  (coxend-origin)
             origin:  time dateop
                 id:  id

Iteration 0:   log likelihood = -262.86273
Iteration 1:   log likelihood = -261.33413
Iteration 2:   log likelihood = -259.98389
Iteration 3:   log likelihood = -259.95873
Iteration 4:   log likelihood = -259.95871
Refining estimates:
Iteration 0:   log likelihood = -259.95871

Cox regression -- Breslow method for ties

No. of subjects =          595                Number of obs    =         595
No. of failures =           48
Time at risk    =       644772
                                              LR chi2(1)       =        5.81
Log likelihood  =   -259.95871                Prob > chi2      =      0.0160
```

_t	Haz. Ratio	Std. Err.	z	P>|z|	[95% Conf.	Interval]
eitergelenk	.3484667	.1354416	-2.71	0.007	.1626737	.7464576

```
212 . xi: stcox crp

         failure _d:  softtissuecoverage
   analysis time _t:  (coxend-origin)
             origin:  time dateop
                 id:  id

Iteration 0:   log likelihood = -217.33953
Iteration 1:   log likelihood = -214.98857
Iteration 2:   log likelihood = -211.36274
Iteration 3:   log likelihood = -211.29803
Iteration 4:   log likelihood = -211.29796
Refining estimates:
Iteration 0:   log likelihood = -211.29796

Cox regression -- Breslow method for ties

No. of subjects =          420                Number of obs    =         420
No. of failures =           42
Time at risk    =       449309
                                              LR chi2(1)       =       12.08
Log likelihood  =   -211.29796                Prob > chi2      =      0.0005
```

_t	Haz. Ratio	Std. Err.	z	P>|z|	[95% Conf.	Interval]
crp	1.006149	.0015589	3.96	0.000	1.003098	1.009209

```
213 . xi: stcox sehnenresektion

         failure _d:  softtissuecoverage
   analysis time _t:  (coxend-origin)
             origin:  time dateop
                 id:  id

    Iteration 0:   log likelihood = -262.86273
    Iteration 1:   log likelihood = -260.99003
    Iteration 2:   log likelihood = -260.63278
    Iteration 3:   log likelihood = -260.63112
    Iteration 4:   log likelihood = -260.63112
    Refining estimates:
    Iteration 0:   log likelihood = -260.63112

    Cox regression -- Breslow method for ties

    No. of subjects =          595                  Number of obs    =         595
    No. of failures =           48
    Time at risk    =       644772
                                                    LR chi2(1)       =        4.46
    Log likelihood  =   -260.63112                  Prob > chi2      =      0.0346
```

_t	Haz. Ratio	Std. Err.	z	P>|z|	[95% Conf.	Interval]
sehnenrese~n	.4402046	.1575352	-2.29	0.022	.2182914	.8877129

```
214 . xi: stcox gelenkversteifung

         failure _d:  softtissuecoverage
   analysis time _t:  (coxend-origin)
             origin:  time dateop
                 id:  id

    Iteration 0:   log likelihood = -262.86273
    Iteration 1:   log likelihood = -262.82478
    Iteration 2:   log likelihood = -262.82372
    Iteration 3:   log likelihood = -262.82372
    Refining estimates:
    Iteration 0:   log likelihood = -262.82372

    Cox regression -- Breslow method for ties

    No. of subjects =          595                  Number of obs    =         595
    No. of failures =           48
    Time at risk    =       644772
                                                    LR chi2(1)       =        0.08
    Log likelihood  =   -262.82372                  Prob > chi2      =      0.7800
```

_t	Haz. Ratio	Std. Err.	z	P>|z|	[95% Conf.	Interval]
gelenkvers~g	.7437459	.7528176	-0.29	0.770	.1022918	5.407649

```
215 . xi: stcox wundheilungsstoerung

         failure _d:  softtissuecoverage
   analysis time _t:  (coxend-origin)
             origin:  time dateop
                 id:  id

    Iteration 0:   log likelihood = -262.86273
    Iteration 1:   log likelihood = -209.96101
    Iteration 2:   log likelihood = -192.18257
    Iteration 3:   log likelihood = -191.87153
    Iteration 4:   log likelihood = -191.86889
    Refining estimates:
    Iteration 0:   log likelihood = -191.86889
```

```
Cox regression -- Breslow method for ties

No. of subjects =          595                  Number of obs    =        595
No. of failures =           48
Time at risk    =       644772
                                                LR chi2(1)       =     141.99
Log likelihood  =   -191.86889                  Prob > chi2      =     0.0000
```

_t	Haz. Ratio	Std. Err.	z	P>|z|	[95% Conf.	Interval]
wundheilun~g	47.26315	19.35827	9.41	0.000	21.17793	105.478

```
216 . xi: stcox i.lappen
i.lappen          _Ilappen_0-4        (naturally coded; _Ilappen_0 omitted)

         failure _d:  softtissuecoverage
   analysis time _t:  (coxend-origin)
             origin:  time dateop
                 id:  id

Iteration 0:   log likelihood = -262.86273
Iteration 1:   log likelihood = -239.03575
Iteration 2:   log likelihood = -227.07332
Iteration 3:   log likelihood =  -219.1716
Iteration 4:   log likelihood = -218.12245
Iteration 5:   log likelihood = -218.12147
Refining estimates:
Iteration 0:   log likelihood = -218.12147

Cox regression -- Breslow method for ties

No. of subjects =          595                  Number of obs    =        595
No. of failures =           48
Time at risk    =       644772
                                                LR chi2(4)       =      89.48
Log likelihood  =   -218.12147                  Prob > chi2      =     0.0000
```

_t	Haz. Ratio	Std. Err.	z	P>|z|	[95% Conf.	Interval]
_Ilappen_1	17.22959	6.255212	7.84	0.000	8.457574	35.09976
_Ilappen_2	101.2673	107.2664	4.36	0.000	12.7011	807.4146
_Ilappen_3	21.93566	8.916741	7.60	0.000	9.888751	48.65865
_Ilappen_4	27.19072	14.87879	6.04	0.000	9.303412	79.46926

```
217 . xi: stcox samerecurrence

         failure _d:  softtissuecoverage
   analysis time _t:  (coxend-origin)
             origin:  time dateop
                 id:  id

Iteration 0:   log likelihood = -262.86273
Iteration 1:   log likelihood = -262.70344
Iteration 2:   log likelihood = -262.64824
Iteration 3:   log likelihood = -262.64739
Iteration 4:   log likelihood = -262.64739
Refining estimates:
Iteration 0:   log likelihood = -262.64739

Cox regression -- Breslow method for ties

No. of subjects =          595                  Number of obs    =        595
No. of failures =           48
Time at risk    =       644772
                                                LR chi2(1)       =       0.43
Log likelihood  =   -262.64739                  Prob > chi2      =     0.5117
```

_t	Haz. Ratio	Std. Err.	z	P>|z|	[95% Conf.	Interval]
samerecurr~e	2.133639	2.217136	0.73	0.466	.2783613	16.35434

```
218 . xi: stcox amputation

         failure _d:  softtissuecoverage
   analysis time _t:  (coxend-origin)
             origin:  time dateop
                 id:  id

Iteration 0:   log likelihood = -262.86273
Iteration 1:   log likelihood = -260.72441
Iteration 2:   log likelihood = -260.03061
Iteration 3:   log likelihood = -260.02535
Iteration 4:   log likelihood = -260.02535
Refining estimates:
Iteration 0:   log likelihood = -260.02535

Cox regression -- Breslow method for ties

No. of subjects =          595                 Number of obs    =         595
No. of failures =           48
Time at risk    =       644772
                                               LR chi2(1)       =        5.67
Log likelihood  =   -260.02535                 Prob > chi2      =      0.0172
```

_t	Haz. Ratio	Std. Err.	z	P>|z|	[95% Conf.	Interval]
amputation	2.550089	.9123032	2.62	0.009	1.264839	5.141332

```
219 . xi: stcox aureus

         failure _d:  softtissuecoverage
   analysis time _t:  (coxend-origin)
             origin:  time dateop
                 id:  id

Iteration 0:   log likelihood = -262.86273
Iteration 1:   log likelihood = -262.45826
Iteration 2:   log likelihood =  -262.4571
Iteration 3:   log likelihood =  -262.4571
Refining estimates:
Iteration 0:   log likelihood =  -262.4571

Cox regression -- Breslow method for ties

No. of subjects =          595                 Number of obs    =         595
No. of failures =           48
Time at risk    =       644772
                                               LR chi2(1)       =        0.81
Log likelihood  =    -262.4571                 Prob > chi2      =      0.3677
```

_t	Haz. Ratio	Std. Err.	z	P>|z|	[95% Conf.	Interval]
aureus	1.318611	.3990663	0.91	0.361	.7286317	2.386303

```
220 . xi: stcox gramnegative

         failure _d:  softtissuecoverage
   analysis time _t:  (coxend-origin)
             origin:  time dateop
                 id:  id

    Iteration 0:   log likelihood = -262.86273
    Iteration 1:   log likelihood = -262.74469
    Iteration 2:   log likelihood = -262.74408
    Iteration 3:   log likelihood = -262.74408
    Refining estimates:
    Iteration 0:   log likelihood = -262.74408

    Cox regression -- Breslow method for ties

    No. of subjects =          595                Number of obs    =        595
    No. of failures =           48
    Time at risk    =       644772
                                                  LR chi2(1)       =       0.24
    Log likelihood  =   -262.74408                Prob > chi2      =     0.6262
```

_t	Haz. Ratio	Std. Err.	z	P>|z|	[95% Conf.	Interval]
gramnegative	1.213332	.4714266	0.50	0.619	.5665718	2.598391

```
221 . xi: stcox strept

         failure _d:  softtissuecoverage
   analysis time _t:  (coxend-origin)
             origin:  time dateop
                 id:  id

    Iteration 0:   log likelihood = -262.86273
    Iteration 1:   log likelihood = -262.03021
    Iteration 2:   log likelihood = -262.01409
    Iteration 3:   log likelihood = -262.01409
    Refining estimates:
    Iteration 0:   log likelihood = -262.01409

    Cox regression -- Breslow method for ties

    No. of subjects =          595                Number of obs    =        595
    No. of failures =           48
    Time at risk    =       644772
                                                  LR chi2(1)       =       1.70
    Log likelihood  =   -262.01409                Prob > chi2      =     0.1926
```

_t	Haz. Ratio	Std. Err.	z	P>|z|	[95% Conf.	Interval]
strept	1.554428	.5080304	1.35	0.177	.81917	2.949626

```
222 .
223 . ** MULTIVARIATE COX REGRESSION SOFTTISUECOVERAGE**
224 . xi: stcox sex age eitergelenk diabetes bmi wundheilungsstoerung aureus strept

         failure _d:  softtissuecoverage
   analysis time _t:  (coxend-origin)
             origin:  time dateop
                 id:  id
```

```
Iteration 0:   log likelihood = -250.66148
Iteration 1:   log likelihood = -199.40401
Iteration 2:   log likelihood = -187.53823
Iteration 3:   log likelihood = -186.39271
Iteration 4:   log likelihood = -186.37665
Iteration 5:   log likelihood = -186.37665
Refining estimates:
Iteration 0:   log likelihood = -186.37665

Cox regression -- Breslow method for ties

No. of subjects =          507                  Number of obs    =         507
No. of failures =           47
Time at risk    =       547711
                                                LR chi2(8)       =      128.57
Log likelihood  =   -186.37665                  Prob > chi2      =      0.0000
```

_t	Haz. Ratio	Std. Err.	z	P>|z|	[95% Conf.	Interval]
sex	1.044779	.3500962	0.13	0.896	.5417445	2.014905
age	.9930327	.0109504	-0.63	0.526	.9718006	1.014729
eitergelenk	1.196513	.4981781	0.43	0.667	.5290696	2.705966
diabetes	1.145439	.4407935	0.35	0.724	.5387761	2.435205
bmi	.9995096	.0263534	-0.02	0.985	.9491697	1.052519
wundheilun~g	44.14984	19.66969	8.50	0.000	18.43745	105.7201
aureus	.8620788	.2854261	-0.45	0.654	.4505284	1.649574
strept	.7513638	.2826162	-0.76	0.447	.3594854	1.570432

```
225 . predict p
    (option hr assumed; predicted hazard ratio)
    (93 missing values generated)

226 . roctab softtissuecoverage p
```

Obs	ROC Area	Std. Err.	—Asymptotic Normal— [95% Conf.	Interval]
507	0.8973	0.0308	0.83702	0.95761

```
227 . drop p

228 .
229 . *** OUTCOME AMPUTATION
230 . ***Gruppenvergleich
231 . tab sex amputation, chi col
```

Key
frequency<br>*column percentage*

sex	amputation 0	1	Total
1	318<br>58.67	44<br>75.86	362<br>60.33
2	224<br>41.33	14<br>24.14	238<br>39.67
Total	542<br>100.00	58<br>100.00	600<br>100.00

Pearson chi2(1) =   6.4695   Pr = 0.011

```
232 . gen ageamputation=age if amputation==1
    (542 missing values generated)

233 . gen agenichts=age if amputation==0
    (58 missing values generated)

234 . centile ageamputation agenichts
```

Variable	Obs	Percentile	Centile	— Binom. Interp. — [95% Conf.	Interval]
ageamputat~n	58	50	59	53.1056	67.9472
agenichts	542	50	53	52	55

```
235 . ranksum age, by (amputation)

    Two-sample Wilcoxon rank-sum (Mann-Whitney) test
```

amputation	obs	rank sum	expected
0	542	159107	162871
1	58	21193	17429
combined	600	180300	180300

```
    unadjusted variance  1574419.67
    adjustment for ties     -535.79
                         ----------
    adjusted variance    1573883.88

    Ho: age(amputa~n==0) = age(amputa~n==1)
                 z =  -3.000
        Prob > |z| =   0.0027

236 . gen bmiamputation=bmi if amputation==1
    (548 missing values generated)

237 . gen bminichts=bmi if amputation==0
    (145 missing values generated)

238 . centile bmiamputation bminichts
```

Variable	Obs	Percentile	Centile	— Binom. Interp. — [95% Conf.	Interval]
bmiamputat~n	52	50	26.35	24.23986	28.54056
bminichts	455	50	26	25.6	26.6

```
239 . ranksum bmi, by (amputation)

    Two-sample Wilcoxon rank-sum (Mann-Whitney) test
```

amputation	obs	rank sum	expected
0	455	115018	115570
1	52	13760	13208
combined	507	128778	128778

```
    unadjusted variance  1001606.67
    adjustment for ties     -109.43
                         ----------
    adjusted variance    1001497.24

    Ho: bmi(amputa~n==0) = bmi(amputa~n==1)
                 z =  -0.552
        Prob > |z| =   0.5812
```

```
240 . gen crpamputation=crp if amputation==1
    (561 missing values generated)

241 . gen crpnichts=crp if amputation==0
    (216 missing values generated)

242 . centile crpamputation crpnichts

                                                          -- Binom. Interp. --
    Variable |       Obs  Percentile    Centile        [95% Conf. Interval]
-------------+-------------------------------------------------------------
crpamputat~n |        39         50        30.3        19.64498    60.00916
   crpnichts |       384         50       15.85        13.22873    18.04254

243 . ranksum crp, by (amputation)

    Two-sample Wilcoxon rank-sum (Mann-Whitney) test

  amputation |      obs    rank sum    expected
-------------+---------------------------------
           0 |      384     79723.5       81408
           1 |       39      9952.5        8268
-------------+---------------------------------
    combined |      423       89676       89676

    unadjusted variance   529152.00
    adjustment for ties      -13.80
                          ----------
    adjusted variance     529138.20

    Ho: crp(amputa~n==0) = crp(amputa~n==1)
                 z =  -2.316
        Prob > |z| =   0.0206

244 . gen opdaueramputation=opdauer if amputation==1
    (542 missing values generated)

245 . gen opdauernichts=opdauer if amputation==0
    (100 missing values generated)

246 . centile opdaueramputation opdauernichts

                                                          -- Binom. Interp. --
    Variable |       Obs  Percentile    Centile        [95% Conf. Interval]
-------------+-------------------------------------------------------------
opdaueramp~n |        58         50    .0215278        .0180556    .0284356
opdauernic~s |       500         50    .0180556        .0173611    .0194444

247 . ranksum opdauer, by (amputation)

    Two-sample Wilcoxon rank-sum (Mann-Whitney) test

  amputation |      obs    rank sum    expected
-------------+---------------------------------
           0 |      500    136539.5      139750
           1 |       58     19421.5       16211
-------------+---------------------------------
    combined |      558      155961      155961

    unadjusted variance  1350916.67
    adjustment for ties     -644.46
                          ----------
    adjusted variance    1350272.20

    Ho: opdauer(amputa~n==0) = opdauer(amputa~n==1)
                 z =  -2.763
        Prob > |z| =   0.0057
```

```
248 . gen anzahlopgesamtamputation=anzahlopgesamt if amputation==1
    (542 missing values generated)

249 . gen anzahlopgesamtnichts=anzahlopgesamt if amputation==0
    (58 missing values generated)

250 . centile anzahlopgesamtamputation anzahlopgesamtnichts
```

Variable	Obs	Percentile	Centile	— Binom. Interp. — [95% Conf.	Interval]
anzahlopge~n	58	50	2	1	2
anzahlopge~s	542	50	1	1	1

```
251 . ranksum anzahlopgesamt, by (amputation)
```

Two-sample Wilcoxon rank-sum (Mann-Whitney) test

amputation	obs	rank sum	expected
0	542	158251.5	162871
1	58	22048.5	17429
combined	600	180300	180300

```
unadjusted variance  1574419.67
adjustment for ties  -579421.77
                     ----------
adjusted variance     994997.90

Ho: anzahl~t(amputa~n==0) = anzahl~t(amputa~n==1)
             z =  -4.631
    Prob > |z| =   0.0000

252 . tab abtraeger amputation, chi col
```

Key
frequency *column percentage*

abtraeger	amputation 0	1	Total
0	526 97.05	52 89.66	578 96.33
1	16 2.95	6 10.34	22 3.67
Total	542 100.00	58 100.00	600 100.00

```
          Pearson chi2(1) =   8.1067   Pr = 0.004

253 . tab sehnenresektion amputation, chi col
```

Key
frequency *column percentage*

sehnenresektion	amputation 0	1	Total
1	**28**<br>**5.17**	**28**<br>**48.28**	**56**<br>**9.33**
2	**514**<br>**94.83**	**30**<br>**51.72**	**544**<br>**90.67**
Total	**542**<br>**100.00**	**58**<br>**100.00**	**600**<br>**100.00**

Pearson chi2(**1**) = **115.0652**   Pr = **0.000**

254 . tab knochenresektion amputation, chi col

Key
frequency<br>*column percentage*

knochenresektion	amputation 1	Total
1	**29**<br>**59.18**	**29**<br>**59.18**
2	**1**<br>**2.04**	**1**<br>**2.04**
3	**19**<br>**38.78**	**19**<br>**38.78**
Total	**49**<br>**100.00**	**49**<br>**100.00**

255 . tab wundheilungsstoerung amputation, chi col

Key
frequency<br>*column percentage*

wundheilungsstoerung	amputation 0	1	Total
0	**484**<br>**89.30**	**43**<br>**74.14**	**527**<br>**87.83**
1	**58**<br>**10.70**	**15**<br>**25.86**	**73**<br>**12.17**
Total	**542**<br>**100.00**	**58**<br>**100.00**	**600**<br>**100.00**

Pearson chi2(**1**) = **11.2693**   Pr = **0.001**

256 . tab aureus amputation, chi col

Key
frequency<br>*column percentage*

aureus	amputation 0	1	Total
0	**393**<br>**72.51**	**29**<br>**50.00**	**422**<br>**70.33**
1	**149**<br>**27.49**	**29**<br>**50.00**	**178**<br>**29.67**
Total	**542**<br>**100.00**	**58**<br>**100.00**	**600**<br>**100.00**

Pearson chi2(**1**) = **12.7223** Pr = **0.000**

257 . tab gramnegative amputation, chi col

Key
frequency<br>*column percentage*

gramnegati ve	amputation 0	1	Total
0	**475**<br>**87.64**	**47**<br>**81.03**	**522**<br>**87.00**
1	**67**<br>**12.36**	**11**<br>**18.97**	**78**<br>**13.00**
Total	**542**<br>**100.00**	**58**<br>**100.00**	**600**<br>**100.00**

Pearson chi2(**1**) = **2.0203** Pr = **0.155**

258 . tab strept amputation, chi col

Key
frequency<br>*column percentage*

strept	amputation 0	1	Total
0	**458**<br>**84.50**	**47**<br>**81.03**	**505**<br>**84.17**
1	**84**<br>**15.50**	**11**<br>**18.97**	**95**<br>**15.83**
Total	**542**<br>**100.00**	**58**<br>**100.00**	**600**<br>**100.00**

Pearson chi2(**1**) = **0.4727** Pr = **0.492**

259 . tab diabetes amputation, chi col

Key
frequency   *column percentage*

diabetes	amputation 0	1	Total
0	**488** **90.04**	**41** **70.69**	**529** **88.17**
1	**54** **9.96**	**17** **29.31**	**71** **11.83**
Total	**542** **100.00**	**58** **100.00**	**600** **100.00**

Pearson chi2(**1**) = **18.7976** Pr = **0.000**

260 . tab eitergelenk amputation, chi col

Key
frequency   *column percentage*

eitergelen k	amputation 0	1	Total
1	**33** **6.09**	**8** **13.79**	**41** **6.83**
2	**509** **93.91**	**50** **86.21**	**559** **93.17**
Total	**542** **100.00**	**58** **100.00**	**600** **100.00**

Pearson chi2(**1**) = **4.8851** Pr = **0.027**

261 . tab gelenkversteifung softtissuecoverage, chi col

Key
frequency   *column percentage*

gelenkvers teifung	softtissuecoverage 0	1	Total
1	**8** **1.45**	**1** **2.08**	**9** **1.50**
2	**544** **98.55**	**47** **97.92**	**591** **98.50**
Total	**552** **100.00**	**48** **100.00**	**600** **100.00**

Pearson chi2(**1**) = **0.1202** Pr = **0.729**

```
262 . gen abamputation=dauerabtotal if amputation==1
    (563 missing values generated)

263 . gen abnichts=dauerabtotal if amputation==0
    (183 missing values generated)

264 . centile abamputation abnichts
```

Variable	Obs	Percentile	Centile	— Binom. Interp. — [95% Conf.	Interval]
abamputation	37	50	10	8	12
abnichts	417	50	8	8	9

```
265 . ranksum dauerabtotal, by (amputation)
```

Two-sample Wilcoxon rank-sum (Mann-Whitney) test

amputation	obs	rank sum	expected
0	417	93249.5	94867.5
1	37	10035.5	8417.5
combined	454	103285	103285

```
unadjusted variance   585016.25
adjustment for ties    -8233.38
                     ----------
adjusted variance     576782.87

Ho: dauera~l(amputa~n==0) = dauera~l(amputa~n==1)
             z =  -2.130
    Prob > |z| =   0.0331
```

```
266 . tab dauerabgrp amputation, chi col
```

Key
frequency
column percentage

dauerabgrp	amputation 0	1	Total
1	180<br>33.21	10<br>17.24	190<br>31.67
2	118<br>21.77	10<br>17.24	128<br>21.33
3	244<br>45.02	38<br>65.52	282<br>47.00
Total	542<br>100.00	58<br>100.00	600<br>100.00

Pearson chi2(2) =   9.4073   Pr = 0.009

267 . tab ruhigstellung amputation, chi col

Key
frequency
column percentage

ruhigstellung	amputation 0	amputation 1	Total
1	**308**<br>**61.72**	**25**<br>**46.30**	**333**<br>**60.22**
2	**190**<br>**38.08**	**29**<br>**53.70**	**219**<br>**39.60**
3	**1**<br>**0.20**	**0**<br>**0.00**	**1**<br>**0.18**
Total	**499**<br>**100.00**	**54**<br>**100.00**	**553**<br>**100.00**

Pearson chi2(**2**) = **5.0390** Pr = **0.081**

268 . tab samerecurrence amputation, chi col

Key
frequency
column percentage

samerecurrence	amputation 0	amputation 1	Total
0	**540**<br>**99.63**	**57**<br>**98.28**	**597**<br>**99.50**
1	**2**<br>**0.37**	**1**<br>**1.72**	**3**<br>**0.50**
Total	**542**<br>**100.00**	**58**<br>**100.00**	**600**<br>**100.00**

Pearson chi2(**1**) = **1.9340** Pr = **0.164**

269 . drop crpamputation crpnichts ageamputation agenichts

270 . drop anzahlopgesamtamputation anzahlopgesamtnichts opdaueramputation

271 . drop abamputation abnichts bmiamputation bminichts opdauernichts

```
272 .
273 . ** UNIVARIATE COX Regression AMPUTATION *
274 . stset coxend, fail (amputation) origin (dateop) id (id)

                id:  id
     failure event:  amputation != 0 & amputation < .
obs. time interval:  (coxend[_n-1], coxend]
 exit on or before:  failure
    t for analysis:  (time-origin)
            origin:  time dateop
```

```
------------------------------------------------------------------------------
        600  total observations
          5  ignored because never entered
------------------------------------------------------------------------------
        595  observations remaining, representing
        595  subjects
         58  failures in single-failure-per-subject data
    644,772  total analysis time at risk and under observation
                                                at risk from t =         0
                                     earliest observed entry t =         0
                                          last observed exit t =     1,427

275 . xi: stcox sex

         failure _d:  amputation
   analysis time _t:  (coxend-origin)
             origin:  time dateop
                 id:  id

Iteration 0:   log likelihood = -314.28893
Iteration 1:   log likelihood = -310.71229
Iteration 2:   log likelihood = -310.67473
Iteration 3:   log likelihood = -310.67472
Refining estimates:
Iteration 0:   log likelihood = -310.67472

Cox regression -- Breslow method for ties

No. of subjects =          595                  Number of obs    =         595
No. of failures =           58
Time at risk    =       644772
                                                LR chi2(1)       =        7.23
Log likelihood  =   -310.67472                  Prob > chi2      =      0.0072
```

_t	Haz. Ratio	Std. Err.	z	P>|z|	[95% Conf.	Interval]
sex	.4589153	.1409293	-2.54	0.011	.2513826	.8377799

```
276 . xi: stcox age

         failure _d:  amputation
   analysis time _t:  (coxend-origin)
             origin:  time dateop
                 id:  id

Iteration 0:   log likelihood = -314.28893
Iteration 1:   log likelihood =  -309.5853
Iteration 2:   log likelihood = -309.57641
Iteration 3:   log likelihood = -309.57641
Refining estimates:
Iteration 0:   log likelihood = -309.57641

Cox regression -- Breslow method for ties

No. of subjects =          595                  Number of obs    =         595
No. of failures =           58
Time at risk    =       644772
                                                LR chi2(1)       =        9.43
Log likelihood  =   -309.57641                  Prob > chi2      =      0.0021
```

_t	Haz. Ratio	Std. Err.	z	P>|z|	[95% Conf.	Interval]
age	1.022392	.0075391	3.00	0.003	1.007722	1.037276

```
277 . xi: stcox bmi

         failure _d:  amputation
   analysis time _t:  (coxend-origin)
             origin:  time dateop
                 id:  id

Iteration 0:   log likelihood = -274.69307
Iteration 1:   log likelihood = -274.32967
Iteration 2:   log likelihood = -274.32823
Iteration 3:   log likelihood = -274.32823
Refining estimates:
Iteration 0:   log likelihood = -274.32823

Cox regression -- Breslow method for ties

No. of subjects =          507                 Number of obs    =          507
No. of failures =           52
Time at risk    =       547711
                                               LR chi2(1)       =         0.73
Log likelihood  =   -274.32823                 Prob > chi2      =       0.3930
```

_t	Haz. Ratio	Std. Err.	z	P>|z|	[95% Conf.	Interval]
bmi	1.023128	.0268286	0.87	0.383	.971873	1.077085

```
278 . xi: stcox diabetes

         failure _d:  amputation
   analysis time _t:  (coxend-origin)
             origin:  time dateop
                 id:  id

Iteration 0:   log likelihood = -314.28893
Iteration 1:   log likelihood = -310.73593
Iteration 2:   log likelihood = -305.43287
Iteration 3:   log likelihood = -305.41621
Iteration 4:   log likelihood = -305.41621
Refining estimates:
Iteration 0:   log likelihood = -305.41621

Cox regression -- Breslow method for ties

No. of subjects =          595                 Number of obs    =          595
No. of failures =           58
Time at risk    =       644772
                                               LR chi2(1)       =        17.75
Log likelihood  =   -305.41621                 Prob > chi2      =       0.0000
```

_t	Haz. Ratio	Std. Err.	z	P>|z|	[95% Conf.	Interval]
diabetes	3.910291	1.131712	4.71	0.000	2.217457	6.895455

```
279 . xi: stcox dauerabgrp

         failure _d:  amputation
   analysis time _t:  (coxend-origin)
             origin:  time dateop
                 id:  id

Iteration 0:   log likelihood = -314.28893
Iteration 1:   log likelihood = -309.18123
Iteration 2:   log likelihood = -309.13141
Iteration 3:   log likelihood = -309.13139
Refining estimates:
Iteration 0:   log likelihood = -309.13139
```

```
Cox regression -- Breslow method for ties

No. of subjects =          595                 Number of obs    =         595
No. of failures =           58
Time at risk    =       644772
                                               LR chi2(1)       =       10.32
Log likelihood  =   -309.13139                 Prob > chi2      =      0.0013

------------------------------------------------------------------------------
          _t | Haz. Ratio   Std. Err.      z    P>|z|     [95% Conf. Interval]
-------------+----------------------------------------------------------------
  dauerabgrp |   1.693306   .2948793     3.02   0.002      1.20366     2.38214
------------------------------------------------------------------------------

280 . xi: stcox dauerabtotal

         failure _d:  amputation
   analysis time _t:  (coxend-origin)
             origin:  time dateop
                 id:  id

Iteration 0:   log likelihood = -189.29178
Iteration 1:   log likelihood = -183.42294
Iteration 2:   log likelihood = -183.42227
Refining estimates:
Iteration 0:   log likelihood = -183.42227

Cox regression -- Breslow method for ties

No. of subjects =          449                 Number of obs    =         449
No. of failures =           37
Time at risk    =       489102
                                               LR chi2(1)       =       11.74
Log likelihood  =   -183.42227                 Prob > chi2      =      0.0006

------------------------------------------------------------------------------
          _t | Haz. Ratio   Std. Err.      z    P>|z|     [95% Conf. Interval]
-------------+----------------------------------------------------------------
dauerabtotal |   1.041267   .0102301     4.12   0.000     1.021408    1.061512
------------------------------------------------------------------------------

281 . xi: stcox anzahlopgesamt

         failure _d:  amputation
   analysis time _t:  (coxend-origin)
             origin:  time dateop
                 id:  id

Iteration 0:   log likelihood = -314.28893
Iteration 1:   log likelihood = -307.70863
Iteration 2:   log likelihood = -307.67149
Iteration 3:   log likelihood = -307.67143
Refining estimates:
Iteration 0:   log likelihood = -307.67143

Cox regression -- Breslow method for ties

No. of subjects =          595                 Number of obs    =         595
No. of failures =           58
Time at risk    =       644772
                                               LR chi2(1)       =       13.23
Log likelihood  =   -307.67143                 Prob > chi2      =      0.0003

------------------------------------------------------------------------------
          _t | Haz. Ratio   Std. Err.      z    P>|z|     [95% Conf. Interval]
-------------+----------------------------------------------------------------
anzahlopge~t |   1.348575   .0926785     4.35   0.000      1.17863    1.543023
------------------------------------------------------------------------------
```

```
282 . xi: stcox abtraeger

          failure _d:  amputation
    analysis time _t:  (coxend-origin)
              origin:  time dateop
                  id:  id

    Iteration 0:   log likelihood = -314.28893
    Iteration 1:   log likelihood = -313.36371
    Iteration 2:   log likelihood = -312.20252
    Iteration 3:   log likelihood = -312.16705
    Iteration 4:   log likelihood = -312.16699
    Refining estimates:
    Iteration 0:   log likelihood = -312.16699

    Cox regression -- Breslow method for ties

    No. of subjects =          595                Number of obs    =         595
    No. of failures =           58
    Time at risk    =       644772
                                                  LR chi2(1)       =        4.24
    Log likelihood  =   -312.16699                Prob > chi2      =      0.0394

    ------------------------------------------------------------------------------
              _t | Haz. Ratio   Std. Err.      z    P>|z|     [95% Conf. Interval]
    -------------+----------------------------------------------------------------
       abtraeger |   2.760603   1.196096     2.34   0.019     1.180867    6.453672
    ------------------------------------------------------------------------------

283 . xi: stcox opdauer

          failure _d:  amputation
    analysis time _t:  (coxend-origin)
              origin:  time dateop
                  id:  id

    Iteration 0:   log likelihood = -309.10086
    Iteration 1:   log likelihood =  -308.6706
    Iteration 2:   log likelihood = -305.13861
    Iteration 3:   log likelihood = -304.79901
    Iteration 4:   log likelihood = -304.79313
    Iteration 5:   log likelihood = -304.79313
    Refining estimates:
    Iteration 0:   log likelihood = -304.79313

    Cox regression -- Breslow method for ties

    No. of subjects =          558                Number of obs    =         558
    No. of failures =           58
    Time at risk    =       601858
                                                  LR chi2(1)       =        8.62
    Log likelihood  =   -304.79313                Prob > chi2      =      0.0033

    ------------------------------------------------------------------------------
              _t | Haz. Ratio   Std. Err.      z    P>|z|     [95% Conf. Interval]
    -------------+----------------------------------------------------------------
         opdauer |    1134025    4503944     3.51   0.000     472.0129    2.72e+09
    ------------------------------------------------------------------------------

284 . xi: stcox opdauergrp

          failure _d:  amputation
    analysis time _t:  (coxend-origin)
              origin:  time dateop
                  id:  id
```

```
Iteration 0:   log likelihood = -314.28893
Iteration 1:   log likelihood = -313.82346
Iteration 2:   log likelihood =  -313.8226
Iteration 3:   log likelihood =  -313.8226
Refining estimates:
Iteration 0:   log likelihood =  -313.8226

Cox regression -- Breslow method for ties

No. of subjects =          595                 Number of obs    =          595
No. of failures =           58
Time at risk    =       644772
                                               LR chi2(1)       =         0.93
Log likelihood  =    -313.8226                 Prob > chi2      =       0.3342
```

_t	Haz. Ratio	Std. Err.	z	P>|z|	[95% Conf.	Interval]
opdauergrp	1.17366	.1979321	0.95	0.342	.8433179	1.633403

```
285 . xi: stcox ruhigstellung

         failure _d:  amputation
   analysis time _t:  (coxend-origin)
             origin:  time dateop
                 id:  id

Iteration 0:   log likelihood =  -287.0085
Iteration 1:   log likelihood = -282.87765
Iteration 2:   log likelihood = -282.84179
Refining estimates:
Iteration 0:   log likelihood = -282.84179

Cox regression -- Breslow method for ties

No. of subjects =          552                 Number of obs    =          552
No. of failures =           54
Time at risk    =       597322
                                               LR chi2(1)       =         8.33
Log likelihood  =   -282.84179                 Prob > chi2      =       0.0039
```

_t	Haz. Ratio	Std. Err.	z	P>|z|	[95% Conf.	Interval]
ruhigstell~g	2.180317	.5826962	2.92	0.004	1.291318	3.681338

```
286 . xi: stcox knochenresektion

         failure _d:  amputation
   analysis time _t:  (coxend-origin)
             origin:  time dateop
                 id:  id

Iteration 0:   log likelihood = -144.81194
Iteration 1:   log likelihood = -143.87566
Iteration 2:   log likelihood = -143.87271
Refining estimates:
Iteration 0:   log likelihood = -143.87271

Cox regression -- Breslow method for ties

No. of subjects =           49                 Number of obs    =           49
No. of failures =           49
Time at risk    =        52345
                                               LR chi2(1)       =         1.88
Log likelihood  =   -143.87271                 Prob > chi2      =       0.1705
```

```
------------------------------------------------------------------------------
          _t | Haz. Ratio   Std. Err.      z    P>|z|     [95% Conf. Interval]
-------------+----------------------------------------------------------------
knochenres~n |   1.237448   .1899708     1.39   0.165     .9159087    1.671868
------------------------------------------------------------------------------
```

287 . xi: stcox eitergelenk

```
         failure _d:  amputation
   analysis time _t:  (coxend-origin)
             origin:  time dateop
                 id:  id

Iteration 0:   log likelihood = -314.28893
Iteration 1:   log likelihood = -312.84399
Iteration 2:   log likelihood = -312.38606
Iteration 3:   log likelihood = -312.38117
Iteration 4:   log likelihood = -312.38117
Refining estimates:
Iteration 0:   log likelihood = -312.38117

Cox regression -- Breslow method for ties

No. of subjects =          595                  Number of obs    =         595
No. of failures =           58
Time at risk    =       644772
                                                LR chi2(1)       =        3.82
Log likelihood  =   -312.38117                  Prob > chi2      =      0.0508

------------------------------------------------------------------------------
          _t | Haz. Ratio   Std. Err.      z    P>|z|     [95% Conf. Interval]
-------------+----------------------------------------------------------------
 eitergelenk |    .439071     .16763    -2.16   0.031     .2077592    .9279172
------------------------------------------------------------------------------
```

288 . xi: stcox crp

```
         failure _d:  amputation
   analysis time _t:  (coxend-origin)
             origin:  time dateop
                 id:  id

Iteration 0:   log likelihood = -201.52224
Iteration 1:   log likelihood =  -199.3348
Iteration 2:   log likelihood = -197.72707
Iteration 3:   log likelihood = -197.69689
Iteration 4:   log likelihood = -197.69686
Refining estimates:
Iteration 0:   log likelihood = -197.69686

Cox regression -- Breslow method for ties

No. of subjects =          420                  Number of obs    =         420
No. of failures =           39
Time at risk    =       449309
                                                LR chi2(1)       =        7.65
Log likelihood  =   -197.69686                  Prob > chi2      =      0.0057

------------------------------------------------------------------------------
          _t | Haz. Ratio   Std. Err.      z    P>|z|     [95% Conf. Interval]
-------------+----------------------------------------------------------------
         crp |   1.005302   .0017048     3.12   0.002     1.001966    1.008649
------------------------------------------------------------------------------
```

```
289 . xi: stcox sehnenresektion

         failure _d:  amputation
   analysis time _t:  (coxend-origin)
             origin:  time dateop
                 id:  id

Iteration 0:   log likelihood = -314.28893
Iteration 1:   log likelihood = -310.75411
Iteration 2:   log likelihood = -297.44334
Iteration 3:   log likelihood = -289.02986
Iteration 4:   log likelihood = -288.40911
Iteration 5:   log likelihood = -288.40906
Refining estimates:
Iteration 0:   log likelihood = -288.40906

Cox regression -- Breslow method for ties

No. of subjects =          595                Number of obs    =         595
No. of failures =           58
Time at risk    =       644772
                                              LR chi2(1)       =       51.76
Log likelihood  =   -288.40906                Prob > chi2      =      0.0000
```

_t	Haz. Ratio	Std. Err.	z	P>|z|	[95% Conf.	Interval]
sehnenrese~n	.1267217	.0334989	-7.81	0.000	.0754807	.2127484

```
290 . xi: stcox gelenkversteifung

         failure _d:  amputation
   analysis time _t:  (coxend-origin)
             origin:  time dateop
                 id:  id

Iteration 0:   log likelihood = -314.28893
Iteration 1:   log likelihood = -310.37642
Iteration 2:   log likelihood = -307.82202
Iteration 3:   log likelihood = -307.70284
Iteration 4:   log likelihood = -307.70223
Refining estimates:
Iteration 0:   log likelihood = -307.70223

Cox regression -- Breslow method for ties

No. of subjects =          595                Number of obs    =         595
No. of failures =           58
Time at risk    =       644772
                                              LR chi2(1)       =       13.17
Log likelihood  =   -307.70223                Prob > chi2      =      0.0003
```

_t	Haz. Ratio	Std. Err.	z	P>|z|	[95% Conf.	Interval]
gelenkvers~g	.1338864	.0581365	-4.63	0.000	.0571644	.3135795

```
291 . xi: stcox wundheilungsstoerung

         failure _d:  amputation
   analysis time _t:  (coxend-origin)
             origin:  time dateop
                 id:  id
```

```
Iteration 0:   log likelihood = -314.28893
Iteration 1:   log likelihood = -310.78363
Iteration 2:   log likelihood = -309.71732
Iteration 3:   log likelihood = -309.71189
Iteration 4:   log likelihood = -309.71189
Refining estimates:
Iteration 0:   log likelihood = -309.71189

Cox regression -- Breslow method for ties

No. of subjects =          595                  Number of obs    =         595
No. of failures =           58
Time at risk    =       644772
                                                LR chi2(1)       =        9.15
Log likelihood  =   -309.71189                  Prob > chi2      =      0.0025
```

_t	Haz. Ratio	Std. Err.	z	P>|z|	[95% Conf. Interval]	
wundheilun~g	2.700135	.8114703	3.31	0.001	1.498216	4.866276

```
292 . xi: stcox i.lappen
i.lappen          _Ilappen_0-4        (naturally coded; _Ilappen_0 omitted)

         failure _d:  amputation
   analysis time _t:  (coxend-origin)
             origin:  time dateop
                 id:  id

Iteration 0:   log likelihood = -314.28893
Iteration 1:   log likelihood = -312.09217
Iteration 2:   log likelihood = -311.62288
Iteration 3:   log likelihood = -311.60265
Iteration 4:   log likelihood = -311.60079
Iteration 5:   log likelihood = -311.60012
Iteration 6:   log likelihood = -311.59987
Iteration 7:   log likelihood = -311.59978
Iteration 8:   log likelihood = -311.59975
Iteration 9:   log likelihood = -311.59974
Iteration 10:  log likelihood = -311.59973
Iteration 11:  log likelihood = -311.59973
Iteration 12:  log likelihood = -311.59973
Iteration 13:  log likelihood = -311.59973
Iteration 14:  log likelihood = -311.59973
Iteration 15:  log likelihood = -311.59973
Iteration 16:  log likelihood = -311.59973
Iteration 17:  log likelihood = -311.59973
Iteration 18:  log likelihood = -311.59973
Iteration 19:  log likelihood = -311.59973
Iteration 20:  log likelihood = -311.59973
Iteration 21:  log likelihood = -311.59973
Iteration 22:  log likelihood = -311.59973
Iteration 23:  log likelihood = -311.59973
Iteration 24:  log likelihood = -311.59973
Iteration 25:  log likelihood = -311.59973
Iteration 26:  log likelihood = -311.59973
Iteration 27:  log likelihood = -311.59973
Iteration 28:  log likelihood = -311.59973
Iteration 29:  log likelihood = -311.59973
Iteration 30:  log likelihood = -311.59973
Iteration 31:  log likelihood = -311.59973
Iteration 32:  log likelihood = -311.59973
Iteration 33:  log likelihood = -311.59973
Iteration 34:  log likelihood = -311.59973
Iteration 35:  log likelihood = -311.59973
Iteration 36:  log likelihood = -311.59973
Iteration 37:  log likelihood = -311.59973
Iteration 38:  log likelihood = -311.59973
Iteration 39:  log likelihood = -311.59973
Iteration 40:  log likelihood = -311.59973
Iteration 41:  log likelihood = -311.59973
```

```
Refining estimates:
Iteration 0:   log likelihood = -311.59973

Cox regression -- Breslow method for ties

No. of subjects =          595                  Number of obs    =         595
No. of failures =           58
Time at risk    =       644772
                                                LR chi2(3)       =        5.38
Log likelihood  =   -311.59973                  Prob > chi2      =      0.1461
```

_t	Haz. Ratio	Std. Err.	z	P>|z|	[95% Conf.	Interval]
_Ilappen_1	1.915361	1.142402	1.09	0.276	.595057	6.165139
_Ilappen_2	2.60e-18	.	.	.	.	.
_Ilappen_3	1.930145	1.400094	0.91	0.365	.4657394	7.999024
_Ilappen_4	6.088289	4.408891	2.49	0.013	1.472617	25.17102

```
293 . xi: stcox samerecurrence

         failure _d:  amputation
   analysis time _t:  (coxend-origin)
             origin:  time dateop
                 id:  id

Iteration 0:   log likelihood = -314.28893
Iteration 1:   log likelihood = -314.18645
Iteration 2:   log likelihood = -314.17419
Iteration 3:   log likelihood = -314.17414
Refining estimates:
Iteration 0:   log likelihood = -314.17414

Cox regression -- Breslow method for ties

No. of subjects =          595                  Number of obs    =         595
No. of failures =           58
Time at risk    =       644772
                                                LR chi2(1)       =        0.23
Log likelihood  =   -314.17414                  Prob > chi2      =      0.6318
```

_t	Haz. Ratio	Std. Err.	z	P>|z|	[95% Conf.	Interval]
samerecurr~e	1.698749	1.736689	0.52	0.604	.2290428	12.59916

```
294 . xi: stcox softtissuecoverage

         failure _d:  amputation
   analysis time _t:  (coxend-origin)
             origin:  time dateop
                 id:  id

Iteration 0:   log likelihood = -314.28893
Iteration 1:   log likelihood = -312.28904
Iteration 2:   log likelihood = -311.48604
Iteration 3:   log likelihood = -311.47693
Iteration 4:   log likelihood = -311.47693
Refining estimates:
Iteration 0:   log likelihood = -311.47693

Cox regression -- Breslow method for ties

No. of subjects =          595                  Number of obs    =         595
No. of failures =           58
Time at risk    =       644772
                                                LR chi2(1)       =        5.62
Log likelihood  =   -311.47693                  Prob > chi2      =      0.0177
```

_t	Haz. Ratio	Std. Err.	z	P>|z|	[95% Conf. Interval]	
softtissue~e	2.49404	.8679424	2.63	0.009	1.260897	4.933183

295 . xi: stcox aureus

```
         failure _d:  amputation
   analysis time _t:  (coxend-origin)
             origin:  time dateop
                 id:  id

Iteration 0:   log likelihood = -314.28893
Iteration 1:   log likelihood = -309.16382
Iteration 2:   log likelihood = -309.07735
Iteration 3:   log likelihood = -309.07735
Refining estimates:
Iteration 0:   log likelihood = -309.07735

Cox regression -- Breslow method for ties

No. of subjects =          595                  Number of obs    =        595
No. of failures =           58
Time at risk    =       644772
                                                LR chi2(1)       =      10.42
Log likelihood  =   -309.07735                  Prob > chi2      =     0.0012
```

_t	Haz. Ratio	Std. Err.	z	P>|z|	[95% Conf. Interval]	
aureus	2.368514	.6233178	3.28	0.001	1.414056	3.96721

296 . xi: stcox gramnegative

```
         failure _d:  amputation
   analysis time _t:  (coxend-origin)
             origin:  time dateop
                 id:  id

Iteration 0:   log likelihood = -314.28893
Iteration 1:   log likelihood = -313.86027
Iteration 2:   log likelihood = -313.85383
Iteration 3:   log likelihood = -313.85383
Refining estimates:
Iteration 0:   log likelihood = -313.85383

Cox regression -- Breslow method for ties

No. of subjects =          595                  Number of obs    =        595
No. of failures =           58
Time at risk    =       644772
                                                LR chi2(1)       =       0.87
Log likelihood  =   -313.85383                  Prob > chi2      =     0.3509
```

_t	Haz. Ratio	Std. Err.	z	P>|z|	[95% Conf. Interval]	
gramnegative	1.383572	.465596	0.96	0.335	.7154146	2.675752

```
297 . xi: stcox strept

         failure _d:  amputation
   analysis time _t:  (coxend-origin)
             origin:  time dateop
                 id:  id

Iteration 0:   log likelihood = -314.28893
Iteration 1:   log likelihood = -314.28512
Iteration 2:   log likelihood = -314.28512
Refining estimates:
Iteration 0:   log likelihood = -314.28512

Cox regression -- Breslow method for ties

No. of subjects =          595                 Number of obs    =         595
No. of failures =           58
Time at risk    =       644772
                                               LR chi2(1)       =        0.01
Log likelihood  =   -314.28512                 Prob > chi2      =      0.9305
```

_t	Haz. Ratio	Std. Err.	z	P>|z|	[95% Conf.	Interval]
strept	.9710971	.3273064	-0.09	0.931	.5016107	1.880003

```
298 .
299 . ** MULTIVARIATE COX REGRESSION AMPUTATION**
300 . xi: stcox sex age crp diabetes aureus gramnegative eitergelenk strept opdauer
> grp anzahlopgesamt

         failure _d:  amputation
   analysis time _t:  (coxend-origin)
             origin:  time dateop
                 id:  id

Iteration 0:   log likelihood = -201.52224
Iteration 1:   log likelihood = -193.39074
Iteration 2:   log likelihood = -182.32749
Iteration 3:   log likelihood = -181.69717
Iteration 4:   log likelihood = -181.69527
Iteration 5:   log likelihood = -181.69527
Refining estimates:
Iteration 0:   log likelihood = -181.69527

Cox regression -- Breslow method for ties

No. of subjects =          420                 Number of obs    =         420
No. of failures =           39
Time at risk    =       449309
                                               LR chi2(10)      =       39.65
Log likelihood  =   -181.69527                 Prob > chi2      =      0.0000
```

_t	Haz. Ratio	Std. Err.	z	P>|z|	[95% Conf.	Interval]
sex	.3230048	.144839	-2.52	0.012	.1341284	.7778529
age	1.026034	.0118718	2.22	0.026	1.003028	1.049568
crp	.9995083	.0024475	-0.20	0.841	.9947229	1.004317
diabetes	2.399359	.9004499	2.33	0.020	1.149872	5.006574
aureus	1.885276	.7080293	1.69	0.091	.9030257	3.935954
gramnegative	1.114843	.6212707	0.20	0.845	.3739925	3.323261
eitergelenk	1.124527	.5585828	0.24	0.813	.4247715	2.97704
strept	1.017983	.4846077	0.04	0.970	.4004315	2.587929
opdauergrp	1.098841	.2543282	0.41	0.684	.6981087	1.729604
anzahlopge~t	1.305567	.142669	2.44	0.015	1.053858	1.617396

```
301 . predict p
    (option hr assumed; predicted hazard ratio)
    (177 missing values generated)

302 . roctab amputation p

                       ROC                    —Asymptotic Normal——
           Obs        Area     Std. Err.      [95% Conf. Interval]
     ——————————————————————————————————————————————————————————————
           423      0.7962       0.0355        0.72662     0.86587

303 . drop p

304 .
305 . *** OUTCOME FAILURE 1
306 . ***Gruppenvergleich
307 . tab sex failure1, chi col

    +-------------------+
    | Key               |
    |-------------------|
    |     frequency     |
    | column percentage |
    +-------------------+

               |       failure1
           sex |         0          1 |     Total
    -----------+----------------------+----------
             1 |       285         77 |       362
               |     57.81      71.96 |     60.33
    -----------+----------------------+----------
             2 |       208         30 |       238
               |     42.19      28.04 |     39.67
    -----------+----------------------+----------
         Total |       493        107 |       600
               |    100.00     100.00 |    100.00

              Pearson chi2(1) =   7.3589   Pr = 0.007

308 . gen agefailure1=age if failure1==1
    (493 missing values generated)

309 . gen agenichts=age if failure1==0
    (107 missing values generated)

310 . centile agefailure1 agenichts

                                                       -- Binom. Interp. --
        Variable |       Obs  Percentile    Centile        [95% Conf. Interval]
    -------------+-------------------------------------------------------------
     agefailure1 |       107         50          59              55          63
       agenichts |       493         50          53              51          54

311 . ranksum age, by (failure1)

    Two-sample Wilcoxon rank-sum (Mann-Whitney) test

        failure1 |      obs    rank sum    expected
    -------------+---------------------------------
               0 |      493    142593.5    148146.5
               1 |      107     37706.5     32153.5
    -------------+---------------------------------
        combined |      600      180300      180300

    unadjusted variance  2641945.92
    adjustment for ties     -899.07
                         ----------
    adjusted variance    2641046.85
```

```
    Ho: age(failure1==0) = age(failure1==1)
                 z =  -3.417
        Prob > |z| =   0.0006

312 . gen bmifailure1=bmi if failure1==1
    (500 missing values generated)

313 . gen bminichts=bmi if failure1==0
    (193 missing values generated)

314 . centile bmifailure1 bminichts

                                                          -- Binom. Interp. --
        Variable |       Obs  Percentile    Centile        [95% Conf. Interval]
    -------------+-------------------------------------------------------------
     bmifailure1 |       100         50          26        24.73648    27.23176
       bminichts |       407         50          26            25.6    26.77762

315 . ranksum bmi, by (failure1)

    Two-sample Wilcoxon rank-sum (Mann-Whitney) test

        failure1 |      obs    rank sum    expected
    -------------+---------------------------------
               0 |      407    103677.5      103378
               1 |      100     25100.5       25400
    -------------+---------------------------------
        combined |      507      128778      128778

    unadjusted variance  1722966.67
    adjustment for ties     -188.24
                         ----------
    adjusted variance    1722778.43

    Ho: bmi(failure1==0) = bmi(failure1==1)
                 z =   0.228
        Prob > |z| =   0.8195

316 . gen crpfailure1=crp if failure1==1
    (519 missing values generated)

317 . gen crpnichts=crp if failure1==0
    (258 missing values generated)

318 . centile crpfailure1 crpnichts

                                                          -- Binom. Interp. --
        Variable |       Obs  Percentile    Centile        [95% Conf. Interval]
    -------------+-------------------------------------------------------------
     crpfailure1 |        81         50        32.7        23.95178    54.23336
       crpnichts |       342         50       14.05        11.93646    16.62708

319 . ranksum crp, by (failure1)

    Two-sample Wilcoxon rank-sum (Mann-Whitney) test

        failure1 |      obs    rank sum    expected
    -------------+---------------------------------
               0 |      342     67665.5       72504
               1 |       81     22010.5       17172
    -------------+---------------------------------
        combined |      423       89676       89676

    unadjusted variance   978804.00
    adjustment for ties      -25.53
                         ----------
    adjusted variance     978778.47

    Ho: crp(failure1==0) = crp(failure1==1)
                 z =  -4.891
        Prob > |z| =   0.0000
```

```
320 . gen opdauerfailure1=opdauer if failure1==1
    (493 missing values generated)

321 . gen opdauernichts=opdauer if failure1==0
    (149 missing values generated)

322 . centile opdauerfailure1 opdauernichts

                                                          -- Binom. Interp. --
        Variable |       Obs  Percentile    Centile        [95% Conf. Interval]
    -------------+-------------------------------------------------------------
    opdauerfai~1 |       107         50    .0243056        .0207394    .0284722
    opdauernic~s |       451         50    .0173611        .0159722      .01875

323 . ranksum opdauer, by (failure1)

    Two-sample Wilcoxon rank-sum (Mann-Whitney) test

        failure1 |      obs    rank sum    expected
    -------------+---------------------------------
               0 |      451    117983.5    126054.5
               1 |      107     37977.5     29906.5
    -------------+---------------------------------
        combined |      558      155961      155961

    unadjusted variance  2247971.92
    adjustment for ties    -1072.41
                         ----------
    adjusted variance    2246899.51

    Ho: opdauer(failure1==0) = opdauer(failure1==1)
                 z =  -5.384
        Prob > |z| =   0.0000

324 . gen anzahlopgesamtfailure1=anzahlopgesamt if failure1==1
    (493 missing values generated)

325 . gen anzahlopgesamtnichts=anzahlopgesamt if failure1==0
    (107 missing values generated)

326 . centile anzahlopgesamtfailure1 anzahlopgesamtnichts

                                                          -- Binom. Interp. --
        Variable |       Obs  Percentile    Centile        [95% Conf. Interval]
    -------------+-------------------------------------------------------------
    anzahlopge~1 |       107         50           3               2           3
    anzahlopge~s |       493         50           1               1           1

327 . ranksum anzahlopgesamt, by (failure1)

    Two-sample Wilcoxon rank-sum (Mann-Whitney) test

        failure1 |      obs    rank sum    expected
    -------------+---------------------------------
               0 |      493    131721.5    148146.5
               1 |      107     48578.5     32153.5
    -------------+---------------------------------
        combined |      600      180300      180300

    unadjusted variance  2641945.92
    adjustment for ties  -972295.38
                         ----------
    adjusted variance    1669650.54

    Ho: anzahl~t(failure1==0) = anzahl~t(failure1==1)
                 z = -12.711
        Prob > |z| =   0.0000
```

```
328 . tab abtraeger failure1, chi col
```

Key
frequency
column percentage

abtraeger	failure1 0	1	Total
0	**479**<br>**97.16**	**99**<br>**92.52**	**578**<br>**96.33**
1	**14**<br>**2.84**	**8**<br>**7.48**	**22**<br>**3.67**
Total	**493**<br>**100.00**	**107**<br>**100.00**	**600**<br>**100.00**

Pearson chi2(**1**) = **5.3516** Pr = **0.021**

```
329 . tab sehnenresektion failure1, chi col
```

Key
frequency
column percentage

sehnenrese ktion	failure1 0	1	Total
1	**19**<br>**3.85**	**37**<br>**34.58**	**56**<br>**9.33**
2	**474**<br>**96.15**	**70**<br>**65.42**	**544**<br>**90.67**
Total	**493**<br>**100.00**	**107**<br>**100.00**	**600**<br>**100.00**

Pearson chi2(**1**) = **98.0827** Pr = **0.000**

```
330 . tab knochenresektion failure1, chi col
```

Key
frequency
column percentage

knochenres ektion	failure1 1	Total
1	**29**<br>**59.18**	**29**<br>**59.18**
2	**1**<br>**2.04**	**1**<br>**2.04**
3	**19**<br>**38.78**	**19**<br>**38.78**
Total	**49**<br>**100.00**	**49**<br>**100.00**

331 . tab wundheilungsstoerung failure1, chi col

Key
frequency   *column percentage*

wundheilungsstoerung	failure1 0	1	Total
0	**472** **95.74**	**55** **51.40**	**527** **87.83**
1	**21** **4.26**	**52** **48.60**	**73** **12.17**
Total	**493** **100.00**	**107** **100.00**	**600** **100.00**

Pearson chi2(**1**) = **161.7374**   Pr = **0.000**

332 . tab aureus failure1, chi col

Key
frequency   *column percentage*

aureus	failure1 0	1	Total
0	**362** **73.43**	**60** **56.07**	**422** **70.33**
1	**131** **26.57**	**47** **43.93**	**178** **29.67**
Total	**493** **100.00**	**107** **100.00**	**600** **100.00**

Pearson chi2(**1**) = **12.6885**   Pr = **0.000**

333 . tab gramnegative failure1, chi col

Key
frequency   *column percentage*

gramnegative	failure1 0	1	Total
0	**433** **87.83**	**89** **83.18**	**522** **87.00**
1	**60** **12.17**	**18** **16.82**	**78** **13.00**
Total	**493** **100.00**	**107** **100.00**	**600** **100.00**

Pearson chi2(**1**) = **1.6823**   Pr = **0.195**

334 . tab strept failure1, chi col

Key
frequency   *column percentage*

strept	failure1 0	1	Total
0	**423** **85.80**	**82** **76.64**	**505** **84.17**
1	**70** **14.20**	**25** **23.36**	**95** **15.83**
Total	**493** **100.00**	**107** **100.00**	**600** **100.00**

Pearson chi2(**1**) = **5.5424** Pr = **0.019**

335 . tab diabetes failure1, chi col

Key
frequency   *column percentage*

diabetes	failure1 0	1	Total
0	**451** **91.48**	**78** **72.90**	**529** **88.17**
1	**42** **8.52**	**29** **27.10**	**71** **11.83**
Total	**493** **100.00**	**107** **100.00**	**600** **100.00**

Pearson chi2(**1**) = **29.1021** Pr = **0.000**

336 . tab eitergelenk failure1, chi col

Key
frequency   *column percentage*

eitergelen k	failure1 0	1	Total
1	**20** **4.06**	**21** **19.63**	**41** **6.83**
2	**473** **95.94**	**86** **80.37**	**559** **93.17**
Total	**493** **100.00**	**107** **100.00**	**600** **100.00**

Pearson chi2(**1**) = **33.4756** Pr = **0.000**

```
337 . tab gelenkversteifung softtissuecoverage, chi col
```

Key
frequency
column percentage

gelenkversteifung	softtissuecoverage 0	1	Total
1	**8**	**1**	**9**
	**1.45**	**2.08**	**1.50**
2	**544**	**47**	**591**
	**98.55**	**97.92**	**98.50**
Total	**552**	**48**	**600**
	**100.00**	**100.00**	**100.00**

Pearson chi2(**1**) = **0.1202** Pr = **0.729**

```
338 . gen abfailure1=dauerabtotal if failure1==1
    (535 missing values generated)

339 . gen abnichts=dauerabtotal if failure1==0
    (211 missing values generated)

340 . centile abfailure1 abnichts
```

Variable	Obs	Percentile	Centile	Binom. Interp. [95% Conf.	Interval]
abfailure1	**65**	**50**	**12**	**9**	**14.89678**
abnichts	**389**	**50**	**8**	**8**	**9**

```
341 . ranksum dauerabtotal, by (failure1)
```

Two-sample Wilcoxon rank-sum (Mann-Whitney) test

failure1	obs	rank sum	expected
0	**389**	**84395.5**	**88497.5**
1	**65**	**18889.5**	**14787.5**
combined	**454**	**103285**	**103285**

unadjusted variance	**958722.92**
adjustment for ties	**-13492.85**
adjusted variance	**945230.07**

Ho: dauera~l(failure1==0) = dauera~l(failure1==1)
z = **-4.219**
Prob > |z| = **0.0000**

```
342 . tab dauerabgrp failure1, chi col
```

Key
frequency
column percentage

dauerabgrp	failure1 0	1	Total
1	**173**<br>**35.09**	**17**<br>**15.89**	**190**<br>**31.67**
2	**117**<br>**23.73**	**11**<br>**10.28**	**128**<br>**21.33**
3	**203**<br>**41.18**	**79**<br>**73.83**	**282**<br>**47.00**
Total	**493**<br>**100.00**	**107**<br>**100.00**	**600**<br>**100.00**

Pearson chi2(**2**) = **37.6434** Pr = **0.000**

```
343 . tab ruhigstellung failure1, chi col
```

Key
frequency<br>*column percentage*

ruhigstellung	failure1 0	1	Total
1	**280**<br>**61.81**	**53**<br>**53.00**	**333**<br>**60.22**
2	**173**<br>**38.19**	**46**<br>**46.00**	**219**<br>**39.60**
3	**0**<br>**0.00**	**1**<br>**1.00**	**1**<br>**0.18**
Total	**453**<br>**100.00**	**100**<br>**100.00**	**553**<br>**100.00**

Pearson chi2(**2**) = **6.8476** Pr = **0.033**

```
344 . drop crpfailure1 crpnichts agefailure1 agenichts

345 . drop anzahlopgesamtfailure1 anzahlopgesamtnichts opdauerfailure1

346 . drop abfailure1 abnichts bmifailure1 bminichts opdauernichts

347 .
348 . ** UNIVARIATE COX Regression FAILURE1 *
349 . stset coxend, fail (failure1) origin (dateop) id (id)

                id:  id
     failure event:  failure1 != 0 & failure1 < .
obs. time interval:  (coxend[_n-1], coxend]
 exit on or before:  failure
    t for analysis:  (time-origin)
            origin:  time dateop

------------------------------------------------------------------------------
        600  total observations
          5  ignored because never entered
------------------------------------------------------------------------------
        595  observations remaining, representing
        595  subjects
        107  failures in single-failure-per-subject data
    644,772  total analysis time at risk and under observation
                                                at risk from t =         0
                                     earliest observed entry t =         0
                                          last observed exit t =     1,427
```

```
350 . xi: stcox sex

         failure _d:  failure1
   analysis time _t:  (coxend-origin)
             origin:  time dateop
                 id:  id

Iteration 0:   log likelihood =  -583.5118
Iteration 1:   log likelihood = -579.49205
Iteration 2:   log likelihood = -579.47131
Iteration 3:   log likelihood = -579.47131
Refining estimates:
Iteration 0:   log likelihood = -579.47131

Cox regression -- Breslow method for ties

No. of subjects =          595                  Number of obs    =         595
No. of failures =          107
Time at risk    =       644772
                                                LR chi2(1)       =        8.08
Log likelihood  =   -579.47131                  Prob > chi2      =      0.0045
```

_t	Haz. Ratio	Std. Err.	z	P>|z|	[95% Conf.	Interval]
sex	.5544709	.1194229	-2.74	0.006	.3635344	.8456915

```
351 . xi: stcox age

         failure _d:  failure1
   analysis time _t:  (coxend-origin)
             origin:  time dateop
                 id:  id

Iteration 0:   log likelihood =  -583.5118
Iteration 1:   log likelihood = -577.64146
Iteration 2:   log likelihood = -577.63498
Refining estimates:
Iteration 0:   log likelihood = -577.63498

Cox regression -- Breslow method for ties

No. of subjects =          595                  Number of obs    =         595
No. of failures =          107
Time at risk    =       644772
                                                LR chi2(1)       =       11.75
Log likelihood  =   -577.63498                  Prob > chi2      =      0.0006
```

_t	Haz. Ratio	Std. Err.	z	P>|z|	[95% Conf.	Interval]
age	1.01815	.0054287	3.37	0.001	1.007566	1.028846

```
352 . xi: stcox bmi

         failure _d:  failure1
   analysis time _t:  (coxend-origin)
             origin:  time dateop
                 id:  id

Iteration 0:   log likelihood = -531.49051
Iteration 1:   log likelihood = -531.43508
Iteration 2:   log likelihood = -531.43507
Refining estimates:
Iteration 0:   log likelihood = -531.43507

Cox regression -- Breslow method for ties
```

```
No. of subjects =          507              Number of obs    =        507
No. of failures =          100
Time at risk    =       547711
                                            LR chi2(1)       =       0.11
Log likelihood  =   -531.43507              Prob > chi2      =     0.7391
```

_t	Haz. Ratio	Std. Err.	z	P>|z|	[95% Conf.	Interval]
bmi	1.006634	.0198782	0.33	0.738	.9684176	1.046358

353 . xi: stcox diabetes

```
         failure _d:  failure1
   analysis time _t:  (coxend-origin)
             origin:  time dateop
                 id:  id

Iteration 0:   log likelihood =  -583.5118
Iteration 1:   log likelihood = -576.70091
Iteration 2:   log likelihood = -570.54393
Iteration 3:   log likelihood = -570.50485
Iteration 4:   log likelihood = -570.50485
Refining estimates:
Iteration 0:   log likelihood = -570.50485

Cox regression -- Breslow method for ties

No. of subjects =          595              Number of obs    =        595
No. of failures =          107
Time at risk    =       644772
                                            LR chi2(1)       =      26.01
Log likelihood  =   -570.50485              Prob > chi2      =     0.0000
```

_t	Haz. Ratio	Std. Err.	z	P>|z|	[95% Conf.	Interval]
diabetes	3.453618	.7535121	5.68	0.000	2.25195	5.296511

354 . xi: stcox dauerabgrp

```
         failure _d:  failure1
   analysis time _t:  (coxend-origin)
             origin:  time dateop
                 id:  id

Iteration 0:   log likelihood =  -583.5118
Iteration 1:   log likelihood = -567.91873
Iteration 2:   log likelihood = -567.60295
Iteration 3:   log likelihood = -567.60228
Refining estimates:
Iteration 0:   log likelihood = -567.60228

Cox regression -- Breslow method for ties

No. of subjects =          595              Number of obs    =        595
No. of failures =          107
Time at risk    =       644772
                                            LR chi2(1)       =      31.82
Log likelihood  =   -567.60228              Prob > chi2      =     0.0000
```

_t	Haz. Ratio	Std. Err.	z	P>|z|	[95% Conf.	Interval]
dauerabgrp	2.033777	.2821001	5.12	0.000	1.549656	2.66914

```
355 . xi: stcox dauerabtotal

         failure _d:  failure1
   analysis time _t:  (coxend-origin)
             origin:  time dateop
                 id:  id

Iteration 0:   log likelihood =   -334.042
Iteration 1:   log likelihood = -321.55745
Iteration 2:   log likelihood = -321.47204
Iteration 3:   log likelihood = -321.47193
Refining estimates:
Iteration 0:   log likelihood = -321.47193

Cox regression -- Breslow method for ties

No. of subjects =          449                Number of obs    =         449
No. of failures =           65
Time at risk    =       489102
                                              LR chi2(1)       =       25.14
Log likelihood  =   -321.47193                Prob > chi2      =      0.0000
```

_t	Haz. Ratio	Std. Err.	z	P>|z|	[95% Conf.	Interval]
dauerabtotal	1.043813	.0073604	6.08	0.000	1.029486	1.058339

```
356 . xi: stcox anzahlopgesamt

         failure _d:  failure1
   analysis time _t:  (coxend-origin)
             origin:  time dateop
                 id:  id

Iteration 0:   log likelihood =  -583.5118
Iteration 1:   log likelihood =  -551.5308
Iteration 2:   log likelihood = -544.76317
Iteration 3:   log likelihood = -544.65032
Iteration 4:   log likelihood = -544.65024
Refining estimates:
Iteration 0:   log likelihood = -544.65024

Cox regression -- Breslow method for ties

No. of subjects =          595                Number of obs    =         595
No. of failures =          107
Time at risk    =       644772
                                              LR chi2(1)       =       77.72
Log likelihood  =   -544.65024                Prob > chi2      =      0.0000
```

_t	Haz. Ratio	Std. Err.	z	P>|z|	[95% Conf.	Interval]
anzahlopge~t	1.555396	.0616477	11.15	0.000	1.439142	1.68104

```
357 . xi: stcox abtraeger

         failure _d:  failure1
   analysis time _t:  (coxend-origin)
             origin:  time dateop
                 id:  id

Iteration 0:   log likelihood =  -583.5118
Iteration 1:   log likelihood = -582.32507
Iteration 2:   log likelihood = -582.04025
Iteration 3:   log likelihood = -582.03748
Iteration 4:   log likelihood = -582.03748
Refining estimates:
Iteration 0:   log likelihood = -582.03748
```

```
Cox regression -- Breslow method for ties

No. of subjects =          595                  Number of obs    =         595
No. of failures =          107
Time at risk    =       644772
                                                LR chi2(1)       =        2.95
Log likelihood  =   -582.03748                  Prob > chi2      =      0.0860

------------------------------------------------------------------------------
          _t | Haz. Ratio   Std. Err.      z    P>|z|     [95% Conf. Interval]
-------------+----------------------------------------------------------------
   abtraeger |   2.006431   .7399092     1.89   0.059     .9739298    4.133528
------------------------------------------------------------------------------

358 . xi: stcox opdauer

         failure _d:  failure1
   analysis time _t:  (coxend-origin)
             origin:  time dateop
                 id:  id

Iteration 0:   log likelihood = -573.65716
Iteration 1:   log likelihood = -565.21564
Iteration 2:   log likelihood = -564.97668
Iteration 3:   log likelihood =  -564.9741
Iteration 4:   log likelihood =  -564.9741
Refining estimates:
Iteration 0:   log likelihood =  -564.9741

Cox regression -- Breslow method for ties

No. of subjects =          558                  Number of obs    =         558
No. of failures =          107
Time at risk    =       601858
                                                LR chi2(1)       =       17.37
Log likelihood  =    -564.9741                  Prob > chi2      =      0.0000

------------------------------------------------------------------------------
          _t | Haz. Ratio   Std. Err.      z    P>|z|     [95% Conf. Interval]
-------------+----------------------------------------------------------------
     opdauer |    1728305    4943109     5.02   0.000     6354.877    4.70e+08
------------------------------------------------------------------------------

359 . xi: stcox opdauergrp

         failure _d:  failure1
   analysis time _t:  (coxend-origin)
             origin:  time dateop
                 id:  id

Iteration 0:   log likelihood =  -583.5118
Iteration 1:   log likelihood = -579.04025
Iteration 2:   log likelihood =  -578.9867
Iteration 3:   log likelihood = -578.98669
Refining estimates:
Iteration 0:   log likelihood = -578.98669

Cox regression -- Breslow method for ties

No. of subjects =          595                  Number of obs    =         595
No. of failures =          107
Time at risk    =       644772
                                                LR chi2(1)       =        9.05
Log likelihood  =   -578.98669                  Prob > chi2      =      0.0026

------------------------------------------------------------------------------
          _t | Haz. Ratio   Std. Err.      z    P>|z|     [95% Conf. Interval]
-------------+----------------------------------------------------------------
  opdauergrp |   1.473163   .1998447     2.86   0.004     1.129222    1.921862
------------------------------------------------------------------------------
```

```
360 . xi: stcox ruhigstellung

         failure _d:  failure1
   analysis time _t:  (coxend-origin)
             origin:  time dateop
                 id:  id

    Iteration 0:   log likelihood =  -533.6759
    Iteration 1:   log likelihood = -529.81989
    Iteration 2:   log likelihood = -529.79667
    Iteration 3:   log likelihood = -529.79667
    Refining estimates:
    Iteration 0:   log likelihood = -529.79667

    Cox regression -- Breslow method for ties

    No. of subjects =          552                Number of obs    =         552
    No. of failures =          100
    Time at risk    =       597322
                                                  LR chi2(1)       =        7.76
    Log likelihood  =   -529.79667                Prob > chi2      =      0.0053
```

_t	Haz. Ratio	Std. Err.	z	P>|z|	[95% Conf.	Interval]
ruhigstell~g	1.744	.3431335	2.83	0.005	1.185965	2.564609

```
361 . xi: stcox knochenresektion

         failure _d:  failure1
   analysis time _t:  (coxend-origin)
             origin:  time dateop
                 id:  id

    Iteration 0:   log likelihood = -144.81194
    Iteration 1:   log likelihood = -143.87566
    Iteration 2:   log likelihood = -143.87271
    Refining estimates:
    Iteration 0:   log likelihood = -143.87271

    Cox regression -- Breslow method for ties

    No. of subjects =           49                Number of obs    =          49
    No. of failures =           49
    Time at risk    =        52345
                                                  LR chi2(1)       =        1.88
    Log likelihood  =   -143.87271                Prob > chi2      =      0.1705
```

_t	Haz. Ratio	Std. Err.	z	P>|z|	[95% Conf.	Interval]
knochenres~n	1.237448	.1899708	1.39	0.165	.9159087	1.671868

```
362 . xi: stcox eitergelenk

         failure _d:  failure1
   analysis time _t:  (coxend-origin)
             origin:  time dateop
                 id:  id

    Iteration 0:   log likelihood =  -583.5118
    Iteration 1:   log likelihood = -581.15787
    Iteration 2:   log likelihood = -573.50914
    Iteration 3:   log likelihood = -573.36175
    Iteration 4:   log likelihood = -573.36156
    Refining estimates:
    Iteration 0:   log likelihood = -573.36156

    Cox regression -- Breslow method for ties
```

```
No. of subjects =          595                 Number of obs    =        595
No. of failures =          107
Time at risk    =       644772
                                               LR chi2(1)       =      20.30
Log likelihood  =   -573.36156                 Prob > chi2      =     0.0000
```

_t	Haz. Ratio	Std. Err.	z	P>|z|	[95% Conf.	Interval]
eitergelenk	.2847617	.0695863	-5.14	0.000	.1763908	.4597133

```
363 . xi: stcox crp

          failure _d:  failure1
    analysis time _t:  (coxend-origin)
              origin:  time dateop
                  id:  id

Iteration 0:   log likelihood = -421.11817
Iteration 1:   log likelihood = -416.59369
Iteration 2:   log likelihood = -410.31202
Iteration 3:   log likelihood =  -410.1922
Iteration 4:   log likelihood = -410.19208
Refining estimates:
Iteration 0:   log likelihood = -410.19208

Cox regression -- Breslow method for ties

No. of subjects =          420                 Number of obs    =        420
No. of failures =           81
Time at risk    =       449309
                                               LR chi2(1)       =      21.85
Log likelihood  =   -410.19208                 Prob > chi2      =     0.0000
```

_t	Haz. Ratio	Std. Err.	z	P>|z|	[95% Conf.	Interval]
crp	1.005978	.0011276	5.32	0.000	1.00377	1.00819

```
364 . xi: stcox sehnenresektion

          failure _d:  failure1
    analysis time _t:  (coxend-origin)
              origin:  time dateop
                  id:  id

Iteration 0:   log likelihood =  -583.5118
Iteration 1:   log likelihood = -574.95469
Iteration 2:   log likelihood = -561.44668
Iteration 3:   log likelihood = -561.44407
Refining estimates:
Iteration 0:   log likelihood = -561.44407

Cox regression -- Breslow method for ties

No. of subjects =          595                 Number of obs    =        595
No. of failures =          107
Time at risk    =       644772
                                               LR chi2(1)       =      44.14
Log likelihood  =   -561.44407                 Prob > chi2      =     0.0000
```

_t	Haz. Ratio	Std. Err.	z	P>|z|	[95% Conf.	Interval]
sehnenrese~n	.2209054	.0452668	-7.37	0.000	.1478368	.3300883

```
365 . xi: stcox gelenkversteifung

         failure _d:  failure1
   analysis time _t:  (coxend-origin)
             origin:  time dateop
                 id:  id

    Iteration 0:   log likelihood =  -583.5118
    Iteration 1:   log likelihood = -580.01316
    Iteration 2:   log likelihood = -580.01315
    Refining estimates:
    Iteration 0:   log likelihood = -580.01315

    Cox regression -- Breslow method for ties

    No. of subjects =          595                Number of obs    =         595
    No. of failures =          107
    Time at risk    =       644772
                                                  LR chi2(1)       =        7.00
    Log likelihood  =   -580.01315                Prob > chi2      =      0.0082
```

_t	Haz. Ratio	Std. Err.	z	P>|z|	[95% Conf.	Interval]
gelenkvers~g	.2621213	.1105953	-3.17	0.002	.1146463	.5993006

```
366 . xi: stcox wundheilungsstoerung

         failure _d:  failure1
   analysis time _t:  (coxend-origin)
             origin:  time dateop
                 id:  id

    Iteration 0:   log likelihood =  -583.5118
    Iteration 1:   log likelihood = -573.07332
    Iteration 2:   log likelihood = -547.95311
    Iteration 3:   log likelihood = -537.80435
    Iteration 4:   log likelihood = -537.55282
    Refining estimates:
    Iteration 0:   log likelihood = -537.55282

    Cox regression -- Breslow method for ties

    No. of subjects =          595                Number of obs    =         595
    No. of failures =          107
    Time at risk    =       644772
                                                  LR chi2(1)       =       91.92
    Log likelihood  =   -537.55282                Prob > chi2      =      0.0000
```

_t	Haz. Ratio	Std. Err.	z	P>|z|	[95% Conf.	Interval]
wundheilun~g	7.472286	1.44947	10.37	0.000	5.109023	10.92872

```
367 . xi: stcox i.lappen
    i.lappen          _Ilappen_0-4        (naturally coded; _Ilappen_0 omitted)

         failure _d:  failure1
   analysis time _t:  (coxend-origin)
             origin:  time dateop
                 id:  id
```

```
Iteration 0:   log likelihood =  -583.5118
Iteration 1:   log likelihood = -567.78508
Iteration 2:   log likelihood = -562.63441
Iteration 3:   log likelihood = -561.82164
Iteration 4:   log likelihood = -561.81847
Iteration 5:   log likelihood = -561.81847
Refining estimates:
Iteration 0:   log likelihood = -561.81847

Cox regression -- Breslow method for ties

No. of subjects =          595                  Number of obs    =         595
No. of failures =          107
Time at risk    =       644772
                                                LR chi2(4)       =       43.39
Log likelihood  =   -561.81847                  Prob > chi2      =      0.0000
```

_t	Haz. Ratio	Std. Err.	z	P>|z|	[95% Conf.	Interval]
_Ilappen_1	4.827347	1.505369	5.05	0.000	2.6198	8.895058
_Ilappen_2	20.92626	21.2994	2.99	0.003	2.846507	153.8406
_Ilappen_3	5.629371	2.012399	4.83	0.000	2.793641	11.34355
_Ilappen_4	7.5604	3.888067	3.93	0.000	2.759304	20.71524

368 . xi: stcox samerecurrence

```
         failure _d:  failure1
   analysis time _t:  (coxend-origin)
             origin:  time dateop
                 id:  id

Iteration 0:   log likelihood =  -583.5118
Iteration 1:   log likelihood = -583.21389
Iteration 2:   log likelihood = -582.41665
Iteration 3:   log likelihood = -582.37599
Iteration 4:   log likelihood = -582.37581
Refining estimates:
Iteration 0:   log likelihood = -582.37581

Cox regression -- Breslow method for ties

No. of subjects =          595                  Number of obs    =         595
No. of failures =          107
Time at risk    =       644772
                                                LR chi2(1)       =        2.27
Log likelihood  =   -582.37581                  Prob > chi2      =      0.1317
```

_t	Haz. Ratio	Std. Err.	z	P>|z|	[95% Conf.	Interval]
samerecurr~e	2.843356	1.710129	1.74	0.082	.8747388	9.242388

369 . xi: stcox softtissuecoverage

```
         failure _d:  failure1
   analysis time _t:  (coxend-origin)
             origin:  time dateop
                 id:  id

Iteration 0:   log likelihood =  -583.5118
Iteration 1:   log likelihood = -530.79939
Iteration 2:   log likelihood = -529.40018
Iteration 3:   log likelihood = -529.40017
Refining estimates:
Iteration 0:   log likelihood = -529.40017

Cox regression -- Breslow method for ties
```

```
No. of subjects =          595                  Number of obs    =        595
No. of failures =          107
Time at risk    =       644772
                                                LR chi2(1)       =     108.22
Log likelihood  =   -529.40017                  Prob > chi2      =     0.0000
```

_t	Haz. Ratio	Std. Err.	z	P>|z|	[95% Conf.	Interval]
softtissue~e	9.761758	1.901553	11.70	0.000	6.663728	14.30009

370 . xi: stcox aureus

```
         failure _d:  failure1
   analysis time _t:  (coxend-origin)
             origin:  time dateop
                 id:  id

Iteration 0:   log likelihood =  -583.5118
Iteration 1:   log likelihood =  -578.5446
Iteration 2:   log likelihood =  -578.4858
Iteration 3:   log likelihood =  -578.4858
Refining estimates:
Iteration 0:   log likelihood =  -578.4858

Cox regression -- Breslow method for ties

No. of subjects =          595                  Number of obs    =        595
No. of failures =          107
Time at risk    =       644772
                                                LR chi2(1)       =      10.05
Log likelihood  =    -578.4858                  Prob > chi2      =     0.0015
```

_t	Haz. Ratio	Std. Err.	z	P>|z|	[95% Conf.	Interval]
aureus	1.881198	.3674703	3.23	0.001	1.28281	2.758716

371 . xi: stcox gramnegative

```
         failure _d:  failure1
   analysis time _t:  (coxend-origin)
             origin:  time dateop
                 id:  id

Iteration 0:   log likelihood =  -583.5118
Iteration 1:   log likelihood = -583.22387
Iteration 2:   log likelihood = -583.22225
Iteration 3:   log likelihood = -583.22225
Refining estimates:
Iteration 0:   log likelihood = -583.22225

Cox regression -- Breslow method for ties

No. of subjects =          595                  Number of obs    =        595
No. of failures =          107
Time at risk    =       644772
                                                LR chi2(1)       =       0.58
Log likelihood  =   -583.22225                  Prob > chi2      =     0.4467
```

_t	Haz. Ratio	Std. Err.	z	P>|z|	[95% Conf.	Interval]
gramnegative	1.223616	.3173702	0.78	0.437	.735984	2.034332

```
372 . xi: stcox strept

         failure _d:  failure1
   analysis time _t:  (coxend-origin)
             origin:  time dateop
                 id:  id

Iteration 0:   log likelihood =  -583.5118
Iteration 1:   log likelihood = -582.97605
Iteration 2:   log likelihood = -582.97299
Iteration 3:   log likelihood = -582.97299
Refining estimates:
Iteration 0:   log likelihood = -582.97299

Cox regression -- Breslow method for ties

No. of subjects =          595                  Number of obs    =         595
No. of failures =          107
Time at risk    =       644772
                                                LR chi2(1)       =        1.08
Log likelihood  =   -582.97299                  Prob > chi2      =      0.2992

------------------------------------------------------------------------------
          _t | Haz. Ratio   Std. Err.      z    P>|z|     [95% Conf. Interval]
-------------+----------------------------------------------------------------
      strept |   1.276324   .2935121     1.06   0.289     .8132289    2.003129
------------------------------------------------------------------------------

373 .
374 . ** MULTIVARIATE COX REGRESSION FAILURE1**
375 . xi: stcox sex age crp diabetes aureus gramnegative eitergelenk strept anzahlo
> pgesamt

         failure _d:  failure1
   analysis time _t:  (coxend-origin)
             origin:  time dateop
                 id:  id

Iteration 0:   log likelihood = -421.11817
Iteration 1:   log likelihood = -382.47403
Iteration 2:   log likelihood = -374.97131
Iteration 3:   log likelihood =  -374.8752
Iteration 4:   log likelihood = -374.87507
Refining estimates:
Iteration 0:   log likelihood = -374.87507

Cox regression -- Breslow method for ties

No. of subjects =          420                  Number of obs    =         420
No. of failures =           81
Time at risk    =       449309
                                                LR chi2(9)       =       92.49
Log likelihood  =   -374.87507                  Prob > chi2      =      0.0000

------------------------------------------------------------------------------
          _t | Haz. Ratio   Std. Err.      z    P>|z|     [95% Conf. Interval]
-------------+----------------------------------------------------------------
         sex |   .4806043   .1362722    -2.58   0.010     .2756988       .8378
         age |   1.020427   .0079445     2.60   0.009     1.004974    1.036117
         crp |   .9985284   .0016778    -0.88   0.381     .9952454    1.001822
    diabetes |   1.675801   .4702222     1.84   0.066     .9668949    2.904463
      aureus |   1.552336   .4288327     1.59   0.111     .9033197    2.667658
gramnegative |   .5852059   .2408761    -1.30   0.193     .2611838    1.311206
 eitergelenk |   .6706374    .210356    -1.27   0.203     .3626561    1.240168
      strept |   1.314217   .3939959     0.91   0.362     .7302659     2.36512
anzahlopge~t |    1.55987    .111404     6.23   0.000     1.356116    1.794239
------------------------------------------------------------------------------
```

```
376 . predict p
    (option hr assumed; predicted hazard ratio)
    (177 missing values generated)

377 . roctab failure1 p

                          ROC                    —Asymptotic Normal——
              Obs        Area     Std. Err.      [95% Conf. Interval]
         ————————————————————————————————————————————————————————————
              423      0.8667       0.0219        0.82382     0.90956

378 . drop p

379 .
380 . *** OUTCOME FAILURE 2
381 . ***Gruppenvergleich
382 . tab sex failure2, chi col

    +-------------------+
    | Key               |
    |-------------------|
    |     frequency     |
    | column percentage |
    +-------------------+

               |       failure2
           sex |         0          1 |     Total
    -----------+----------------------+----------
             1 |       261        101 |       362
               |     56.99      71.13 |     60.33
    -----------+----------------------+----------
             2 |       197         41 |       238
               |     43.01      28.87 |     39.67
    -----------+----------------------+----------
         Total |       458        142 |       600
               |    100.00     100.00 |    100.00

              Pearson chi2(1) =   9.0554   Pr = 0.003

383 . gen agefailure2=age if failure2==1
    (458 missing values generated)

384 . gen agenichts=age if failure2==0
    (142 missing values generated)

385 . centile agefailure2 agenichts

                                                           —— Binom. Interp. ——
        Variable |       Obs  Percentile    Centile        [95% Conf. Interval]
    -------------+-------------------------------------------------------------
     agefailure2 |       142         50          59               56   62.18156
       agenichts |       458         50          52               50         54

386 . ranksum age, by (failure2)

    Two-sample Wilcoxon rank-sum (Mann-Whitney) test

        failure2 |      obs    rank sum    expected
    -------------+---------------------------------
               0 |      458    130195.5      137629
               1 |      142     50104.5       42671
    -------------+---------------------------------
        combined |      600      180300      180300

    unadjusted variance  3257219.67
    adjustment for ties    -1108.45
                         ----------
    adjusted variance    3256111.21
```

```
    Ho: age(failure2==0) = age(failure2==1)
                 z =  -4.119
        Prob > |z| =   0.0000

387 . gen bmifailure2=bmi if failure2==1
    (466 missing values generated)

388 . gen bminichts=bmi if failure2==0
    (227 missing values generated)

389 . centile bmifailure2 bminichts
```

Variable	Obs	Percentile	Centile	— Binom. Interp. — [95% Conf.	Interval]
bmifailure2	134	50	26.15	25.2	27.28473
bminichts	373	50	26	25.6	26.69222

```
390 . ranksum bmi, by (failure2)

    Two-sample Wilcoxon rank-sum (Mann-Whitney) test
```

failure2	obs	rank sum	expected
0	373	94199	94742
1	134	34579	34036
combined	507	128778	128778

```
    unadjusted variance  2115904.67
    adjustment for ties     -231.17
                         ----------
    adjusted variance    2115673.50

    Ho: bmi(failure2==0) = bmi(failure2==1)
                 z =  -0.373
        Prob > |z| =   0.7089

391 . gen crpfailure2=crp if failure2==1
    (487 missing values generated)

392 . gen crpnichts=crp if failure2==0
    (290 missing values generated)

393 . centile crpfailure2 crpnichts
```

Variable	Obs	Percentile	Centile	— Binom. Interp. — [95% Conf.	Interval]
crpfailure2	113	50	32.7	22.0953	46.72963
crpnichts	310	50	13.4	11.2	16.1

```
394 . ranksum crp, by (failure2)

    Two-sample Wilcoxon rank-sum (Mann-Whitney) test
```

failure2	obs	rank sum	expected
0	310	59684.5	65720
1	113	29991.5	23956
combined	423	89676	89676

```
    unadjusted variance  1237726.67
    adjustment for ties      -32.28
                         ----------
    adjusted variance    1237694.39

    Ho: crp(failure2==0) = crp(failure2==1)
                 z =  -5.425
        Prob > |z| =   0.0000
```

```
395 . gen opdauerfailure2=opdauer if failure2==1
    (459 missing values generated)

396 . gen opdauernichts=opdauer if failure2==0
    (183 missing values generated)

397 . centile opdauerfailure2 opdauernichts

                                                          ── Binom. Interp. ──
        Variable |       Obs  Percentile    Centile        [95% Conf. Interval]
    -------------+-------------------------------------------------------------
    opdauerfai~2 |       141         50    .0243056        .0208333    .0284722
    opdauernic~s |       417         50    .0166667        .0159722    .0180556

398 . ranksum opdauer, by (failure2)

    Two-sample Wilcoxon rank-sum (Mann-Whitney) test

        failure2 |      obs    rank sum    expected
    -------------+---------------------------------
               0 |      417    105949.5    116551.5
               1 |      141     50011.5     39409.5
    -------------+---------------------------------
        combined |      558      155961      155961

    unadjusted variance  2738960.25
    adjustment for ties    -1306.64
                         ----------
    adjusted variance    2737653.61

    Ho: opdauer(failure2==0) = opdauer(failure2==1)
                 z =  -6.408
        Prob > |z| =   0.0000

399 . gen anzahlopgesamtfailure2=anzahlopgesamt if failure2==1
    (458 missing values generated)

400 . gen anzahlopgesamtnichts=anzahlopgesamt if failure2==0
    (142 missing values generated)

401 . centile anzahlopgesamtfailure2 anzahlopgesamtnichts

                                                          ── Binom. Interp. ──
        Variable |       Obs  Percentile    Centile        [95% Conf. Interval]
    -------------+-------------------------------------------------------------
    anzahlopge~2 |       142         50           2               2           2
    anzahlopge~s |       458         50           1               1           1

402 . ranksum anzahlopgesamt, by (failure2)

    Two-sample Wilcoxon rank-sum (Mann-Whitney) test

        failure2 |      obs    rank sum    expected
    -------------+---------------------------------
               0 |      458    119216.5      137629
               1 |      142     61083.5       42671
    -------------+---------------------------------
        combined |      600      180300      180300

    unadjusted variance  3257219.67
    adjustment for ties   -1.20e+06
                         ----------
    adjusted variance    2058489.74

    Ho: anzahl~t(failure2==0) = anzahl~t(failure2==1)
                 z = -12.833
        Prob > |z| =   0.0000
```

403 . tab abtraeger failure2, chi col

Key
frequency
column percentage

abtraeger	failure2 0	1	Total
0	**445**<br>**97.16**	**133**<br>**93.66**	**578**<br>**96.33**
1	**13**<br>**2.84**	**9**<br>**6.34**	**22**<br>**3.67**
Total	**458**<br>**100.00**	**142**<br>**100.00**	**600**<br>**100.00**

Pearson chi2(**1**) = **3.7583** Pr = **0.053**

404 . tab sehnenresektion failure2, chi col

Key
frequency
column percentage

sehnenrese ktion	failure2 0	1	Total
1	**16**<br>**3.49**	**40**<br>**28.17**	**56**<br>**9.33**
2	**442**<br>**96.51**	**102**<br>**71.83**	**544**<br>**90.67**
Total	**458**<br>**100.00**	**142**<br>**100.00**	**600**<br>**100.00**

Pearson chi2(**1**) = **77.9924** Pr = **0.000**

405 . tab knochenresektion failure2, chi col

Key
frequency
column percentage

knochenres ektion	failure2 1	Total
1	**29**<br>**59.18**	**29**<br>**59.18**
2	**1**<br>**2.04**	**1**<br>**2.04**
3	**19**<br>**38.78**	**19**<br>**38.78**
Total	**49**<br>**100.00**	**49**<br>**100.00**

406 . tab wundheilungsstoerung failure2, chi col

Key
frequency
column percentage

wundheilun gsstoerung	failure2 0	1	Total
0	**458**<br>**100.00**	**69**<br>**48.59**	**527**<br>**87.83**
1	**0**<br>**0.00**	**73**<br>**51.41**	**73**<br>**12.17**
Total	**458**<br>**100.00**	**142**<br>**100.00**	**600**<br>**100.00**

Pearson chi2(**1**) = **268.0653** Pr = **0.000**

407 . tab aureus failure2, chi col

Key
frequency
column percentage

aureus	failure2 0	1	Total
0	**342**<br>**74.67**	**80**<br>**56.34**	**422**<br>**70.33**
1	**116**<br>**25.33**	**62**<br>**43.66**	**178**<br>**29.67**
Total	**458**<br>**100.00**	**142**<br>**100.00**	**600**<br>**100.00**

Pearson chi2(**1**) = **17.4626** Pr = **0.000**

408 . tab gramnegative failure2, chi col

Key
frequency
column percentage

gramnegati ve	failure2 0	1	Total
0	**406**<br>**88.65**	**116**<br>**81.69**	**522**<br>**87.00**
1	**52**<br>**11.35**	**26**<br>**18.31**	**78**<br>**13.00**
Total	**458**<br>**100.00**	**142**<br>**100.00**	**600**<br>**100.00**

Pearson chi2(**1**) = **4.6374** Pr = **0.031**

409 . tab strept failure2, chi col

Key
frequency   *column percentage*

strept	failure2 0	1	Total
0	**401** **87.55**	**104** **73.24**	**505** **84.17**
1	**57** **12.45**	**38** **26.76**	**95** **15.83**
Total	**458** **100.00**	**142** **100.00**	**600** **100.00**

Pearson chi2(**1**) = **16.6679** Pr = **0.000**

410 . tab diabetes failure2, chi col

Key
frequency   *column percentage*

diabetes	failure2 0	1	Total
0	**424** **92.58**	**105** **73.94**	**529** **88.17**
1	**34** **7.42**	**37** **26.06**	**71** **11.83**
Total	**458** **100.00**	**142** **100.00**	**600** **100.00**

Pearson chi2(**1**) = **36.0699** Pr = **0.000**

411 . tab eitergelenk failure2, chi col

Key
frequency   *column percentage*

eitergelen k	failure2 0	1	Total
1	**19** **4.15**	**22** **15.49**	**41** **6.83**
2	**439** **95.85**	**120** **84.51**	**559** **93.17**
Total	**458** **100.00**	**142** **100.00**	**600** **100.00**

Pearson chi2(**1**) = **21.9119** Pr = **0.000**

```
412 . tab gelenkversteifung softtissuecoverage, chi col
```

Key
frequency
column percentage

gelenkversteifung	softtissuecoverage 0	1	Total
1	8<br>1.45	1<br>2.08	9<br>1.50
2	544<br>98.55	47<br>97.92	591<br>98.50
Total	552<br>100.00	48<br>100.00	600<br>100.00

```
          Pearson chi2(1) =   0.1202   Pr = 0.729

413 . gen abfailure2=dauerabtotal if failure2==1
    (510 missing values generated)

414 . gen abnichts=dauerabtotal if failure2==0
    (236 missing values generated)

415 . centile abfailure2 abnichts
```

Variable	Obs	Percentile	Centile	Binom. Interp. [95% Conf.	Interval]
abfailure2	90	50	12	11	14.80672
abnichts	364	50	8	7	8

```
416 . ranksum dauerabtotal, by (failure2)

    Two-sample Wilcoxon rank-sum (Mann-Whitney) test
```

failure2	obs	rank sum	expected
0	364	75840	82810
1	90	27445	20475
combined	454	103285	103285

```
    unadjusted variance  1242150.00
    adjustment for ties   -17481.73
                         ----------
    adjusted variance    1224668.27

    Ho: dauera~l(failure2==0) = dauera~l(failure2==1)
                 z =  -6.298
        Prob > |z| =   0.0000

417 . tab dauerabgrp failure2, chi col
```

Key
frequency
column percentage

dauerabgrp	failure2 0	1	Total
1	170<br>37.12	20<br>14.08	190<br>31.67
2	114<br>24.89	14<br>9.86	128<br>21.33
3	174<br>37.99	108<br>76.06	282<br>47.00
Total	458<br>100.00	142<br>100.00	600<br>100.00

Pearson chi2(**2**) = **63.0567** Pr = **0.000**

418 . tab ruhigstellung failure2, chi col

Key
frequency<br>*column percentage*

ruhigstellung	failure2 0	1	Total
1	255<br>60.71	78<br>58.65	333<br>60.22
2	165<br>39.29	54<br>40.60	219<br>39.60
3	0<br>0.00	1<br>0.75	1<br>0.18
Total	420<br>100.00	133<br>100.00	553<br>100.00

Pearson chi2(**2**) = **3.2738** Pr = **0.195**

419 . tab samerecurrence failure2, chi col

Key
frequency<br>*column percentage*

samerecurrence	failure2 0	1	Total
0	458<br>100.00	139<br>97.89	597<br>99.50
1	0<br>0.00	3<br>2.11	3<br>0.50
Total	458<br>100.00	142<br>100.00	600<br>100.00

Pearson chi2(**1**) = **9.7247** Pr = **0.002**

```
420 . drop crpfailure2 crpnichts agefailure2 agenichts

421 . drop anzahlopgesamtfailure2 anzahlopgesamtnichts opdauerfailure2

422 . drop abfailure2 abnichts bmifailure2 bminichts opdauernichts

423 .
424 . ** UNIVARIATE COX Regression FAILURE 2 *
425 . stset coxend, fail (failure2) origin (dateop) id (id)

                id:  id
     failure event:  failure2 != 0 & failure2 < .
obs. time interval:  (coxend[_n-1], coxend]
 exit on or before:  failure
    t for analysis:  (time-origin)
            origin:  time dateop

------------------------------------------------------------------------------
        600  total observations
          5  ignored because never entered
------------------------------------------------------------------------------
        595  observations remaining, representing
        595  subjects
        142  failures in single-failure-per-subject data
    644,772  total analysis time at risk and under observation
                                                at risk from t =         0
                                     earliest observed entry t =         0
                                          last observed exit t =     1,427

426 . xi: stcox sex

         failure _d:  failure2
   analysis time _t:  (coxend-origin)
             origin:  time dateop
                 id:  id

Iteration 0:   log likelihood = -780.03241
Iteration 1:   log likelihood = -775.35648
Iteration 2:   log likelihood = -775.33621
Iteration 3:   log likelihood = -775.33621
Refining estimates:
Iteration 0:   log likelihood = -775.33621

Cox regression -- Breslow method for ties

No. of subjects =          595                  Number of obs    =         595
No. of failures =          142
Time at risk    =       644772
                                                LR chi2(1)       =        9.39
Log likelihood  =   -775.33621                  Prob > chi2      =      0.0022

------------------------------------------------------------------------------
          _t | Haz. Ratio   Std. Err.      z    P>|z|     [95% Conf. Interval]
-------------+----------------------------------------------------------------
         sex |   .5774538   .1070071    -2.96   0.003     .4015895    .8303326
------------------------------------------------------------------------------

427 . xi: stcox age

         failure _d:  failure2
   analysis time _t:  (coxend-origin)
             origin:  time dateop
                 id:  id

Iteration 0:   log likelihood = -780.03241
Iteration 1:   log likelihood = -772.52097
Iteration 2:   log likelihood = -772.51303
Refining estimates:
Iteration 0:   log likelihood = -772.51303

Cox regression -- Breslow method for ties
```

```
No. of subjects =          595                Number of obs    =        595
No. of failures =          142
Time at risk    =       644772
                                              LR chi2(1)       =      15.04
Log likelihood  =   -772.51303                Prob > chi2      =     0.0001
```

_t	Haz. Ratio	Std. Err.	z	P>|z|	[95% Conf.	Interval]
age	1.01776	.004693	3.82	0.000	1.008603	1.027

```
428 . xi: stcox bmi

         failure _d:  failure2
   analysis time _t:  (coxend-origin)
             origin:  time dateop
                 id:  id

Iteration 0:   log likelihood =  -716.0524
Iteration 1:   log likelihood = -715.60693
Iteration 2:   log likelihood = -715.60616
Refining estimates:
Iteration 0:   log likelihood = -715.60616

Cox regression -- Breslow method for ties

No. of subjects =          507                Number of obs    =        507
No. of failures =          134
Time at risk    =       547711
                                              LR chi2(1)       =       0.89
Log likelihood  =   -715.60616                Prob > chi2      =     0.3448
```

_t	Haz. Ratio	Std. Err.	z	P>|z|	[95% Conf.	Interval]
bmi	1.016053	.0168947	0.96	0.338	.9834737	1.049711

```
429 . xi: stcox diabetes

         failure _d:  failure2
   analysis time _t:  (coxend-origin)
             origin:  time dateop
                 id:  id

Iteration 0:   log likelihood = -780.03241
Iteration 1:   log likelihood = -771.23046
Iteration 2:   log likelihood = -764.60381
Iteration 3:   log likelihood = -764.55497
Iteration 4:   log likelihood = -764.55496
Refining estimates:
Iteration 0:   log likelihood = -764.55496

Cox regression -- Breslow method for ties

No. of subjects =          595                Number of obs    =        595
No. of failures =          142
Time at risk    =       644772
                                              LR chi2(1)       =      30.95
Log likelihood  =   -764.55496                Prob > chi2      =     0.0000
```

_t	Haz. Ratio	Std. Err.	z	P>|z|	[95% Conf.	Interval]
diabetes	3.274399	.6278735	6.19	0.000	2.248597	4.768166

```
430 . xi: stcox dauerabgrp

         failure _d:  failure2
   analysis time _t:  (coxend-origin)
             origin:  time dateop
                 id:  id

Iteration 0:   log likelihood = -780.03241
Iteration 1:   log likelihood = -755.46366
Iteration 2:   log likelihood = -754.80457
Iteration 3:   log likelihood = -754.80202
Refining estimates:
Iteration 0:   log likelihood = -754.80202

Cox regression -- Breslow method for ties

No. of subjects =          595                Number of obs    =         595
No. of failures =          142
Time at risk    =       644772
                                              LR chi2(1)       =       50.46
Log likelihood  =   -754.80202                Prob > chi2      =      0.0000
```

_t	Haz. Ratio	Std. Err.	z	P>|z|	[95% Conf.	Interval]
dauerabgrp	2.213162	.2782283	6.32	0.000	1.729833	2.831537

```
431 . xi: stcox dauerabtotal

         failure _d:  failure2
   analysis time _t:  (coxend-origin)
             origin:  time dateop
                 id:  id

Iteration 0:   log likelihood = -467.47481
Iteration 1:   log likelihood = -452.89805
Iteration 2:   log likelihood = -452.89063
Iteration 3:   log likelihood = -452.89062
Refining estimates:
Iteration 0:   log likelihood = -452.89062

Cox regression -- Breslow method for ties

No. of subjects =          449                Number of obs    =         449
No. of failures =           90
Time at risk    =       489102
                                              LR chi2(1)       =       29.17
Log likelihood  =   -452.89062                Prob > chi2      =      0.0000
```

_t	Haz. Ratio	Std. Err.	z	P>|z|	[95% Conf.	Interval]
dauerabtotal	1.040894	.0064128	6.51	0.000	1.028401	1.053539

```
432 . xi: stcox anzahlopgesamt

         failure _d:  failure2
   analysis time _t:  (coxend-origin)
             origin:  time dateop
                 id:  id

Iteration 0:   log likelihood = -780.03241
Iteration 1:   log likelihood = -747.04336
Iteration 2:   log likelihood = -745.62643
Iteration 3:   log likelihood = -745.61465
Iteration 4:   log likelihood = -745.61464
Refining estimates:
Iteration 0:   log likelihood = -745.61464
```

```
Cox regression -- Breslow method for ties

No. of subjects =          595                Number of obs    =        595
No. of failures =          142
Time at risk    =       644772
                                              LR chi2(1)       =      68.84
Log likelihood  =   -745.61464                Prob > chi2      =     0.0000
```

_t	Haz. Ratio	Std. Err.	z	P>|z|	[95% Conf.	Interval]
anzahlopge~t	1.473788	.055305	10.34	0.000	1.369282	1.58627

```
433 . xi: stcox abtraeger

         failure _d:  failure2
   analysis time _t:  (coxend-origin)
             origin:  time dateop
                 id:  id

Iteration 0:   log likelihood = -780.03241
Iteration 1:   log likelihood = -779.15036
Iteration 2:   log likelihood = -779.06303
Iteration 3:   log likelihood = -779.06275
Refining estimates:
Iteration 0:   log likelihood = -779.06275

Cox regression -- Breslow method for ties

No. of subjects =          595                Number of obs    =        595
No. of failures =          142
Time at risk    =       644772
                                              LR chi2(1)       =       1.94
Log likelihood  =   -779.06275                Prob > chi2      =     0.1637
```

_t	Haz. Ratio	Std. Err.	z	P>|z|	[95% Conf.	Interval]
abtraeger	1.67726	.5790496	1.50	0.134	.8525803	3.299631

```
434 . xi: stcox opdauer

         failure _d:  failure2
   analysis time _t:  (coxend-origin)
             origin:  time dateop
                 id:  id

Iteration 0:   log likelihood = -760.94684
Iteration 1:   log likelihood = -749.10092
Iteration 2:   log likelihood = -748.81444
Iteration 3:   log likelihood = -748.81172
Iteration 4:   log likelihood = -748.81172
Refining estimates:
Iteration 0:   log likelihood = -748.81172

Cox regression -- Breslow method for ties

No. of subjects =          558                Number of obs    =        558
No. of failures =          141
Time at risk    =       601858
                                              LR chi2(1)       =      24.27
Log likelihood  =   -748.81172                Prob > chi2      =     0.0000
```

_t	Haz. Ratio	Std. Err.	z	P>|z|	[95% Conf.	Interval]
opdauer	2262490	5554219	5.96	0.000	18406.02	2.78e+08

```
435 . xi: stcox opdauergrp

         failure _d:  failure2
   analysis time _t:  (coxend-origin)
             origin:  time dateop
                 id:  id

Iteration 0:   log likelihood = -780.03241
Iteration 1:   log likelihood = -774.11975
Iteration 2:   log likelihood = -774.04902
Iteration 3:   log likelihood = -774.04899
Refining estimates:
Iteration 0:   log likelihood = -774.04899

Cox regression -- Breslow method for ties

No. of subjects =          595                 Number of obs    =         595
No. of failures =          142
Time at risk    =       644772
                                               LR chi2(1)       =       11.97
Log likelihood  =   -774.04899                 Prob > chi2      =      0.0005

------------------------------------------------------------------------------
          _t | Haz. Ratio   Std. Err.      z    P>|z|     [95% Conf. Interval]
-------------+----------------------------------------------------------------
  opdauergrp |   1.472439   .1734748     3.28   0.001     1.168835    1.854903
------------------------------------------------------------------------------

436 . xi: stcox ruhigstellung

         failure _d:  failure2
   analysis time _t:  (coxend-origin)
             origin:  time dateop
                 id:  id

Iteration 0:   log likelihood = -716.30549
Iteration 1:   log likelihood = -714.64292
Iteration 2:   log likelihood = -714.63885
Refining estimates:
Iteration 0:   log likelihood = -714.63885

Cox regression -- Breslow method for ties

No. of subjects =          552                 Number of obs    =         552
No. of failures =          133
Time at risk    =       597322
                                               LR chi2(1)       =        3.33
Log likelihood  =   -714.63885                 Prob > chi2      =      0.0679

------------------------------------------------------------------------------
          _t | Haz. Ratio   Std. Err.      z    P>|z|     [95% Conf. Interval]
-------------+----------------------------------------------------------------
ruhigstell~g |   1.378379   .2392412     1.85   0.064     .9809079    1.936909
------------------------------------------------------------------------------

437 . xi: stcox knochenresektion

         failure _d:  failure2
   analysis time _t:  (coxend-origin)
             origin:  time dateop
                 id:  id

Iteration 0:   log likelihood = -144.81194
Iteration 1:   log likelihood = -143.87566
Iteration 2:   log likelihood = -143.87271
Refining estimates:
Iteration 0:   log likelihood = -143.87271

Cox regression -- Breslow method for ties
```

```
No. of subjects =           49                Number of obs    =          49
No. of failures =           49
Time at risk    =        52345
                                              LR chi2(1)       =        1.88
Log likelihood  =   -143.87271                Prob > chi2      =      0.1705

------------------------------------------------------------------------------
          _t | Haz. Ratio   Std. Err.      z    P>|z|     [95% Conf. Interval]
-------------+----------------------------------------------------------------
knochenres~n |   1.237448   .1899708     1.39   0.165     .9159087    1.671868
------------------------------------------------------------------------------

438 . xi: stcox eitergelenk

         failure _d:  failure2
   analysis time _t:  (coxend-origin)
             origin:  time dateop
                 id:  id

Iteration 0:   log likelihood = -780.03241
Iteration 1:   log likelihood = -775.71094
Iteration 2:   log likelihood = -773.26966
Iteration 3:   log likelihood = -773.23136
Iteration 4:   log likelihood = -773.23135
Refining estimates:
Iteration 0:   log likelihood = -773.23135

Cox regression -- Breslow method for ties

No. of subjects =          595                Number of obs    =         595
No. of failures =          142
Time at risk    =       644772
                                              LR chi2(1)       =       13.60
Log likelihood  =   -773.23135                Prob > chi2      =      0.0002

------------------------------------------------------------------------------
          _t | Haz. Ratio   Std. Err.      z    P>|z|     [95% Conf. Interval]
-------------+----------------------------------------------------------------
 eitergelenk |   .3837169   .0892248    -4.12   0.000     .2432669    .6052556
------------------------------------------------------------------------------

439 . xi: stcox crp

         failure _d:  failure2
   analysis time _t:  (coxend-origin)
             origin:  time dateop
                 id:  id

Iteration 0:   log likelihood = -588.44529
Iteration 1:   log likelihood = -581.79953
Iteration 2:   log likelihood = -577.24675
Iteration 3:   log likelihood = -577.16673
Iteration 4:   log likelihood = -577.16668
Refining estimates:
Iteration 0:   log likelihood = -577.16668

Cox regression -- Breslow method for ties

No. of subjects =          420                Number of obs    =         420
No. of failures =          113
Time at risk    =       449309
                                              LR chi2(1)       =       22.56
Log likelihood  =   -577.16668                Prob > chi2      =      0.0000

------------------------------------------------------------------------------
          _t | Haz. Ratio   Std. Err.      z    P>|z|     [95% Conf. Interval]
-------------+----------------------------------------------------------------
         crp |   1.005315   .0009972     5.34   0.000     1.003362    1.007271
------------------------------------------------------------------------------
```

```
440 . xi: stcox sehnenresektion

         failure _d:  failure2
   analysis time _t:  (coxend-origin)
             origin:  time dateop
                 id:  id

Iteration 0:   log likelihood = -780.03241
Iteration 1:   log likelihood = -769.73043
Iteration 2:   log likelihood = -763.26722
Iteration 3:   log likelihood = -763.23492
Iteration 4:   log likelihood = -763.23491
Refining estimates:
Iteration 0:   log likelihood = -763.23491

Cox regression -- Breslow method for ties

No. of subjects =          595                 Number of obs    =         595
No. of failures =          142
Time at risk    =       644772
                                               LR chi2(1)       =       33.59
Log likelihood  =   -763.23491                 Prob > chi2      =      0.0000
```

_t	Haz. Ratio	Std. Err.	z	P>|z|	[95% Conf.	Interval]
sehnenrese~n	.3003843	.0564091	-6.40	0.000	.2078884	.4340343

```
441 . xi: stcox gelenkversteifung

         failure _d:  failure2
   analysis time _t:  (coxend-origin)
             origin:  time dateop
                 id:  id

Iteration 0:   log likelihood = -780.03241
Iteration 1:   log likelihood = -774.11338
Iteration 2:   log likelihood = -774.07824
Iteration 3:   log likelihood = -774.07819
Refining estimates:
Iteration 0:   log likelihood = -774.07819

Cox regression -- Breslow method for ties

No. of subjects =          595                 Number of obs    =         595
No. of failures =          142
Time at risk    =       644772
                                               LR chi2(1)       =       11.91
Log likelihood  =   -774.07819                 Prob > chi2      =      0.0006
```

_t	Haz. Ratio	Std. Err.	z	P>|z|	[95% Conf.	Interval]
gelenkvers~g	.2349553	.081273	-4.19	0.000	.1192745	.4628316

```
442 . xi: stcox wundheilungsstoerung

         failure _d:  failure2
   analysis time _t:  (coxend-origin)
             origin:  time dateop
                 id:  id
```

```
Iteration 0:   log likelihood = -780.03241
Iteration 1:   log likelihood = -764.57338
Iteration 2:   log likelihood = -740.86936
Iteration 3:   log likelihood = -713.85872
Iteration 4:   log likelihood = -711.06156
Iteration 5:   log likelihood = -711.05861
Refining estimates:
Iteration 0:   log likelihood = -711.05861

Cox regression -- Breslow method for ties

No. of subjects =          595                  Number of obs    =         595
No. of failures =          142
Time at risk    =       644772
                                                LR chi2(1)       =      137.95
Log likelihood  =   -711.05861                  Prob > chi2      =      0.0000
```

_t	Haz. Ratio	Std. Err.	z	P>|z|	[95% Conf.	Interval]
wundheilun~g	8.3242	1.40131	12.59	0.000	5.984802	11.57805

```
443 . xi: stcox i.lappen
i.lappen          _Ilappen_0-4        (naturally coded; _Ilappen_0 omitted)

         failure _d:  failure2
   analysis time _t:  (coxend-origin)
             origin:  time dateop
                 id:  id

Iteration 0:   log likelihood = -780.03241
Iteration 1:   log likelihood = -769.18355
Iteration 2:   log likelihood = -765.15971
Iteration 3:   log likelihood = -764.53161
Iteration 4:   log likelihood = -764.52868
Refining estimates:
Iteration 0:   log likelihood = -764.52868

Cox regression -- Breslow method for ties

No. of subjects =          595                  Number of obs    =         595
No. of failures =          142
Time at risk    =       644772
                                                LR chi2(4)       =       31.01
Log likelihood  =   -764.52868                  Prob > chi2      =      0.0000
```

_t	Haz. Ratio	Std. Err.	z	P>|z|	[95% Conf.	Interval]
_Ilappen_1	3.290768	1.003678	3.91	0.000	1.810017	5.982904
_Ilappen_2	15.17457	15.37862	2.68	0.007	2.081946	110.6021
_Ilappen_3	4.095698	1.434569	4.03	0.000	2.061503	8.137142
_Ilappen_4	5.238472	2.671302	3.25	0.001	1.928163	14.23199

```
444 . xi: stcox samerecurrence

         failure _d:  failure2
   analysis time _t:  (coxend-origin)
             origin:  time dateop
                 id:  id

Iteration 0:   log likelihood = -780.03241
Iteration 1:   log likelihood = -779.54746
Iteration 2:   log likelihood = -779.27995
Iteration 3:   log likelihood =  -779.2736
Iteration 4:   log likelihood = -779.27359
Refining estimates:
Iteration 0:   log likelihood = -779.27359
```

Cox regression -- Breslow method for ties

```
No. of subjects =          595                  Number of obs    =         595
No. of failures =          142
Time at risk    =       644772
                                                LR chi2(1)       =        1.52
Log likelihood  =   -779.27359                  Prob > chi2      =      0.2180
```

_t	Haz. Ratio	Std. Err.	z	P>|z|	[95% Conf.	Interval]
samerecurr~e	2.279832	1.356522	1.39	0.166	.7102806	7.317723

445 . xi: stcox softtissuecoverage

```
         failure _d:  failure2
   analysis time _t:  (coxend-origin)
             origin:  time dateop
                 id:  id

Iteration 0:   log likelihood = -780.03241
Iteration 1:   log likelihood = -740.37518
Iteration 2:   log likelihood = -740.36999
Refining estimates:
Iteration 0:   log likelihood = -740.36999
```

Cox regression -- Breslow method for ties

```
No. of subjects =          595                  Number of obs    =         595
No. of failures =          142
Time at risk    =       644772
                                                LR chi2(1)       =       79.32
Log likelihood  =   -740.36999                  Prob > chi2      =      0.0000
```

_t	Haz. Ratio	Std. Err.	z	P>|z|	[95% Conf.	Interval]
softtissue~e	6.115975	1.086726	10.19	0.000	4.317364	8.663886

446 . xi: stcox aureus

```
         failure _d:  failure2
   analysis time _t:  (coxend-origin)
             origin:  time dateop
                 id:  id

Iteration 0:   log likelihood = -780.03241
Iteration 1:   log likelihood = -773.80582
Iteration 2:   log likelihood = -773.73635
Iteration 3:   log likelihood = -773.73635
Refining estimates:
Iteration 0:   log likelihood = -773.73635
```

Cox regression -- Breslow method for ties

```
No. of subjects =          595                  Number of obs    =         595
No. of failures =          142
Time at risk    =       644772
                                                LR chi2(1)       =       12.59
Log likelihood  =   -773.73635                  Prob > chi2      =      0.0004
```

_t	Haz. Ratio	Std. Err.	z	P>|z|	[95% Conf.	Interval]
aureus	1.847616	.3133282	3.62	0.000	1.325136	2.576101

```
447 . xi: stcox gramnegative

         failure _d:  failure2
   analysis time _t:  (coxend-origin)
             origin:  time dateop
                 id:  id

    Iteration 0:   log likelihood = -780.03241
    Iteration 1:   log likelihood =  -779.0986
    Iteration 2:   log likelihood = -779.08549
    Iteration 3:   log likelihood = -779.08549
    Refining estimates:
    Iteration 0:   log likelihood = -779.08549

    Cox regression -- Breslow method for ties

    No. of subjects =          595                 Number of obs    =          595
    No. of failures =          142
    Time at risk    =       644772
                                                   LR chi2(1)       =         1.89
    Log likelihood  =   -779.08549                 Prob > chi2      =       0.1688

    ------------------------------------------------------------------------------
              _t | Haz. Ratio   Std. Err.      z    P>|z|     [95% Conf. Interval]
    -------------+----------------------------------------------------------------
    gramnegative |   1.362994   .2968093     1.42   0.155     .8894757    2.088594
    ------------------------------------------------------------------------------

448 . xi: stcox strept

         failure _d:  failure2
   analysis time _t:  (coxend-origin)
             origin:  time dateop
                 id:  id

    Iteration 0:   log likelihood = -780.03241
    Iteration 1:   log likelihood = -777.64461
    Iteration 2:   log likelihood = -777.59879
    Iteration 3:   log likelihood = -777.59878
    Refining estimates:
    Iteration 0:   log likelihood = -777.59878

    Cox regression -- Breslow method for ties

    No. of subjects =          595                 Number of obs    =          595
    No. of failures =          142
    Time at risk    =       644772
                                                   LR chi2(1)       =         4.87
    Log likelihood  =   -777.59878                 Prob > chi2      =       0.0274

    ------------------------------------------------------------------------------
              _t | Haz. Ratio   Std. Err.      z    P>|z|     [95% Conf. Interval]
    -------------+----------------------------------------------------------------
          strept |   1.546404   .2949626     2.29   0.022     1.064055    2.247408
    ------------------------------------------------------------------------------

449 .
450 . ** MULTIVARIATE COX REGRESSION FAILURE 2**
451 . xi: stcox sex age crp bmi abvorher softtissuecoverage dauerabtotal i.dauerabg
    > rp abtraeger diabetes aureus gramnegative eitergelenk strept anzahlopgesamt
    i.dauerabgrp      _Idauerabgr_1-3     (naturally coded; _Idauerabgr_1 omitted)

         failure _d:  failure2
   analysis time _t:  (coxend-origin)
             origin:  time dateop
                 id:  id
```

```
Iteration 0:   log likelihood = -330.84093
Iteration 1:   log likelihood = -290.32312
Iteration 2:   log likelihood = -282.30609
Iteration 3:   log likelihood = -281.93809
Iteration 4:   log likelihood = -281.93709
Iteration 5:   log likelihood = -281.93709
Refining estimates:
Iteration 0:   log likelihood = -281.93709

Cox regression -- Breslow method for ties

No. of subjects =          268                Number of obs    =         268
No. of failures =           69
Time at risk    =       285770
                                              LR chi2(16)      =       97.81
Log likelihood  =   -281.93709                Prob > chi2      =      0.0000
```

_t	Haz. Ratio	Std. Err.	z	P>|z|	[95% Conf.	Interval]
sex	.4137733	.1450464	-2.52	0.012	.2081505	.8225218
age	1.012984	.0088648	1.47	0.140	.9957574	1.030508
crp	.9993521	.0018813	-0.34	0.731	.9956716	1.003046
bmi	.9996612	.0234856	-0.01	0.988	.9546739	1.046768
abvorher	1.539683	.6287457	1.06	0.291	.6915672	3.427902
softtissue~e	2.584447	1.117465	2.20	0.028	1.107451	6.031297
dauerabtotal	.9834193	.0139074	-1.18	0.237	.9565355	1.011059
_Idauerabg~2	.6436655	.3089877	-0.92	0.359	.2512154	1.649204
_Idauerabg~3	3.158208	1.179539	3.08	0.002	1.518906	6.566749
abtraeger	.7442984	.4390298	-0.50	0.617	.2342393	2.365018
diabetes	1.926536	.6468265	1.95	0.051	.9976764	3.720185
aureus	.763129	.2595038	-0.79	0.427	.3918725	1.48611
gramnegative	2.122135	.8546575	1.87	0.062	.9637504	4.672846
eitergelenk	1.362854	.7181671	0.59	0.557	.4851788	3.828221
strept	.9081452	.3094812	-0.28	0.777	.4656714	1.771051
anzahlopge~t	1.729274	.2968413	3.19	0.001	1.235234	2.420907

```
452 . predict p
    (option hr assumed; predicted hazard ratio)
    (332 missing values generated)

453 . roctab failure2 p
```

Obs	ROC Area	Std. Err.	—Asymptotic Normal— [95% Conf.	Interval]
268	0.8702	0.0266	0.81799	0.92245

```
454 . drop p

455 .
456 . *** OUTCOME SAME RECURRENCE MIT GLEICHEM KEIM
457 . ***Gruppenvergleich
458 . tab sex samerecurrence, chi col
```

Key
frequency<br>*column percentage*

sex	samerecurrence 0	1	Total
1	360<br>60.30	2<br>66.67	362<br>60.33
2	237<br>39.70	1<br>33.33	238<br>39.67
Total	597<br>100.00	3<br>100.00	600<br>100.00

Pearson chi2(**1**) = **0.0505** Pr = **0.822**

```
459 . gen agesamerecurrence=age if samerecurrence==1
    (597 missing values generated)

460 . gen agenichts=age if samerecurrence==0
    (3 missing values generated)

461 . centile agesamerecurrence agenichts
```

Variable	Obs	Percentile	Centile	— Binom. Interp. — [95% Conf.	Interval]
agesamerec~e	3	50	69	29	70*
agenichts	597	50	54	52	55

* Lower (upper) confidence limit held at minimum (maximum) of sample

```
462 . ranksum age, by (samerecurrence)
```

Two-sample Wilcoxon rank-sum (Mann-Whitney) test

samerecurr~e	obs	rank sum	expected
0	597	179264.5	179398.5
1	3	1035.5	901.5
combined	600	180300	180300

```
unadjusted variance    89699.25
adjustment for ties      -30.53
                      ----------
adjusted variance      89668.72

Ho: age(samere~e==0) = age(samere~e==1)
             z =  -0.447
    Prob > |z| =   0.6545
```

```
463 . gen bmisamerecurrence=bmi if samerecurrence==1
    (597 missing values generated)

464 . gen bminichts=bmi if samerecurrence==0
    (96 missing values generated)

465 . centile bmisamerecurrence bminichts
```

Variable	Obs	Percentile	Centile	— Binom. Interp. — [95% Conf.	Interval]
bmisamerec~e	3	50	25.4	24.2	32.4*
bminichts	504	50	26	25.6488	26.6

* Lower (upper) confidence limit held at minimum (maximum) of sample

```
466 . ranksum bmi, by (samerecurrence)

    Two-sample Wilcoxon rank-sum (Mann-Whitney) test

    samerecurr~e |      obs    rank sum    expected
    -------------+---------------------------------
               0 |      504    127948.5      128016
               1 |        3       829.5         762
    -------------+---------------------------------
        combined |      507      128778      128778

    unadjusted variance    64008.00
    adjustment for ties       -6.99
                          ----------
    adjusted variance      64001.01

    Ho: bmi(samere~e==0) = bmi(samere~e==1)
                 z =  -0.267
        Prob > |z| =   0.7896

467 . gen crpsamerecurrence=crp if samerecurrence==1
    (599 missing values generated)

468 . gen crpnichts=crp if samerecurrence==0
    (178 missing values generated)

469 . centile crpsamerecurrence crpnichts

                                                          -- Binom. Interp. --
        Variable |       Obs  Percentile    Centile        [95% Conf. Interval]
    -------------+-------------------------------------------------------------
    crpsamerec~e |         1          50       20.5             20.5        20.5*
       crpnichts |       422          50       16.6             13.9        18.9

     Lower (upper) confidence limit held at minimum (maximum) of sample

470 . ranksum crp, by (samerecurrence)

    Two-sample Wilcoxon rank-sum (Mann-Whitney) test

    samerecurr~e |      obs    rank sum    expected
    -------------+---------------------------------
               0 |      422       89427       89464
               1 |        1         249         212
    -------------+---------------------------------
        combined |      423       89676       89676

    unadjusted variance    14910.67
    adjustment for ties       -0.39
                          ----------
    adjusted variance      14910.28

    Ho: crp(samere~e==0) = crp(samere~e==1)
                 z =  -0.303
        Prob > |z| =   0.7619

471 . gen opdauersamerecurrence=opdauer if samerecurrence==1
    (597 missing values generated)

472 . gen opdauernichts=opdauer if samerecurrence==0
    (45 missing values generated)
```

```
473 . centile opdauersamerecurrence opdauernichts

                                                       -- Binom. Interp. --
    Variable |       Obs  Percentile    Centile        [95% Conf. Interval]
-------------+-------------------------------------------------------------
opdauersam~e |         3         50    .0194444        .0118056    .0340278*
opdauernic~s |       555         50      .01875        .0173611    .0201389

 Lower (upper) confidence limit held at minimum (maximum) of sample

474 . ranksum opdauer, by (samerecurrence)

Two-sample Wilcoxon rank-sum (Mann-Whitney) test

samerecurr~e |      obs    rank sum    expected
-------------+---------------------------------
           0 |      555    155082.5    155122.5
           1 |        3       878.5       838.5
-------------+---------------------------------
    combined |      558      155961      155961

unadjusted variance    77561.25
adjustment for ties      -37.00
                     ----------
adjusted variance      77524.25

Ho: opdauer(samere~e==0) = opdauer(samere~e==1)
             z =  -0.144
    Prob > |z| =   0.8858

475 . gen anzahlopgesamtsamerecurrence=anzahlopgesamt if samerecurrence==1
    (597 missing values generated)

476 . gen anzahlopgesamtnichts=anzahlopgesamt if samerecurrence==0
    (3 missing values generated)

477 . centile anzahlopgesamtsamerecurrence anzahlopgesamtnichts

                                                       -- Binom. Interp. --
    Variable |       Obs  Percentile    Centile        [95% Conf. Interval]
-------------+-------------------------------------------------------------
anzahlopge~e |         3         50           3               2           4*
anzahlopge~s |       597         50           1               1           1

 Lower (upper) confidence limit held at minimum (maximum) of sample

478 . ranksum anzahlopgesamt, by (samerecurrence)

Two-sample Wilcoxon rank-sum (Mann-Whitney) test

samerecurr~e |      obs    rank sum    expected
-------------+---------------------------------
           0 |      597    178690.5    179398.5
           1 |        3      1609.5       901.5
-------------+---------------------------------
    combined |      600      180300      180300

unadjusted variance    89699.25
adjustment for ties   -33011.34
                     ----------
adjusted variance      56687.91

Ho: anzahl~t(samere~e==0) = anzahl~t(samere~e==1)
             z =  -2.974
    Prob > |z| =   0.0029
```

479 . tab abtraeger samerecurrence, chi col

Key
frequency<br>*column percentage*

abtraeger	samerecurrence 0	1	Total
0	**575**<br>**96.31**	**3**<br>**100.00**	**578**<br>**96.33**
1	**22**<br>**3.69**	**0**<br>**0.00**	**22**<br>**3.67**
Total	**597**<br>**100.00**	**3**<br>**100.00**	**600**<br>**100.00**

Pearson chi2(**1**) = **0.1148** Pr = **0.735**

480 . tab sehnenresektion samerecurrence, chi col

Key
frequency<br>*column percentage*

sehnenrese ktion	samerecurrence 0	1	Total
1	**56**<br>**9.38**	**0**<br>**0.00**	**56**<br>**9.33**
2	**541**<br>**90.62**	**3**<br>**100.00**	**544**<br>**90.67**
Total	**597**<br>**100.00**	**3**<br>**100.00**	**600**<br>**100.00**

Pearson chi2(**1**) = **0.3104** Pr = **0.577**

481 . tab knochenresektion samerecurrence, chi col

Key
frequency<br>*column percentage*

knochenres ektion	samerecurrence 0	1	Total
1	**29**<br>**60.42**	**0**<br>**0.00**	**29**<br>**59.18**
2	**1**<br>**2.08**	**0**<br>**0.00**	**1**<br>**2.04**
3	**18**<br>**37.50**	**1**<br>**100.00**	**19**<br>**38.78**
Total	**48**<br>**100.00**	**1**<br>**100.00**	**49**<br>**100.00**

Pearson chi2(**2**) = **1.6118** Pr = **0.447**

482 . tab wundheilungsstoerung samerecurrence, chi col

Key
frequency<br>*column percentage*

wundheilun gsstoerung	samerecurrence 0	1	Total
0	**525**<br>**87.94**	**2**<br>**66.67**	**527**<br>**87.83**
1	**72**<br>**12.06**	**1**<br>**33.33**	**73**<br>**12.17**
Total	**597**<br>**100.00**	**3**<br>**100.00**	**600**<br>**100.00**

Pearson chi2(**1**) = **1.2641** Pr = **0.261**

483 . tab aureus samerecurrence, chi col

Key
frequency<br>*column percentage*

aureus	samerecurrence 0	1	Total
0	**421**<br>**70.52**	**1**<br>**33.33**	**422**<br>**70.33**
1	**176**<br>**29.48**	**2**<br>**66.67**	**178**<br>**29.67**
Total	**597**<br>**100.00**	**3**<br>**100.00**	**600**<br>**100.00**

Pearson chi2(**1**) = **1.9782** Pr = **0.160**

484 . tab gramnegative samerecurrence, chi col

Key
frequency<br>*column percentage*

gramnegati ve	samerecurrence 0	1	Total
0	**519**<br>**86.93**	**3**<br>**100.00**	**522**<br>**87.00**
1	**78**<br>**13.07**	**0**<br>**0.00**	**78**<br>**13.00**
Total	**597**<br>**100.00**	**3**<br>**100.00**	**600**<br>**100.00**

Pearson chi2(**1**) = **0.4505** Pr = **0.502**

485 . tab strept samerecurrence, chi col

Key
frequency<br>*column percentage*

strept	samerecurrence 0	1	Total
0	503<br>84.25	2<br>66.67	505<br>84.17
1	94<br>15.75	1<br>33.33	95<br>15.83
Total	597<br>100.00	3<br>100.00	600<br>100.00

Pearson chi2(**1**) = **0.6929** Pr = **0.405**

486 . tab diabetes samerecurrence, chi col

Key
frequency<br>*column percentage*

diabetes	samerecurrence 0	1	Total
0	527<br>88.27	2<br>66.67	529<br>88.17
1	70<br>11.73	1<br>33.33	71<br>11.83
Total	597<br>100.00	3<br>100.00	600<br>100.00

Pearson chi2(**1**) = **1.3359** Pr = **0.248**

487 . tab eitergelenk samerecurrence, chi col

Key
frequency<br>*column percentage*

eitergelen k	samerecurrence 0	1	Total
1	39<br>6.53	2<br>66.67	41<br>6.83
2	558<br>93.47	1<br>33.33	559<br>93.17
Total	597<br>100.00	3<br>100.00	600<br>100.00

Pearson chi2(**1**) = **16.9548** Pr = **0.000**

```
488 . tab gelenkversteifung softtissuecoverage, chi col

  +-------------------+
  | Key               |
  |-------------------|
  |     frequency     |
  | column percentage |
  +-------------------+

gelenkvers |  softtissuecoverage
   teifung |         0          1 |     Total
-----------+----------------------+----------
         1 |         8          1 |         9 
           |      1.45       2.08 |      1.50 
-----------+----------------------+----------
         2 |       544         47 |       591 
           |     98.55      97.92 |     98.50 
-----------+----------------------+----------
     Total |       552         48 |       600 
           |    100.00     100.00 |    100.00 

          Pearson chi2(1) =   0.1202   Pr = 0.729

489 . gen absamerecurrence=dauerabtotal if samerecurrence==1
    (599 missing values generated)

490 . gen abnichts=dauerabtotal if samerecurrence==0
    (147 missing values generated)

491 . centile absamerecurrence abnichts

                                                       -- Binom. Interp. --
    Variable |       Obs  Percentile    Centile        [95% Conf. Interval]
-------------+-------------------------------------------------------------
absamerecu~e |         1         50          20              20          20*
    abnichts |       453         50           8               8           9

 Lower (upper) confidence limit held at minimum (maximum) of sample

492 . ranksum dauerabtotal, by (samerecurrence)

    Two-sample Wilcoxon rank-sum (Mann-Whitney) test

samerecurr~e |      obs    rank sum    expected
-------------+---------------------------------
           0 |      453    102874.5    103057.5
           1 |        1       410.5       227.5
-------------+---------------------------------
    combined |      454      103285      103285

unadjusted variance    17176.25
adjustment for ties     -241.73
                     ----------
adjusted variance      16934.52

Ho: dauera~l(samere~e==0) = dauera~l(samere~e==1)
             z =  -1.406
    Prob > |z| =   0.1596

493 . tab dauerabgrp samerecurrence, chi col

  +-------------------+
  | Key               |
  |-------------------|
  |     frequency     |
  | column percentage |
  +-------------------+
```

dauerabgrp	samerecurrence 0	1	Total
1	**190**<br>**31.83**	**0**<br>**0.00**	**190**<br>**31.67**
2	**128**<br>**21.44**	**0**<br>**0.00**	**128**<br>**21.33**
3	**279**<br>**46.73**	**3**<br>**100.00**	**282**<br>**47.00**
Total	**597**<br>**100.00**	**3**<br>**100.00**	**600**<br>**100.00**

Pearson chi2(**2**) = **3.4000** Pr = **0.183**

```
494 . tab ruhigstellung samerecurrence, chi col
```

Key
frequency<br>*column percentage*

ruhigstellung	samerecurrence 0	1	Total
1	**332**<br>**60.36**	**1**<br>**33.33**	**333**<br>**60.22**
2	**217**<br>**39.45**	**2**<br>**66.67**	**219**<br>**39.60**
3	**1**<br>**0.18**	**0**<br>**0.00**	**1**<br>**0.18**
Total	**550**<br>**100.00**	**3**<br>**100.00**	**553**<br>**100.00**

Pearson chi2(**2**) = **0.9254** Pr = **0.630**

```
495 . drop crpsamerecurrence crpnichts agesamerecurrence agenichts

496 . drop anzahlopgesamtsamerecurrence anzahlopgesamtnichts opdauersamerecurrence

497 . drop absamerecurrence abnichts bmisamerecurrence bminichts opdauernichts

498 .
499 . ** UNIVARIATE COX Regression SAMERECURRENCE *
500 . stset coxend, fail (samerecurrence) origin (dateop) id (id)

                id:  id
     failure event:  samerecurrence != 0 & samerecurrence < .
obs. time interval:  (coxend[_n-1], coxend]
 exit on or before:  failure
    t for analysis:  (time-origin)
            origin:  time dateop

------------------------------------------------------------------------------
        600  total observations
          5  ignored because never entered
------------------------------------------------------------------------------
        595  observations remaining, representing
        595  subjects
          3  failures in single-failure-per-subject data
    644,772  total analysis time at risk and under observation
                                                at risk from t =         0
                                     earliest observed entry t =         0
                                          last observed exit t =     1,427
```

```
501 . xi: stcox sex

         failure _d:  samerecurrence
   analysis time _t:  (coxend-origin)
             origin:  time dateop
                 id:  id

Iteration 0:   log likelihood = -13.871301
Iteration 1:   log likelihood = -13.827779
Iteration 2:   log likelihood = -13.827716
Refining estimates:
Iteration 0:   log likelihood = -13.827716

Cox regression -- no ties

No. of subjects =          595                  Number of obs    =         595
No. of failures =            3
Time at risk    =       644772
                                                LR chi2(1)       =        0.09
Log likelihood  =   -13.827716                  Prob > chi2      =      0.7678

------------------------------------------------------------------------------
          _t | Haz. Ratio   Std. Err.      z    P>|z|     [95% Conf. Interval]
-------------+----------------------------------------------------------------
         sex |   .7005245   .8593174    -0.29   0.772     .0632811     7.75484
------------------------------------------------------------------------------

502 . xi: stcox age

         failure _d:  samerecurrence
   analysis time _t:  (coxend-origin)
             origin:  time dateop
                 id:  id

Iteration 0:   log likelihood = -13.871301
Iteration 1:   log likelihood = -13.709965
Iteration 2:   log likelihood = -13.709877
Refining estimates:
Iteration 0:   log likelihood = -13.709877

Cox regression -- no ties

No. of subjects =          595                  Number of obs    =         595
No. of failures =            3
Time at risk    =       644772
                                                LR chi2(1)       =        0.32
Log likelihood  =   -13.709877                  Prob > chi2      =      0.5699

------------------------------------------------------------------------------
          _t | Haz. Ratio   Std. Err.      z    P>|z|     [95% Conf. Interval]
-------------+----------------------------------------------------------------
         age |   1.018125   .0326188     0.56   0.575      .956159    1.084107
------------------------------------------------------------------------------

503 . xi: stcox bmi

         failure _d:  samerecurrence
   analysis time _t:  (coxend-origin)
             origin:  time dateop
                 id:  id

Iteration 0:   log likelihood = -13.067263
Iteration 1:   log likelihood = -13.017176
Iteration 2:   log likelihood = -13.016621
Iteration 3:   log likelihood = -13.016621
Refining estimates:
Iteration 0:   log likelihood = -13.016621

Cox regression -- no ties
```

```
No. of subjects =          507                  Number of obs    =        507
No. of failures =            3
Time at risk    =       547711
                                                LR chi2(1)       =       0.10
Log likelihood  =   -13.016621                  Prob > chi2      =     0.7503

------------------------------------------------------------------------------
          _t | Haz. Ratio   Std. Err.      z    P>|z|     [95% Conf. Interval]
-------------+----------------------------------------------------------------
         bmi |   1.037195   .1151073     0.33   0.742      .834439    1.289218
------------------------------------------------------------------------------
```

504 . xi: stcox diabetes

```
         failure _d:  samerecurrence
   analysis time _t:  (coxend-origin)
             origin:  time dateop
                 id:  id

Iteration 0:   log likelihood = -13.871301
Iteration 1:   log likelihood = -13.684957
Iteration 2:   log likelihood = -13.265514
Iteration 3:   log likelihood = -13.265496
Refining estimates:
Iteration 0:   log likelihood = -13.265496

Cox regression -- no ties

No. of subjects =          595                  Number of obs    =        595
No. of failures =            3
Time at risk    =       644772
                                                LR chi2(1)       =       1.21
Log likelihood  =   -13.265496                  Prob > chi2      =     0.2710

------------------------------------------------------------------------------
          _t | Haz. Ratio   Std. Err.      z    P>|z|     [95% Conf. Interval]
-------------+----------------------------------------------------------------
    diabetes |   4.533417     5.5756     1.23   0.219     .4069511    50.50206
------------------------------------------------------------------------------
```

505 . xi: stcox dauerabgrp

```
         failure _d:  samerecurrence
   analysis time _t:  (coxend-origin)
             origin:  time dateop
                 id:  id

Iteration 0:   log likelihood = -13.871301
Iteration 1:   log likelihood = -11.850194
Iteration 2:   log likelihood = -11.536119
Iteration 3:   log likelihood =  -11.41679
Iteration 4:   log likelihood = -11.366456
Iteration 5:   log likelihood = -11.345097
Iteration 6:   log likelihood = -11.336403
Iteration 7:   log likelihood = -11.333027
Iteration 8:   log likelihood = -11.331756
Iteration 9:   log likelihood = -11.331284
Iteration 10:  log likelihood = -11.331109
Iteration 11:  log likelihood = -11.331045
Iteration 12:  log likelihood = -11.331022
Iteration 13:  log likelihood = -11.331013
Iteration 14:  log likelihood =  -11.33101
Iteration 15:  log likelihood = -11.331009
Iteration 16:  log likelihood = -11.331008
Iteration 17:  log likelihood = -11.331008
Iteration 18:  log likelihood = -11.331008
Iteration 19:  log likelihood = -11.331008
Iteration 20:  log likelihood = -11.331008
Iteration 21:  log likelihood = -11.331008
Iteration 22:  log likelihood = -11.331008
Iteration 23:  log likelihood = -11.331008
Iteration 24:  log likelihood = -11.331008
```

```
Iteration 25:  log likelihood = -11.331008
Iteration 26:  log likelihood = -11.331008
Iteration 27:  log likelihood = -11.331008
Iteration 28:  log likelihood = -11.331008
Iteration 29:  log likelihood = -11.331008
Iteration 30:  log likelihood = -11.331008
Iteration 31:  log likelihood = -11.331008
Refining estimates:
Iteration 0:   log likelihood = -11.331008
Iteration 1:   log likelihood = -11.331008
Iteration 2:   log likelihood = -11.331008
Iteration 3:   log likelihood = -11.331008
Iteration 4:   log likelihood = -11.331008

Cox regression -- no ties

No. of subjects =          595                 Number of obs    =          595
No. of failures =            3
Time at risk    =       644772
                                               LR chi2(1)       =         5.08
Log likelihood  =   -11.331008                 Prob > chi2      =       0.0242
```

_t	Haz. Ratio	Std. Err.	z	P>|z|	[95% Conf.	Interval]
dauerabgrp	1.75e+14	2.93e+21	0.00	1.000	0	.

```
506 . xi: stcox dauerabtotal

         failure _d:  samerecurrence
   analysis time _t:  (coxend-origin)
             origin:  time dateop
                 id:  id

Iteration 0:   log likelihood = -2.0794415
Iteration 1:   log likelihood = -.48139426
Iteration 2:   log likelihood = -.24783418
Iteration 3:   log likelihood = -.19156565
Iteration 4:   log likelihood = -.18701518
Refining estimates:
Iteration 0:   log likelihood = -.17967727
Iteration 1:   log likelihood = -.07297315
Iteration 2:   log likelihood = -.02779638
Iteration 3:   log likelihood = -.01199657

Cox regression -- no ties

No. of subjects =          449                 Number of obs    =          449
No. of failures =            1
Time at risk    =       489102
                                               LR chi2(0)       =         4.13
Log likelihood  =   -.01199657                 Prob > chi2      =            .
```

_t	Haz. Ratio	Std. Err.	z	P>|z|	[95% Conf.	Interval]
dauerabtotal	3.658575	.	.	.	.	.

```
507 . xi: stcox anzahlopgesamt

         failure _d:  samerecurrence
   analysis time _t:  (coxend-origin)
             origin:  time dateop
                 id:  id

Iteration 0:   log likelihood = -13.871301
Iteration 1:   log likelihood = -12.860466
Iteration 2:   log likelihood = -12.138327
Iteration 3:   log likelihood = -12.138263
Refining estimates:
Iteration 0:   log likelihood = -12.138263

Cox regression -- no ties

No. of subjects =          595                  Number of obs    =        595
No. of failures =            3
Time at risk    =       644772
                                                LR chi2(1)       =       3.47
Log likelihood  =   -12.138263                  Prob > chi2      =     0.0626
```

_t	Haz. Ratio	Std. Err.	z	P>|z|	[95% Conf.	Interval]
anzahlopge~t	1.753217	.4106884	2.40	0.017	1.107753	2.774779

```
508 . xi: stcox abtraeger

         failure _d:  samerecurrence
   analysis time _t:  (coxend-origin)
             origin:  time dateop
                 id:  id

Iteration 0:   log likelihood = -13.871301
Iteration 1:   log likelihood = -13.824719
Iteration 2:   log likelihood = -13.808164
Iteration 3:   log likelihood = -13.802149
Iteration 4:   log likelihood = -13.799945
Iteration 5:   log likelihood = -13.799136
Iteration 6:   log likelihood = -13.798839
Iteration 7:   log likelihood = -13.798729
Iteration 8:   log likelihood = -13.798689
Iteration 9:   log likelihood = -13.798674
Iteration 10:  log likelihood = -13.798669
Iteration 11:  log likelihood = -13.798667
Iteration 12:  log likelihood = -13.798666
Iteration 13:  log likelihood = -13.798666
Iteration 14:  log likelihood = -13.798666
Iteration 15:  log likelihood = -13.798665
Iteration 16:  log likelihood = -13.798665
Iteration 17:  log likelihood = -13.798665
Iteration 18:  log likelihood = -13.798665
Iteration 19:  log likelihood = -13.798665
Iteration 20:  log likelihood = -13.798665
Iteration 21:  log likelihood = -13.798665
Iteration 22:  log likelihood = -13.798665
Iteration 23:  log likelihood = -13.798665
Iteration 24:  log likelihood = -13.798665
Iteration 25:  log likelihood = -13.798665
Iteration 26:  log likelihood = -13.798665
Iteration 27:  log likelihood = -13.798665
Iteration 28:  log likelihood = -13.798665
Iteration 29:  log likelihood = -13.798665
Iteration 30:  log likelihood = -13.798665
Iteration 31:  log likelihood = -13.798665
Iteration 32:  log likelihood = -13.798665
Iteration 33:  log likelihood = -13.798665
Iteration 34:  log likelihood = -13.798665
Iteration 35:  log likelihood = -13.798665
Refining estimates:
```

```
Iteration 0:   log likelihood = -13.798665
Iteration 1:   log likelihood = -13.798665

Cox regression -- no ties

No. of subjects =          595                Number of obs    =          595
No. of failures =            3
Time at risk    =       644772
                                              LR chi2(1)       =         0.15
Log likelihood  =   -13.798665                Prob > chi2      =       0.7031
```

_t	Haz. Ratio	Std. Err.	z	P>|z|	[95% Conf.	Interval]
abtraeger	2.19e-16	5.44e-08	-0.00	1.000	0	.

509 . xi: stcox opdauer

```
         failure _d:  samerecurrence
   analysis time _t:  (coxend-origin)
             origin:  time dateop
                 id:  id

Iteration 0:   log likelihood = -13.237957
Iteration 1:   log likelihood = -13.232148
Iteration 2:   log likelihood = -13.232095
Iteration 3:   log likelihood = -13.232095
Refining estimates:
Iteration 0:   log likelihood = -13.232095

Cox regression -- no ties

No. of subjects =          558                Number of obs    =          558
No. of failures =            3
Time at risk    =       601858
                                              LR chi2(1)       =         0.01
Log likelihood  =   -13.232095                Prob > chi2      =       0.9138
```

_t	Haz. Ratio	Std. Err.	z	P>|z|	[95% Conf.	Interval]
opdauer	.024371	.8637244	-0.10	0.917	1.66e-32	3.58e+28

510 . xi: stcox opdauergrp

```
         failure _d:  samerecurrence
   analysis time _t:  (coxend-origin)
             origin:  time dateop
                 id:  id

Iteration 0:   log likelihood = -13.871301
Iteration 1:   log likelihood = -13.870168
Iteration 2:   log likelihood = -13.870168
Refining estimates:
Iteration 0:   log likelihood = -13.870168

Cox regression -- no ties

No. of subjects =          595                Number of obs    =          595
No. of failures =            3
Time at risk    =       644772
                                              LR chi2(1)       =         0.00
Log likelihood  =   -13.870168                Prob > chi2      =       0.9620
```

_t	Haz. Ratio	Std. Err.	z	P>|z|	[95% Conf.	Interval]
opdauergrp	1.033499	.7176997	0.05	0.962	.2649746	4.031027

```
511 . xi: stcox i.ruhigstellung
    i.ruhigstellung   _Iruhigstel_1-3     (naturally coded; _Iruhigstel_1 omitted)

             failure _d:  samerecurrence
       analysis time _t:  (coxend-origin)
                 origin:  time dateop
                     id:  id

    Iteration 0:   log likelihood =  -13.23826
    Iteration 1:   log likelihood = -12.678338
    Iteration 2:   log likelihood = -12.676194
    Iteration 3:   log likelihood = -12.675777
    Iteration 4:   log likelihood = -12.675624
    Iteration 5:   log likelihood = -12.675568
    Iteration 6:   log likelihood = -12.675547
    Iteration 7:   log likelihood =  -12.67554
    Iteration 8:   log likelihood = -12.675537
    Iteration 9:   log likelihood = -12.675536
    Iteration 10:  log likelihood = -12.675536
    Iteration 11:  log likelihood = -12.675535
    Iteration 12:  log likelihood = -12.675535
    Iteration 13:  log likelihood = -12.675535
    Iteration 14:  log likelihood = -12.675535
    Iteration 15:  log likelihood = -12.675535
    Iteration 16:  log likelihood = -12.675535
    Iteration 17:  log likelihood = -12.675535
    Iteration 18:  log likelihood = -12.675535
    Iteration 19:  log likelihood = -12.675535
    Iteration 20:  log likelihood = -12.675535
    Iteration 21:  log likelihood = -12.675535
    Iteration 22:  log likelihood = -12.675535
    Iteration 23:  log likelihood = -12.675535
    Iteration 24:  log likelihood = -12.675535
    Iteration 25:  log likelihood = -12.675535
    Iteration 26:  log likelihood = -12.675535
    Iteration 27:  log likelihood = -12.675535
    Iteration 28:  log likelihood = -12.675535
    Iteration 29:  log likelihood = -12.675535
    Iteration 30:  log likelihood = -12.675535
    Iteration 31:  log likelihood = -12.675535
    Iteration 32:  log likelihood = -12.675535
    Iteration 33:  log likelihood = -12.675535
    Iteration 34:  log likelihood = -12.675535
    Iteration 35:  log likelihood = -12.675535
    Iteration 36:  log likelihood = -12.675535
    Iteration 37:  log likelihood = -12.675535
    Iteration 38:  log likelihood = -12.675535
    Iteration 39:  log likelihood = -12.675535
    Refining estimates:
    Iteration 0:   log likelihood = -12.675535

    Cox regression -- no ties

    No. of subjects =          552                 Number of obs    =         552
    No. of failures =            3
    Time at risk    =       597322
                                                   LR chi2(1)       =        1.13
    Log likelihood  =   -12.675535                 Prob > chi2      =      0.2887
```

_t	Haz. Ratio	Std. Err.	z	P>|z|	[95% Conf.	Interval]
_Iruhigste~2	3.471425	4.256682	1.01	0.310	.3138769	38.39337
_Iruhigste~3	1.74e-17	.	.	.	.	.

```
512 . xi: stcox knochenresektion

          failure _d:  samerecurrence
    analysis time _t:  (coxend-origin)
              origin:  time dateop
                  id:  id

    Iteration 0:   log likelihood = -3.2580965
    Iteration 1:   log likelihood = -2.4148755
    Iteration 2:   log likelihood =   -2.34067
    Iteration 3:   log likelihood = -2.3162287
    Iteration 4:   log likelihood = -2.3075574
    Iteration 5:   log likelihood = -2.3044081
    Iteration 6:   log likelihood = -2.3032549
    Iteration 7:   log likelihood = -2.3028314
    Iteration 8:   log likelihood = -2.3026757
    Iteration 9:   log likelihood = -2.3026184
    Iteration 10:  log likelihood = -2.3025974
    Iteration 11:  log likelihood = -2.3025896
    Iteration 12:  log likelihood = -2.3025868
    Iteration 13:  log likelihood = -2.3025857
    Iteration 14:  log likelihood = -2.3025853
    Iteration 15:  log likelihood = -2.3025852
    Iteration 16:  log likelihood = -2.3025851
    Iteration 17:  log likelihood = -2.3025851
    Iteration 18:  log likelihood = -2.3025851
    Iteration 19:  log likelihood = -2.3025851
    Iteration 20:  log likelihood = -2.3025851
    Iteration 21:  log likelihood = -2.3025851
    Iteration 22:  log likelihood = -2.3025851
    Iteration 23:  log likelihood = -2.3025851
    Iteration 24:  log likelihood = -2.3025851
    Iteration 25:  log likelihood = -2.3025851
    Iteration 26:  log likelihood = -2.3025851
    Iteration 27:  log likelihood = -2.3025851
    Iteration 28:  log likelihood = -2.3025851
    Iteration 29:  log likelihood = -2.3025851
    Iteration 30:  log likelihood = -2.3025851
    Iteration 31:  log likelihood = -2.3025851
    Iteration 32:  log likelihood = -2.3025851
    Iteration 33:  log likelihood = -2.3025851
    Refining estimates:
    Iteration 0:   log likelihood = -2.3025851

    Cox regression -- no ties

    No. of subjects =           49                  Number of obs    =         49
    No. of failures =            1
    Time at risk    =        52345
                                                    LR chi2(0)       =       1.91
    Log likelihood  =   -2.3025851                  Prob > chi2      =          .

    ------------------------------------------------------------------------------
              _t | Haz. Ratio   Std. Err.      z    P>|z|     [95% Conf. Interval]
    -------------+----------------------------------------------------------------
    knochenres~n |   8.66e+07          .       .       .             .           .
    ------------------------------------------------------------------------------

513 . xi: stcox eitergelenk

          failure _d:  samerecurrence
    analysis time _t:  (coxend-origin)
              origin:  time dateop
                  id:  id
```

```
Iteration 0:   log likelihood = -13.871301
Iteration 1:   log likelihood = -8.1196967
Refining estimates:
Iteration 0:   log likelihood = -8.1196969
Iteration 1:   log likelihood = -8.1196965
Iteration 2:   log likelihood = -8.1196963
Iteration 3:   log likelihood = -8.1196963
Iteration 4:   log likelihood = -8.1196963
Iteration 5:   log likelihood = -8.1196963
Iteration 6:   log likelihood = -8.1196963
Iteration 7:   log likelihood = -8.1196963
Iteration 8:   log likelihood = -8.1196963
Iteration 9:   log likelihood = -8.1196963
Iteration 10:  log likelihood = -8.1196963
Iteration 11:  log likelihood = -8.1196963
Iteration 12:  log likelihood = -8.1196963
Iteration 13:  log likelihood = -8.1196963
Iteration 14:  log likelihood = -8.1196963
Iteration 15:  log likelihood = -8.1196963
Iteration 16:  log likelihood = -8.1196963
Iteration 17:  log likelihood = -8.1196963

Cox regression -- no ties

No. of subjects =          595                  Number of obs    =         595
No. of failures =            3
Time at risk    =       644772
                                                LR chi2(0)       =       11.50
Log likelihood  =   -8.1196963                  Prob > chi2      =           .
```

_t	Haz. Ratio	Std. Err.	z	P>|z|	[95% Conf.	Interval]
eitergelenk	1.95e-15	.	.	.	.	.

```
514 . xi: stcox crp

          failure _d:  samerecurrence
    analysis time _t:  (coxend-origin)
              origin:  time dateop
                  id:  id

Iteration 0:   log likelihood = -5.4638318
Iteration 1:   log likelihood = -5.3867421
Iteration 2:   log likelihood = -5.3702623
Iteration 3:   log likelihood =  -5.369119
Iteration 4:   log likelihood = -5.3691121
Refining estimates:
Iteration 0:   log likelihood = -5.3691121

Cox regression -- no ties

No. of subjects =          420                  Number of obs    =         420
No. of failures =            1
Time at risk    =       449309
                                                LR chi2(1)       =        0.19
Log likelihood  =   -5.3691121                  Prob > chi2      =      0.6634
```

_t	Haz. Ratio	Std. Err.	z	P>|z|	[95% Conf.	Interval]
crp	.9884161	.0340004	-0.34	0.735	.9239734	1.057353

```
515 . xi: stcox sehnenresektion

         failure _d:  samerecurrence
   analysis time _t:  (coxend-origin)
             origin:  time dateop
                 id:  id

    Iteration 0:   log likelihood = -13.871301
    Iteration 1:   log likelihood = -13.748361
    Iteration 2:   log likelihood = -13.707092
    Iteration 3:   log likelihood = -13.692368
    Iteration 4:   log likelihood =  -13.68701
    Iteration 5:   log likelihood = -13.685047
    Iteration 6:   log likelihood = -13.684326
    Iteration 7:   log likelihood = -13.684061
    Iteration 8:   log likelihood = -13.683963
    Iteration 9:   log likelihood = -13.683927
    Iteration 10:  log likelihood = -13.683914
    Iteration 11:  log likelihood = -13.683909
    Iteration 12:  log likelihood = -13.683907
    Iteration 13:  log likelihood = -13.683907
    Iteration 14:  log likelihood = -13.683907
    Iteration 15:  log likelihood = -13.683907
    Iteration 16:  log likelihood = -13.683906
    Iteration 17:  log likelihood = -13.683906
    Iteration 18:  log likelihood = -13.683906
    Iteration 19:  log likelihood = -13.683906
    Iteration 20:  log likelihood = -13.683906
    Iteration 21:  log likelihood = -13.683906
    Iteration 22:  log likelihood = -13.683906
    Iteration 23:  log likelihood = -13.683906
    Iteration 24:  log likelihood = -13.683906
    Iteration 25:  log likelihood = -13.683906
    Iteration 26:  log likelihood = -13.683906
    Iteration 27:  log likelihood = -13.683906
    Iteration 28:  log likelihood = -13.683906
    Iteration 29:  log likelihood = -13.683906
    Refining estimates:
    Iteration 0:   log likelihood = -13.683906
    Iteration 1:   log likelihood = -13.683906
    Iteration 2:   log likelihood = -13.683906
    Iteration 3:   log likelihood = -13.683906

    Cox regression -- no ties

    No. of subjects =          595                Number of obs    =         595
    No. of failures =            3
    Time at risk    =       644772
                                                  LR chi2(1)       =        0.37
    Log likelihood  =   -13.683906                Prob > chi2      =      0.5404
```

_t	Haz. Ratio	Std. Err.	z	P>|z|	[95% Conf.	Interval]
sehnenrese~n	6.26e+13	1.21e+21	0.00	1.000	0	.

```
516 . xi: stcox gelenkversteifung

         failure _d:  samerecurrence
   analysis time _t:  (coxend-origin)
             origin:  time dateop
                 id:  id
```

```
Iteration 0:   log likelihood = -13.871301
Iteration 1:   log likelihood = -13.853674
Iteration 2:   log likelihood = -13.847274
Iteration 3:   log likelihood =  -13.84493
Iteration 4:   log likelihood =  -13.84407
Iteration 5:   log likelihood = -13.843753
Iteration 6:   log likelihood = -13.843637
Iteration 7:   log likelihood = -13.843594
Iteration 8:   log likelihood = -13.843578
Iteration 9:   log likelihood = -13.843573
Iteration 10:  log likelihood =  -13.84357
Iteration 11:  log likelihood =  -13.84357
Iteration 12:  log likelihood = -13.843569
Iteration 13:  log likelihood = -13.843569
Iteration 14:  log likelihood = -13.843569
Iteration 15:  log likelihood = -13.843569
Iteration 16:  log likelihood = -13.843569
Iteration 17:  log likelihood = -13.843569
Iteration 18:  log likelihood = -13.843569
Iteration 19:  log likelihood = -13.843569
Iteration 20:  log likelihood = -13.843569
Iteration 21:  log likelihood = -13.843569
Iteration 22:  log likelihood = -13.843569
Iteration 23:  log likelihood = -13.843569
Iteration 24:  log likelihood = -13.843569
Iteration 25:  log likelihood = -13.843569
Iteration 26:  log likelihood = -13.843569
Iteration 27:  log likelihood = -13.843569
Refining estimates:
Iteration 0:   log likelihood = -13.843569
Iteration 1:   log likelihood = -13.843569
Iteration 2:   log likelihood = -13.843569
Iteration 3:   log likelihood = -13.843569
Iteration 4:   log likelihood = -13.843569
Iteration 5:   log likelihood = -13.843569

Cox regression -- no ties

No. of subjects =          595                  Number of obs    =        595
No. of failures =            3
Time at risk    =       644772
                                                LR chi2(1)       =       0.06
Log likelihood  =   -13.843569                  Prob > chi2      =     0.8138
```

_t	Haz. Ratio	Std. Err.	z	P>|z|	[95% Conf.	Interval]
gelenkvers~g	7.25e+13	2.43e+21	0.00	1.000	0	.

```
517 . xi: stcox wundheilungsstoerung

         failure _d:  samerecurrence
   analysis time _t:  (coxend-origin)
             origin:  time dateop
                 id:  id

Iteration 0:   log likelihood = -13.871301
Iteration 1:   log likelihood = -13.605335
Iteration 2:   log likelihood = -13.360908
Iteration 3:   log likelihood = -13.360731
Refining estimates:
Iteration 0:   log likelihood = -13.360731

Cox regression -- no ties

No. of subjects =          595                  Number of obs    =        595
No. of failures =            3
Time at risk    =       644772
                                                LR chi2(1)       =       1.02
Log likelihood  =   -13.360731                  Prob > chi2      =     0.3122
```

_t	Haz. Ratio	Std. Err.	z	P>|z|	[95% Conf.	Interval]
wundheilun~g	**3.927337**	**4.81367**	**1.12**	**0.264**	**.3554635**	**43.39117**

```
518 . xi: stcox i.lappen
i.lappen          _Ilappen_0-4        (naturally coded; _Ilappen_0 omitted)

         failure _d:  samerecurrence
   analysis time _t:  (coxend-origin)
             origin:  time dateop
                 id:  id

Iteration 0:   log likelihood = -13.871301
Iteration 1:   log likelihood = -12.553076
Iteration 2:   log likelihood = -12.473919
Iteration 3:   log likelihood = -12.465882
Iteration 4:   log likelihood = -12.462962
Iteration 5:   log likelihood =  -12.46189
Iteration 6:   log likelihood = -12.461496
Iteration 7:   log likelihood = -12.461351
Iteration 8:   log likelihood = -12.461298
Iteration 9:   log likelihood = -12.461278
Iteration 10:  log likelihood = -12.461271
Iteration 11:  log likelihood = -12.461269
Iteration 12:  log likelihood = -12.461268
Iteration 13:  log likelihood = -12.461267
Iteration 14:  log likelihood = -12.461267
Iteration 15:  log likelihood = -12.461267
Iteration 16:  log likelihood = -12.461267
Iteration 17:  log likelihood = -12.461267
Iteration 18:  log likelihood = -12.461267
Iteration 19:  log likelihood = -12.461267
Iteration 20:  log likelihood = -12.461267
Iteration 21:  log likelihood = -12.461267
Iteration 22:  log likelihood = -12.461267
Iteration 23:  log likelihood = -12.461267
Iteration 24:  log likelihood = -12.461267
Iteration 25:  log likelihood = -12.461267
Iteration 26:  log likelihood = -12.461267
Iteration 27:  log likelihood = -12.461267
Iteration 28:  log likelihood = -12.461267
Iteration 29:  log likelihood = -12.461267
Iteration 30:  log likelihood = -12.461267
Iteration 31:  log likelihood = -12.461267
Iteration 32:  log likelihood = -12.461267
Iteration 33:  log likelihood = -12.461267
Iteration 34:  log likelihood = -12.461267
Iteration 35:  log likelihood = -12.461267
Iteration 36:  log likelihood = -12.461267
Iteration 37:  log likelihood = -12.461267
Iteration 38:  log likelihood = -12.461267
Iteration 39:  log likelihood = -12.461267
Iteration 40:  log likelihood = -12.461267
Iteration 41:  log likelihood = -12.461267
Iteration 42:  log likelihood = -12.461267
Refining estimates:
Iteration 0:   log likelihood = -12.461267

Cox regression -- no ties

No. of subjects =          595                 Number of obs    =          595
No. of failures =            3
Time at risk    =       644772
                                               LR chi2(1)       =         2.82
Log likelihood  =   -12.461267                 Prob > chi2      =       0.0931
```

_t	Haz. Ratio	Std. Err.	z	P>|z|	[95% Conf.	Interval]
_Ilappen_1	8.46e-19	.	.	.	.	.
_Ilappen_2	1	(omitted)				
_Ilappen_3	13.99886	19.81489	1.86	0.062	.873468	224.3563
_Ilappen_4	6.25e-18	.	.	.	.	.

```
519 . xi: stcox samerecurrence

          failure _d:  samerecurrence
    analysis time _t:  (coxend-origin)
              origin:  time dateop
                  id:  id

Iteration 0:   log likelihood = -13.871301
Iteration 1:   log likelihood = -1.7917595
Iteration 2:   log likelihood = -1.7917595
Iteration 3:   log likelihood = -1.7917595
Iteration 4:   log likelihood = -1.7917595
Iteration 5:   log likelihood = -1.7917595
Iteration 6:   log likelihood = -1.7917595
Iteration 7:   log likelihood = -1.7917595
Iteration 8:   log likelihood = -1.7917595
Iteration 9:   log likelihood = -1.7917595
Iteration 10:  log likelihood = -1.7917595
Iteration 11:  log likelihood = -1.7917595
Iteration 12:  log likelihood = -1.7917595
Iteration 13:  log likelihood = -1.7917595
Iteration 14:  log likelihood = -1.7917595
Refining estimates:
Iteration 0:   log likelihood = -1.7917595
Iteration 1:   log likelihood = -1.7917595
Iteration 2:   log likelihood = -1.7917595
Iteration 3:   log likelihood = -1.7917595
Iteration 4:   log likelihood = -1.7917595

Cox regression -- no ties

No. of subjects =          595                Number of obs    =         595
No. of failures =            3
Time at risk    =       644772
                                              LR chi2(1)       =       24.16
Log likelihood  =   -1.7917595                Prob > chi2      =      0.0000
```

_t	Haz. Ratio	Std. Err.	z	P>|z|	[95% Conf.	Interval]
samerecurr~e	3.11e+17	9.34e+24	0.00	1.000	0	.

```
520 . xi: stcox softtissuecoverage

          failure _d:  samerecurrence
    analysis time _t:  (coxend-origin)
              origin:  time dateop
                  id:  id

Iteration 0:   log likelihood = -13.871301
Iteration 1:   log likelihood = -13.834557
Iteration 2:   log likelihood = -13.172335
Iteration 3:   log likelihood = -13.169607
Iteration 4:   log likelihood = -13.169607
Refining estimates:
Iteration 0:   log likelihood = -13.169607

Cox regression -- no ties
```

```
No. of subjects =          595                  Number of obs    =         595
No. of failures =            3
Time at risk    =       644772
                                                LR chi2(1)       =        1.40
Log likelihood  =   -13.169607                  Prob > chi2      =      0.2362

------------------------------------------------------------------------------
          _t | Haz. Ratio   Std. Err.      z    P>|z|     [95% Conf. Interval]
-------------+----------------------------------------------------------------
softtissue~e |   5.216864   6.453428     1.34   0.182     .4617915    58.93498
------------------------------------------------------------------------------

521 . xi: stcox aureus

         failure _d:  samerecurrence
   analysis time _t:  (coxend-origin)
             origin:  time dateop
                 id:  id

Iteration 0:   log likelihood = -13.871301
Iteration 1:   log likelihood = -12.627386
Iteration 2:   log likelihood = -12.549214
Iteration 3:   log likelihood = -12.548026
Iteration 4:   log likelihood = -12.548026
Refining estimates:
Iteration 0:   log likelihood = -12.548026

Cox regression -- no ties

No. of subjects =          595                  Number of obs    =         595
No. of failures =            3
Time at risk    =       644772
                                                LR chi2(1)       =        2.65
Log likelihood  =   -12.548026                  Prob > chi2      =      0.1038

------------------------------------------------------------------------------
          _t | Haz. Ratio   Std. Err.      z    P>|z|     [95% Conf. Interval]
-------------+----------------------------------------------------------------
      aureus |   7.237325   9.124964     1.57   0.116     .6114575    85.66234
------------------------------------------------------------------------------

522 . xi: stcox gramnegative

         failure _d:  samerecurrence
   analysis time _t:  (coxend-origin)
             origin:  time dateop
                 id:  id

Iteration 0:   log likelihood = -13.871301
Iteration 1:   log likelihood =  -13.59531
Iteration 2:   log likelihood = -13.506812
Iteration 3:   log likelihood = -13.475651
Iteration 4:   log likelihood = -13.464363
Iteration 5:   log likelihood = -13.460233
Iteration 6:   log likelihood = -13.458717
Iteration 7:   log likelihood = -13.458159
Iteration 8:   log likelihood = -13.457954
Iteration 9:   log likelihood = -13.457879
Iteration 10:  log likelihood = -13.457851
Iteration 11:  log likelihood = -13.457841
Iteration 12:  log likelihood = -13.457837
Iteration 13:  log likelihood = -13.457836
Iteration 14:  log likelihood = -13.457835
Iteration 15:  log likelihood = -13.457835
Iteration 16:  log likelihood = -13.457835
Iteration 17:  log likelihood = -13.457835
Iteration 18:  log likelihood = -13.457835
Iteration 19:  log likelihood = -13.457835
Iteration 20:  log likelihood = -13.457835
Iteration 21:  log likelihood = -13.457835
Iteration 22:  log likelihood = -13.457835
Iteration 23:  log likelihood = -13.457835
```

```
Iteration 24:  log likelihood = -13.457835
Iteration 25:  log likelihood = -13.457835
Iteration 26:  log likelihood = -13.457835
Iteration 27:  log likelihood = -13.457835
Iteration 28:  log likelihood = -13.457835
Iteration 29:  log likelihood = -13.457835
Iteration 30:  log likelihood = -13.457835
Iteration 31:  log likelihood = -13.457835
Iteration 32:  log likelihood = -13.457835
Iteration 33:  log likelihood = -13.457835
Iteration 34:  log likelihood = -13.457835
Iteration 35:  log likelihood = -13.457835
Refining estimates:
Iteration 0:   log likelihood = -13.457835
Iteration 1:   log likelihood = -13.457835

Cox regression -- no ties

No. of subjects =          595                 Number of obs    =          595
No. of failures =            3
Time at risk    =       644772
                                               LR chi2(1)       =         0.83
Log likelihood  =   -13.457835                 Prob > chi2      =       0.3632
```

_t	Haz. Ratio	Std. Err.	z	P>|z|	[95% Conf.	Interval]
gramnegative	1.86e-16	2.05e-08	-0.00	1.000	0	.

```
523 . xi: stcox strept

         failure _d:  samerecurrence
   analysis time _t:  (coxend-origin)
             origin:  time dateop
                 id:  id

Iteration 0:   log likelihood = -13.871301
Iteration 1:   log likelihood = -13.807142
Iteration 2:   log likelihood =  -13.80641
Iteration 3:   log likelihood =  -13.80641
Refining estimates:
Iteration 0:   log likelihood =  -13.80641

Cox regression -- no ties

No. of subjects =          595                 Number of obs    =          595
No. of failures =            3
Time at risk    =       644772
                                               LR chi2(1)       =         0.13
Log likelihood  =    -13.80641                 Prob > chi2      =       0.7187
```

_t	Haz. Ratio	Std. Err.	z	P>|z|	[95% Conf.	Interval]
strept	1.593478	2.006938	0.37	0.711	.1349841	18.81089

```
524 .
525 . ** MULTIVARIATE COX REGRESSION SAMERECURRENCE**
```

```
526 . *** Nicht möglich, da zu kline Fallzahlen (n=3 mikrobiologische Recurrences)
527 .
528 . ** Zusatz Ilker- Was bringt Ab vor erster OP ? Assoziationen ?
529 . tab abvorher sex, chi col
```

Key
frequency<br>*column percentage*

abvorher	sex 1	2	Total
1	**56**<br>**15.47**	**57**<br>**23.95**	**113**<br>**18.83**
2	**306**<br>**84.53**	**181**<br>**76.05**	**487**<br>**81.17**
Total	**362**<br>**100.00**	**238**<br>**100.00**	**600**<br>**100.00**

Pearson chi2(**1**) = **6.7549** Pr = **0.009**

```
530 . tab abvorher aureus, chi col
```

Key
frequency<br>*column percentage*

abvorher	aureus 0	1	Total
1	**83**<br>**19.67**	**30**<br>**16.85**	**113**<br>**18.83**
2	**339**<br>**80.33**	**148**<br>**83.15**	**487**<br>**81.17**
Total	**422**<br>**100.00**	**178**<br>**100.00**	**600**<br>**100.00**

Pearson chi2(**1**) = **0.6487** Pr = **0.421**

```
531 . tab abvorher gramnegative, chi col
```

Key
frequency<br>*column percentage*

abvorher	gramnegative 0	1	Total
1	**97**<br>**18.58**	**16**<br>**20.51**	**113**<br>**18.83**
2	**425**<br>**81.42**	**62**<br>**79.49**	**487**<br>**81.17**
Total	**522**<br>**100.00**	**78**<br>**100.00**	**600**<br>**100.00**

Pearson chi2(**1**) = **0.1654** Pr = **0.684**

532 . tab abvorher strept, chi col

Key
frequency
column percentage

	strept		
abvorher	0	1	Total
1	**101**<br>**20.00**	**12**<br>**12.63**	**113**<br>**18.83**
2	**404**<br>**80.00**	**83**<br>**87.37**	**487**<br>**81.17**
Total	**505**<br>**100.00**	**95**<br>**100.00**	**600**<br>**100.00**

Pearson chi2(**1**) = **2.8399** Pr = **0.092**

533 . tab abvorher amputation, chi col

Key
frequency
column percentage

	amputation		
abvorher	0	1	Total
1	**104**<br>**19.19**	**9**<br>**15.52**	**113**<br>**18.83**
2	**438**<br>**80.81**	**49**<br>**84.48**	**487**<br>**81.17**
Total	**542**<br>**100.00**	**58**<br>**100.00**	**600**<br>**100.00**

Pearson chi2(**1**) = **0.4619** Pr = **0.497**

534 . tab abvorher softtissuecoverage, chi col

Key
frequency
column percentage

	softtissuecoverage		
abvorher	0	1	Total
1	**100**<br>**18.12**	**13**<br>**27.08**	**113**<br>**18.83**
2	**452**<br>**81.88**	**35**<br>**72.92**	**487**<br>**81.17**
Total	**552**<br>**100.00**	**48**<br>**100.00**	**600**<br>**100.00**

Pearson chi2(**1**) = **2.3230** Pr = **0.127**

535 . tab abvorher failure1, chi col

Key
frequency   *column percentage*

abvorher	failure1 0	1	Total
1	**93** **18.86**	**20** **18.69**	**113** **18.83**
2	**400** **81.14**	**87** **81.31**	**487** **81.17**
Total	**493** **100.00**	**107** **100.00**	**600** **100.00**

Pearson chi2(**1**) = **0.0017** Pr = **0.967**

536 . tab abvorher failure2, chi col

Key
frequency   *column percentage*

abvorher	failure2 0	1	Total
1	**89** **19.43**	**24** **16.90**	**113** **18.83**
2	**369** **80.57**	**118** **83.10**	**487** **81.17**
Total	**458** **100.00**	**142** **100.00**	**600** **100.00**

Pearson chi2(**1**) = **0.4542** Pr = **0.500**

537 . tab abvorher dauerabgrp, chi col

Key
frequency   *column percentage*

abvorher	dauerabgrp 1	2	3	Total
1	**30** **15.79**	**27** **21.09**	**56** **19.86**	**113** **18.83**
2	**160** **84.21**	**101** **78.91**	**226** **80.14**	**487** **81.17**
Total	**190** **100.00**	**128** **100.00**	**282** **100.00**	**600** **100.00**

Pearson chi2(**2**) = **1.7732** Pr = **0.412**

538 . tab abvorher eitergelenk, chi col

Key
frequency<br>*column percentage*

abvorher	eitergelenk 1	2	Total
1	**7**<br>**17.07**	**106**<br>**18.96**	**113**<br>**18.83**
2	**34**<br>**82.93**	**453**<br>**81.04**	**487**<br>**81.17**
Total	**41**<br>**100.00**	**559**<br>**100.00**	**600**<br>**100.00**

Pearson chi2(**1**) = **0.0892** Pr = **0.765**

539 .
540 . save "D:\camillofranziska", replace
(note: file D:\camillofranziska.dta not found)
file D:\camillofranziska.dta saved

541 .
end of do-file
